# Thio- and selenosemicarbazones as antiprotozoal agents against *Trypanosoma cruzi* and *Trichomonas vaginalis*

**DOI:** 10.1080/14756366.2022.2041629

**Published:** 2022-02-23

**Authors:** Alexandra Ibáñez-Escribano, Cristina Fonseca-Berzal, Mónica Martínez-Montiel, Manuel Álvarez-Márquez, María Gómez-Núñez, Manuel Lacueva-Arnedo, Teresa Espinosa-Buitrago, Tania Martín-Pérez, José Antonio Escario, Penélope Merino-Montiel, Sara Montiel-Smith, Alicia Gómez-Barrio, Óscar López, José G. Fernández-Bolaños

**Affiliations:** a Unidad de Parasitología, Departamento de Microbiología y Parasitología, Facultad de Farmacia, Madrid, Spain; b Facultad de Ciencias Químicas, Ciudad Universitaria, Benemérita Universidad Autónoma de Puebla, Puebla, México; c Departamento de Química Orgánica, Facultad de Química, Universidad de Sevilla, Sevilla, Spain; d Escuela Politécnica Superior, Universidad de Sevilla, Sevilla, Spain; e Departamento de Biomedicina y Biotecnología, Facultad de Farmacia, Universidad de Alcalá, Alcalá de Henares, Madrid, Spain; f Institute of Specific Prophylaxis and Tropical Medicine, Medical University of Vienna, Vienna, Austria

**Keywords:** *Trypanosoma cruzi*, *Trichomonas vaginalis*, antiprotozoal agents, thio(seleno)semicarbazones, unspecific cytotoxicity, MoA

## Abstract

Herein, we report the preparation of a panel of Schiff bases analogues as antiprotozoal agents by modification of the stereoelectronic effects of the substituents on N-1 and N-4 and the nature of the chalcogen atom (S, Se). These compounds were evaluated towards *Trypanosoma cruzi* and *Trichomonas vaginalis*. Thiosemicarbazide **31** showed the best trypanocidal profile (epimastigotes), similar to benznidazole (BZ): IC_50_ (**31**)=28.72 μM (CL-B5 strain) and 33.65 μM (Y strain), IC_50_ (BZ)=25.31 μM (CL-B5) and 22.73 μM (Y); it lacked toxicity over mammalian cells (CC_50_ > 256 µM). Thiosemicarbazones **49**, **51** and **63** showed remarkable trichomonacidal effects (IC_50 _=16.39, 14.84 and 14.89 µM) and no unspecific cytotoxicity towards Vero cells (CC_50_ ≥ 275 µM). Selenoisosters **74** and **75** presented a slightly enhanced activity (IC_50_=11.10 and 11.02 µM, respectively). Hydrogenosome membrane potential and structural changes were analysed to get more insight into the trichomonacidal mechanism.

## Graphical Abstract

**Figure:**
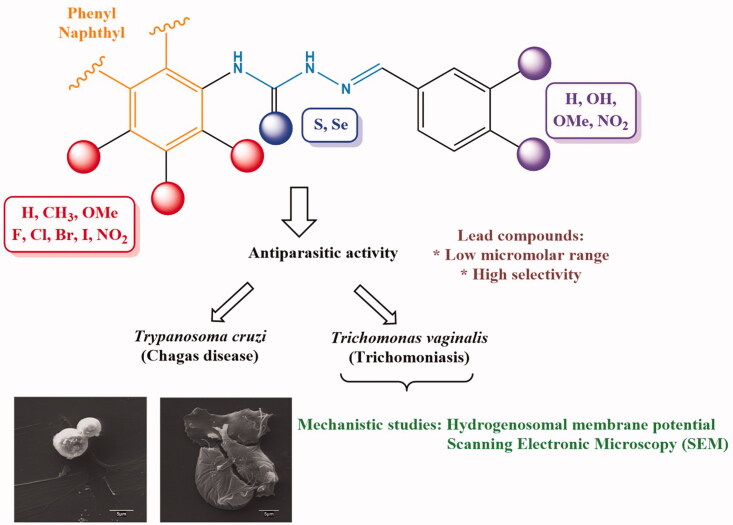

## 1. Introduction

Protozoa are responsible for a large number of severe parasitic diseases in humans, livestock and pets, causing important morbidity and mortality worldwide^1^. This constitutes a burden for health systems in low-medium income tropical and subtropical countries from Africa, Asia and Latin America, where some of these protozooses are endemic^2^. Despite affecting more than one billion people, almost one sixth of the human population, and causing roughly half million deaths per year, many of these diseases are largely ignored, and indeed, some of them are categorised into the so-called Neglected Tropical Diseases (NTDs) group^3^.

Management of diseases caused by pathogenic protozoa is not a simple task^4^. On the one hand, development of successful vaccines is a hitherto unachieved goal^5^; on the other hand, the chemotherapeutic arsenal available so far suffers from important drawbacks: most of them are old drugs that are developing chemoresistance^6^, and are endowed with severe side-effects^7^ and low efficiency^8^. Moreover, their high prices, and complex administration protocols make them unaffordable for underdeveloped countries^9^. Accordingly, the development of new antiprotozoal agents is a hot topic in current Medicinal Chemistry research^10^^,^^11^.

Herein, we have focussed our attention on Chagas disease and trichomoniasis. On the one hand, Chagas disease (aka American trypanosomiasis), discovered by the Brazilian physician Carlos R.J. das Chagas in 1909, is endemic in Latin America, where it affects roughly 7 million people^12^; nevertheless, it is also being spread to USA, Canada, Europe and Australia, because of human migrations^13^, and therefore, becoming a global health problem^14^. Chagas disease is caused by the haemoflagellate protozoan *Trypanosoma cruzi*, whose transmission to humans naturally takes place by contact with faeces or urine of infected triatomine insects. The infection can also be transmitted by non-vectorial routes, such as the iatrogenic and the congenital one^12^. The life-cycle of the parasite involves three stages: epimastigotes (extracellular and replicative form found in the intestine of the vector), amastigotes (intracellular and proliferative form of the vertebrate host) and trypomastigotes (extracellular and non-replicative state found in the bloodstream)^15^^,^^16^. Currently, there are only two available drugs for the specific treatment of Chagas disease: benznidazole (BZ), a nitroimidazole, and nifurtimox, a nitrofurane both showing some disadvantages (e.g., low efficacy in the chronic phase, adverse effects and parasite drug resistance) that constitute one of the main drawbacks of this parasitosis^17^.

Cruzipain, the main papain-like cysteine peptidase in *T. cruzi* is currently considered as a validated therapeutic target against Chagas disease^18^, and thus compounds inhibiting such enzyme constitute an interesting alternative to classical antichagasic drugs. Some thio-^19^^‒^^21^ and selenosemicarbazones^22^ have been reported to be good inhibitors of such enzymes.

On the other hand, regarding the four curable sexually-transmitted infections (STI), three of them are caused by bacteria (chlamydia, gonorrhoea and syphilis), whereas the fourth one (trichomoniasis) is caused by a flagellated protozoan. In this context, a recent report has indicated^23^ that within population ranging from 15 to 49 years, 376.4 million new cases of STI were estimated to appear each year, among which, 156.0 million cases of urogenital trichomoniasis are found. Human trichomoniasis, recently categorised as a neglected parasitic infection (NPI)^24^, not only accounts for more than 40% of curable STI, but also represents a severe health risk^25^, as it increases the susceptibility to HIV, human papilloma virus (HPV), or herpes simplex virus (HSV) infections, as well as cervical and prostate cancers^26^. In this sense, some authors have demonstrated the positive impact of trichomoniasis treatment on the prevention of HIV transmission^27^. Therefore, as the development of these neoplasia seems to be associated with the inflammatory response induced by the parasite^26^, its diagnosis and treatment could also reduce the risk of their subsequent development. Moreover, and due to the fact that trichomoniasis is not a notifiable STI, with a high number of asymptomatic patients, epidemiologic data might be underestimated^28^. Currently, there are only two drugs available for the treatment of trichomoniasis^29^, both of them being nitroimidazole derivatives: metronidazole (MTZ), discovered in 1959, and still the first drug of choice, and tinidazole, approved in 2004. Due to the scarce number of commercialised drugs against trichomoniasis, when drug resistance, side-effects or hipersensibility to 5-nitroimidazoles emerge, no alternative treatments are available^29^.

Herein, we have accomplished the preparation of an ample panel of thiosemicarbazones with the aim of developing antiprotozoal agents with a different mechanism of action than that exhibited by nitroheterocyclic derivatives. This type of Schiff bases analogues has been reported to be endowed with a broad spectrum of relevant biological properties, like inhibitors of aldose reductase^30^, tyrosinase^31^, urease^32^ cholinesterases^33^, or β-amyloid aggregation^34^, and also as antimicrobial^35^, or anticancer agents^36^^‒^^38^, the latter being the most widely studied. Although there are some reports on the use of thiosemicarbazones as antiprotozoal agents against *Toxoplasma gondii*^39^^‒^^41^ or *T. cruzi*^42^^‒^^45^, studies on *T. vaginalis* are very scarce and limited to nitrofurane derivatives and bisthiosemicarbazones^46–49^.

## 2. Materials and methods

### 2.1. General procedures

TLCs were performed using aluminium-coated sheets (Merck 60 F_254_), 0.25 mm gel thickness. Each eluant is indicated in the experimental procedures. Spots were visualised by UV light (λ = 254 nm), and by charring with 10% ethanolic vainillin containing 1% H_2_SO_4_, or with 5% ethanolic phosphomolibdic acid.

Column chromatography purifications were performed using silica gel stationary phase (Merck 60, particle size 40‒63 µm), eluting by gravity, or with a mild pressure. Eluants are indicated in each case.

NMR spectra were registered in the Centro de Investigación, Tecnología e Innovación de la Universidad de Sevilla (CITIUS), using Bruker Avance III 300 spectrometer (300.1 MHz for ^1^H, 75.5 MHz for ^13 ^C), using the solvent indicated in each case. Chemical shifts (δ) are expressed in ppm, and coupling constants (*J*) in Hz. Residual signals from the solvent are used as internal references for the calibration. Mass spectra were registered using a QExactive™ spectrometer, using Electrospray Ionisation (ESI), and calibrated using the Pierce^TM^ LTQ Velos ESI Positive Ion Calibration Solution (ThermoFisher Scientific).

### 2.2. Biological assessments

General procedures for the biological assays accomplished herein can be found in the Supporting Information.

### 2.3. Chemistry

General procedures for the preparation of isothiocyanates **11**‒**20**, thiosemicarbazides **22**‒**30**, thiosemicarbazones **36**‒**65** and selenosemicarbazones **74**, **75**, together with spectroscopic characterisation of new compounds can be found in the Supporting Information.

## 3. Results and discussion

### 3.1. Chemistry

**Figure 1.**
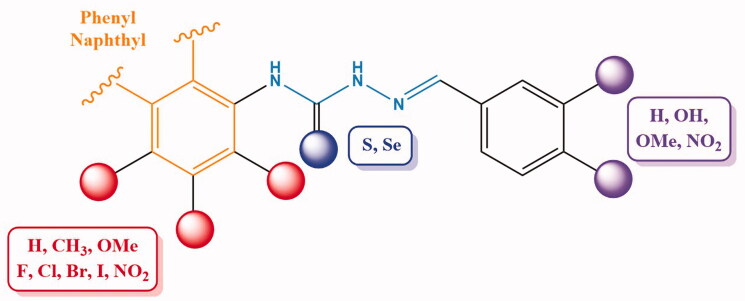
General structure for the ntiprotozoal thio(seleno)semicarbazones prepared herein.

Considering the variety of biological properties exhibited by thiosemicarbazones, including some antiprotozoal activities, we have accomplished the preparation of an ample panel of Schiff bases analogues with the aim of targeting protozoal-mediated diseases like Chagas disease, or trichomoniasis. For this purpose, we envisioned the general structure depicted in Figure 1: an aromatic residue (phenyl, naphthyl) is located on position N-4 of the thiosemicarbazone, which in turn incorporates substituents with different stereoelectronic properties, like methyl, methoxy, halogens, or NO_2_. Moreover, the second aromatic motif, on position N-1 of the Schiff base is decorated with a different number of substituents, including free and masked phenolics, and NO_2_, which can modulate both the bioavailability and activity of the compounds.

We have also considered the isosteric replacement of sulphur with selenium; our group has previously prepared a plethora of organoselenium derivatives exhibiting interesting redox properties^50^^,^^51^, together with strong antiproliferative activities^37^^,^^38^^,^^52‒55^.

**Scheme 1.**
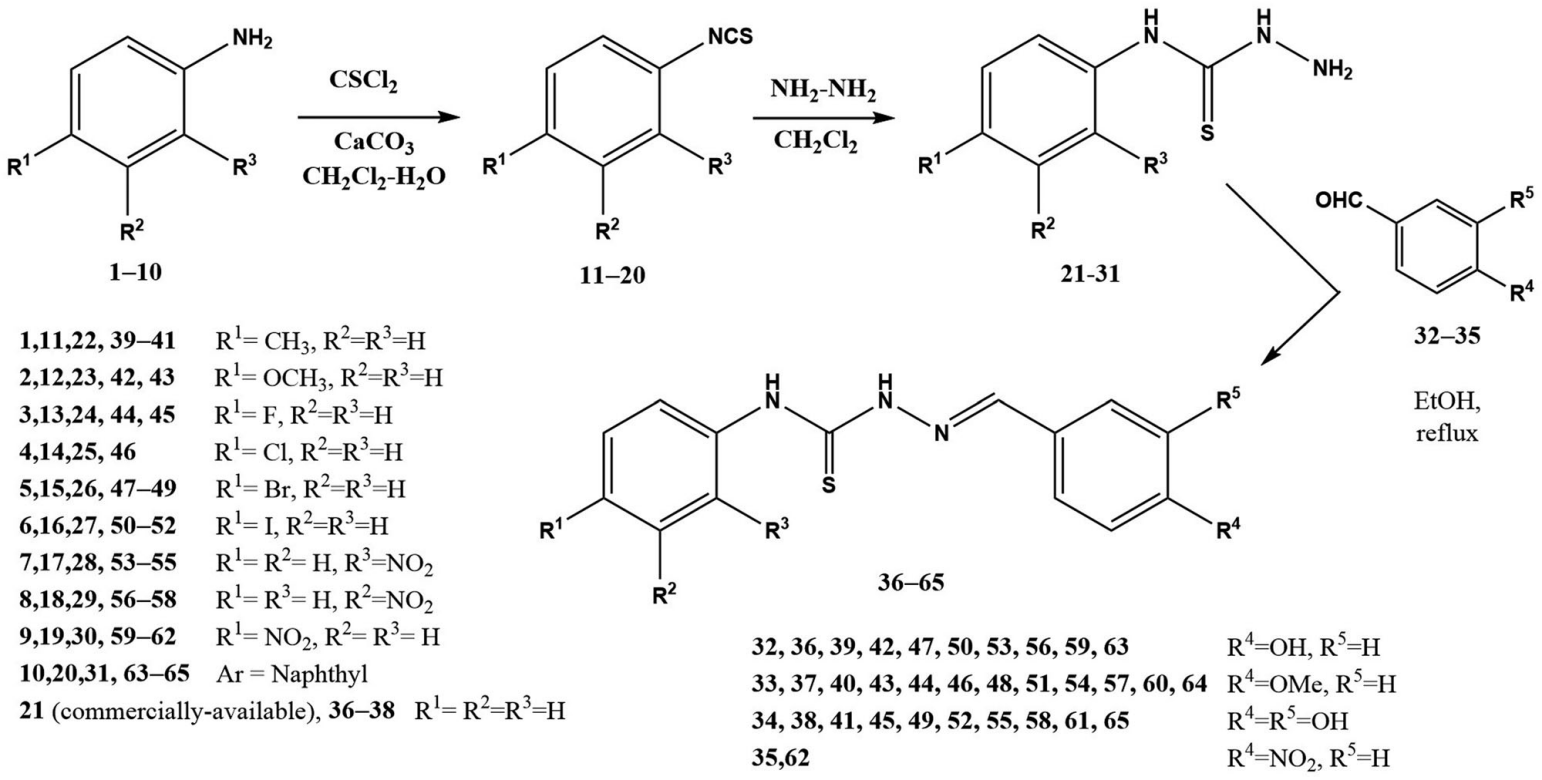
Preparation of thiosemicarbazones **36**‒**65.**

The synthetic approach used for accessing thiosemicarbazones is depicted in Scheme 1. Thus, treatment of commercially available anilines **1**‒**10** with thiophosgene as the thionating agent afforded isothiocyanates **11**‒**20**, which in turn were transformed into target thiosemicarbazones **36**‒**65** upon subsequent treatment with hydrazine, followed by condensation of intermediate thiosemicarbazides **21**‒**31** with the corresponding aldehydes **32**‒**35**. Final compounds precipitated in the medium; ^13 ^C-NMR resonances at 175‒177 ppm (C = S) and at 143‒146 ppm (C = N) confirmed their structures.

**Scheme 2.**
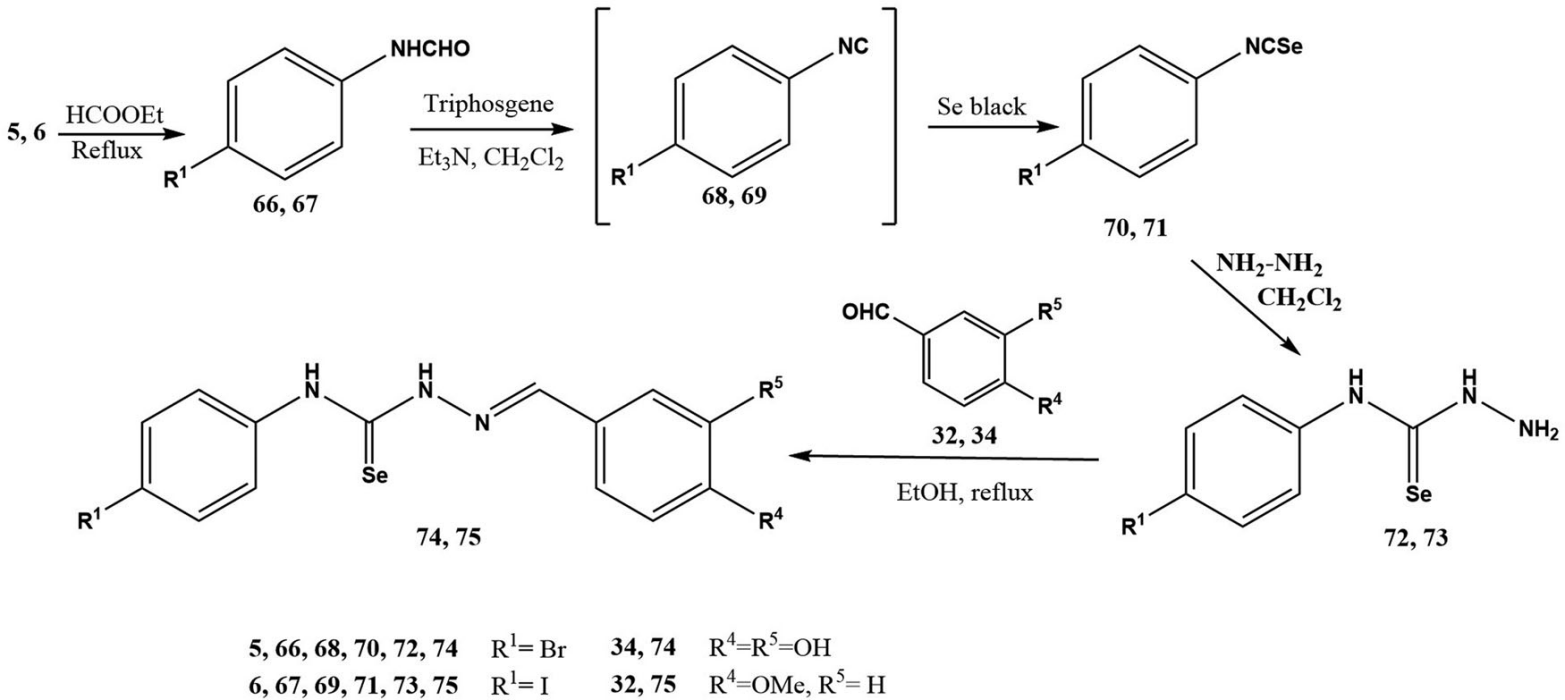
Preparation of selenosemicarbazones **72**, **73.**

Selenosemicarbazones **74**, **75** were obtained using a similar synthetic pathway (Scheme 2); the main difference is the access to the key isoselenocyanates **70**, **71**. They were obtained using a methodology developed in our group^56^^,^^57^ for the preparation of alkyl- and aryl isoselenocyanates, using triphosgene as a safe substitute for hazardous phosgene in the dehydration of formamides **66**, **67** to furnish transient isocyanides **68**, **69**, which underwent addition of elemental black selenium.

### 3.2. Biological assays

#### 3.2.1. In vitro evaluation of anti-T. cruzi activity

Trypanocidal activity was evaluated by following a sequential screening procedure in which all compounds were primarily tested over *T. cruzi* epimastigotes of the drug-sensitive CL-B5 *lacZ* strain (DTU TcVI)^58^^,^^59^. Then, compounds whose trypanocidal profiles were similar to that of the reference drug BZ, were assayed over such an extracellular form of the moderately drug-resistant Y strain (DTU TcII)^60^. Only those compounds with selectivity indexes (SI) on epimastigotes similar or higher than that of BZ, were moved to a more specific assay against the replicative and intracellular forms of the parasite (amastigotes)^58^^,^^59^. Table 1 depicts the results of trypanocidal activity shown by parent thiosemicarbazides (**23**‒**25**, **27‒31**) and the corresponding thiosemicarbazones (**36**‒**65**).

**Table 1.** *In vitro* activity against *Trypanosoma cruzi* CL-B5 (DTU TcVI) and Y (DTU TcII) parasites and unspecific cytotoxicity on L929 and J774 cells, expressed as IC_50_ and CC_50_ values, respectively. Selectivity indexes (SI) for each strain and form are also calculated.

Compound	IC_50_ (µM)^a^ CL-B5 epimastigotes	CC_50_ (µM)^a^ L929 cells	SI^b^ CL-B5 epi	IC_50_ (µM)^a^ Y epimastigotes	CC_50_ (µM)^a^ J774 cells	SI^b^ Y epi	IC_50_ (µM)^a^ CL-B5 amastigotes	SI^b^ CL-B5 ama
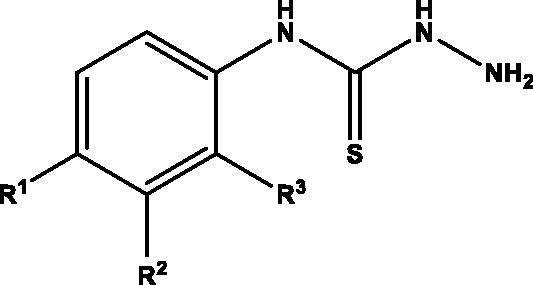								
**23** (R^1^=OMe, R^2^=R^3^=H)	53.51 ± 12.98	>256	>4.78	59.91 ± 10.43	>256	>4.27	−	−
**24** (R^1^=F, R^2^=R^3^=H)	58.80 ± 8.77	>256	>4.35	97.26 ± 3.45	>256	>2.63	−	−
**25** (R^1^=Cl, R^2^=R^3^=H)	44.17 ± 13.61	67.66 ± 3.16	1.53	−	−	−	−	−
**27** (R^1^=I, R^2^=R^3^=H)	49.76 ± 14.25	50.99 ± 8.88	1.02	−	−	−	−	−
**28** (R^1^=R^2^=H, R^3^=NO_2_)	44.15 ± 5.38	210.34 ± 15.34	4.76	−	−	−	−	−
**29** (R^1^=R^3^=H, R^2^=NO_2_)	79.86 ± 15.40	205.76 ± 7.25	2.58	−	−	−	−	−
**30** (R^1^=NO_2_, R^2^=R^3^=H)	72.49 ± 1.23	23.58 ± 2.84	0.33	−	−	−	−	−
**31** (Ar = Naphthyl)	28.72 ± 4.61	>256	>8.91	33.65 ± 5.72	>256	>7.61	39.66 ± 9.91	>6.45
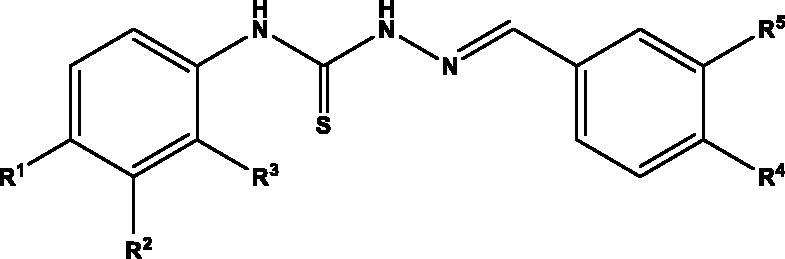								
R^1^=R^2^= R^3^=H								
** 36** R^4^=OH, R^5^=H	70.88 ± 10.96	46.29 ± 0.15	0.65	−	−	−	−	−
** 37** R^4^=OMe R^5^=H	39.79 ± 6,43	>256	>6.43	85.10 ± 17.15	>256	>3.01	−	−
** 38** R^4^=R^5^=OH	15.34 ± 2.22	45.19 ± 0.58	2.95	−	−	−	−	−
R^1^=Me, R^2^=R^3^=H								
** 39** R^4^=OH, R^5^=H	39.58 ± 7.08	60.99 ± 3.72	1.54	−	−	−	−	−
** 40** R^4^=OMe R^5^=H	>256	>256	ND^c^	−	−	−	−	−
** 41** R^4^=R^5^=OH	118.61 ± 7.68	20.02 ± 1.87	0.17	−	−	−	−	−
R^1^=OMe, R^2^=R^3^=H								
** 42** R^4^=OH, R^5^=H	49.19 ± 9.97	43.31 ± 16.61	0.88	−	−	−	−	−
** 43** R^4^=OMe R^5^=H	26.75 ± 2.68	>256	>9.57	55.57 ± 16.30	>256	>4.61	39.04 ± 14.33	>6.56
R^1^=F, R^2^=R^3^=H								
** 44** R^4^=OMe R^5^=H	>256	>256	ND	−	−	−	−	−
** 45** R^4^=R^5^=OH	97.38 ± 5.33	30.47 ± 3.93	0.31	−	−	−	−	−
R^1^=Cl, R^2^=R^3^=H								
** 46** R^4^=OMe R^5^=H	30.53 ± 4.10	49.72 ± 3.76	1.63	−	−	−	−	−
R^1^=Br, R^2^=R^3^=H								
** 47** R^4^=OH, R^5^=H	42.79 ± 12.01	66.96 ± 5.43	1.56	−	−	−	−	−
** 48** R^4^=OMe R^5^=H	>256	>256	ND	−	−	−	−	−
** 49** R^4^=R^5^=OH	104.5 ± 15.71	16.71 ± 0.70	0.16	−	−	−	−	−
R^1^=I, R^2^=R^3^=H								
** 50** R^4^=OH, R^5^=H	22.80 ± 1.61	41.44 ± 8.36	1.82	−	−	−	−	−
** 51** R^4^=OMe R^5^=H	>256	>256	ND^c^	−	−	−	−	−
** 52** R^4^=R^5^=OH	69.37 ± 4.73	20.82 ± 7.92	0.30	−	−	−	−	−
R^1^=R^2^=HR^3^=NO_2_								
** 53** R^4^=OH, R^5^=H	21.77 ± 2.98	34.89 ± 8.65	1.60	−	−	−	−	−
** 54** R^4^=OMe R^5^=H	210.40 ± 14.54	>256	>1.22	−	−	−	−	−
** 55** R^4^=R^5^=OH	41.99 ± 9.06	21.40 ± 4.12	0.51	−	−	−	−	−
R^1^=R^3^=HR^2^=NO_2_								
** 56** R^4^=OH, R^5^=H	36.85 ± 3.84	28.81 ± 3.25	0.78	−	−	−	−	−
** 57** R^4^=OMe R^5^=H	22.96 ± 6.33	>256	>11.15	>256	>256	ND	22.27 ± 4.20	>11.49
** 58** R^4^=R^5^=OH	33.79 ± 7.59	11.98 ± 1.42	0.35	−	−	−	−	−
R^1^=NO_2_ R^2^=R^3^=H								
** 59** R^4^=OH, R^5^=H	76.37 ± 9.91	29.44 ± 2.52	0.39	−	−	−	−	−
** 60** R^4^=OMe R^5^=H	>256	>256	ND	−	−	−	−	−
** 61** R^4^=R^5^=OH	110.56 ± 14.83	5.37 ± 0.30	0.05	−	−	−	−	−
** 62** R^4^=NO_2_ R^5^=H	>256	53.50 ± 4.82	<0.21	−	−	−	−	−
Ar = Naphthyl								
** 63** R^4^=OH R^5^=H	53.65 ± 11.28	31.14 ± 9.21	0.58	−	−	−	−	−
** 64** R^4^=OMe R^5^=H	>256	>256	ND	−	−	−	−	−
** 65** R^4^=R^5^=OH	70.23 ± 6.99	28.79 ± 7.77	0.41	−	−	−	−	−
**Benznidazole**	25.31 ± 1.63	>256	>10.11	22.73 ± 1.82	>256	>11.26	0.47 ± 0.09	>544.68

^a^Results are expressed as the mean ± SD of three independent experiments (*n* = 3).

^b^SI CL-B5 epi = CC_50_ L929/IC_50_ CL *lacZ* epimastigotes, SI Y epi = CC_50_ J774/IC_50_ Y epimastigotes and SI CL-B5 ama = CC_50_ L929/IC_50_ CL *lacZ* amastigotes.

^c^ND: not determined.

As it can be seen, thiosemicarbazides **23** and **31** and thiosemicarbazones **37**, **43** and **57** were active on CL-B5 epimastigotes, showing selectivity indexes (SI) ranging from >4.78 to >11.15. According to these results, they were tested on Y strain epimastigotes: three of them displayed slightly lower activity against moderately drug-resistant parasites, and only **23** and **31** showed similar trypanocidal profiles over both DTU TcVI and TcII strains. As depicted in Table 1, none of these molecules triggered toxic effects on mammalian cells, either phagocytic or non-phagocytic (CC_50_ >256 µM). In fact, previous studies introduce the capability of different series of thiosemicarbazone-based compounds to inhibit epimastigotes growth, proposing the interaction of these molecules with parasite proteases (i.e., cruzipain, one validated target for Chagas disease) as responsible of such an effect^19^^‒^^21^. Improved inhibition of cruzipain (low nanomolar range) was reported^22^ for the isosteric replacement of sulphur with selenium, what was claimed to be responsible for the antiparasitic activity of such selenosemicarbazones (epimastigotes and intracellular amastigotes).

Regarding the activity on CL-B5 amastigotes, compounds **31**, **43** and **57** were the only derivatives assayed against the replicative and intracellular form of the parasite, according to their SI on CL-B5 epimastigotes: SI **31 **>** **8.91, SI **43 **>** **9.57 and SI **57 **>** **11.15. Unfortunately, none of these derivatives were as active as BZ, with IC_50_ values on amastigotes ranging from 22.27 to 39.66 µM. This reduction in the parasite burden of infected cells could also occur because of cruzipain inhibition: the cysteine protease, also present in the intracellular form of *T. cruzi*, has been usually proposed as putative target of thiosemicarbazones^61^^,^^62^.

#### 3.2.2. In vitro evaluation of anti-*Trichomonas vaginalis* activity

Activity against *T. vaginalis* was evaluated following the sequential flow chart protocol of drug screening in which the most active molecules against the parasite are subsequently evaluated against Vero cells to determine their selectivity indexes (SI)^63^. The antiparasitic activity of thiosemicarbazides **23**‒**25**, **27‒31** and thiosemicarbazones **36**‒**65** is shown in Table 2. Activity of all the compounds was lower than that of the reference drug MTZ (IC_50_=2.68 µM). However, almost 37% of all the synthetic molecules exhibited from moderate to good trichomonacidal activity with an IC_50_≤50 µM and were screened against mammalian cell lines.

**Table 2.** *In vitro* activity against *Trichomonas vaginalis* (IC_50_), unspecific cytotoxicity of the most promising molecules against Vero cells (CC_50_) and selectivity indexes (SI).

Compound	**IC_50_ (µM)** ^a^	CC_50_ (µM)	**SI** ^b^
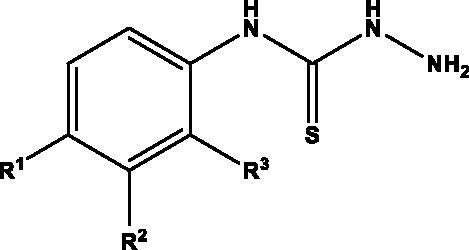			
**23** (R^1^=OMe, R^2^=R^3^=H)	255.70 [205.26–348.10]	–	–
**24** (R^1^=F, R^2^=R^3^=H)	200.49 [134.86–366.51]	–	–
**25** (R^1^=Cl, R^2^=R^3^=H)	49.19 [30.46–77.32]	>300	>6.10
**27** (R^1^=I, R^2^=R^3^=H)	75.69 [57.77–102.71]	–	–
**28** (R^1^=R^2^=H, R^3^=NO_2_)	>300	–	–
**29** (R^1^=R^3^=H, R^2^=NO_2_)	19.14 [14.92–23.52]	>300	>15.67
**30** (R^1^=NO_2_, R^2^=R^3^=H)	105.92 [63.13–236.25]	–	–
**31** (Ar = Naphthyl)	30.20 [11.76–55.08]	>300	>9.93
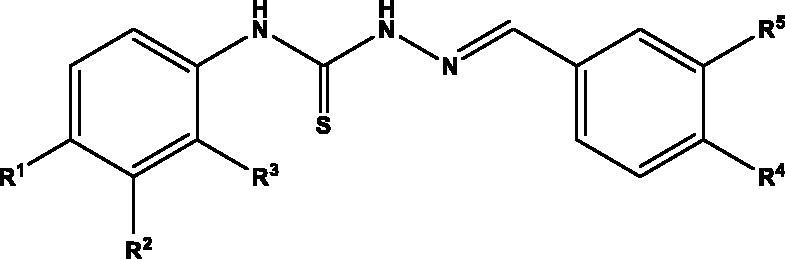			
R^1^=R^2^= R^3^=H			
** 36** R^4^=OH, R^5^=H	74.01 [60.18–92.68]	–	–
** 37** R^4^=OMe R^5^=H	167.29 [116.44–283.25]	–	–
** 38** R^4^=R^5^=OH	88.59 [65.64–125.65]	–	–
R^1^=Me, R^2^=R^3^=H			
** 39** R^4^=OH, R^5^=H	66.10 [43.85–103.72]	–	–
** 40** R^4^=OMe R^5^=H	225.96 [118.97–1084.50]	–	–
** 41** R^4^=R^5^=OH	80.61 [69.32–94.63]	–	–
R^1^=OMe, R^2^=R^3^=H			
** 42** R^4^=OH, R^5^=H	64.79 [55.04–76.69]	–	–
** 43** R^4^=OMe R^5^=H	257.50 [141.94–834.94]	–	–
R^1^=F, R^2^=R^3^=H			
** 44** R^4^=OMe R^5^=H	>300	–	–
** 45** R^4^=R^5^=OH	226.35 [192.65–275.96]	–	–
R^1^=Cl, R^2^=R^3^=H			
** 46** R^4^=OMe R^5^=H	32.27 [21.71–47.99]	>300	>9.30
R^1^=Br, R^2^=R^3^=H			
** 47** R^4^=OH, R^5^=H	52.36 [35.6–77.09]	–	–
** 48** R^4^=OMe R^5^=H	55.14 [47.61–63.92]	–	–
** 49** R^4^=R^5^=OH	16.39 [0.08–28.72]	>300	>18.30
R^1^=I, R^2^=R^3^=H			
** 50** R^4^=OH, R^5^=H	42.25 [36.30–48.52]	>300	>7.10
** 51** R^4^=OMe R^5^=H	14.85 [5.94–24.24]	>300	>20.20
** 52** R^4^=R^5^=OH	24.89 [11.95–41.25]	>300	>12.05
R^1^=R^2^=HR^3^=NO_2_			
** 53** R^4^=OH, R^5^=H	92.80 [81.78–103.30]	–	–
** 54** R^4^=OMe, R^5^=H	34.20 [29.47–39.39]	>300	>8.77
** 55** R^4^=R^5^=OH	132.56 [120.93–146.15]	–	–
R^1^=R^3^=HR^2^=NO_2_			
** 56** R^4^=OH, R^5^=H	24.28 [12.49–38.45]	168.93 [127.03–239–12]	7.00
** 57** R^4^=OMe R^5^=H	51.91 [31.66–85.57]	–	–
** 58** R^4^=R^5^=OH	73.41 [50.52–110.14]	–	–
R^1^=NO_2_ R^2^=R^3^=H			
** 59** R^4^=OH, R^5^=H	35.95 [29.98–42.63]	254.15 [170.16–725.83]	7.07
** 60** R^4^=OMe R^5^=H	155.17 [93.43–395.10]	–	–
** 61** R^4^=R^5^=OH	127.98 [77.11–294.93]	–	–
** 62** R^4^=NO_2_ R^5^=H	151.81 [112.95–209.64]	–	–
Ar = Naphthyl			
** 63** R^4^=OH R^5^=H	14.89 [11.51–18.30]	274.75 [193.19–562.70]	18.45
** 64** R^4^=OMe R^5^=H	30.46 [17.60–46.35]	>300	>9.85
** 65** R^4^=R^5^=OH	20.14 [16.37–24.03]	174.81 [110.58–371.96]	>8.68
**Metronidazole**	2.68 [2.37–3.03]	>300	>111

IC_50_ and CC_50_ were calculated with growth inhibition values showing a standard deviation of less than 10%.

–: Not evaluated against Vero cells owing to the low trichomonacidal activity.

^a^Results in brackets refer to 95% confidence interval.

^b^Selectivity indexes SI = CC_50_ Vero cells/IC_50_ *T. vaginalis*.

In particular, four molecules displayed remarkable activity against *T. vaginalis*, namely thiosemicarbazide **29**, and thiosemicarbazones **49**, **51** and **63**, with IC_50_ values ranging from 14.85‒19.14 µM, and SI > 15.7.

It is important to highlight that most of the thiosemicarbazones synthetised lacked unspecific cytotoxicity at the highest concentration evaluated towards Vero cells. These results are in agreement with the phenylthiosemicarbazones evaluated by Gomes *et al.*^64^ against *T. gondii*, which show cytotoxic effect on Vero cells between 300 to 700 µM. Also, the results published by Merlino *et al.*^65^ refute the low cytotoxic profile of this scaffold showing a low non-specific cytotoxicity on human red blood cells and J-774 mouse macrophages.

In this context, due to the promising trichomonacidal profile and the absence of unspecific cytotoxicity of the most potent derivatives, these molecules can be considered as excellent candidates for further studies. According to this, two selenium compounds (i.e., **74** and **75**) were prepared as the isosters of the two best trichomonacidal compounds evaluated previously (derivatives **49** and **51**, respectively). The activity of these selenosemicarbazones on *T. vaginalis* was slightly enhanced in comparison with that of their sulphur counterparts. Curiously, both derivatives exhibit a similar trichomonacidal effect: IC_50_ **49**_ _= 11.10 µM and IC_50_ **51**_ _=11.02 µM.

Although thiosemicarbazone derivatives **49** and **51** did not exhibit unspecific cytotoxicity, their selenium analogue **74** presented a low cytotoxicity effect at the highest concentrations evaluated against Vero cells (CC_50_ = 114.50 µM), while **75** did not present toxic effects at the maximum concentration tested. Moreover, SI observed in both compounds continues being higher than 10, which demonstrates their specific antiparasitic profile.

According to the trichomonacidal profile, only a few *in vitro* studies based thiosemicarbazones of 5-nitrothiophene-2-carboxaldehyde^46^, or bis (thiosemicarbazone) and bis(4-methylthiosemicarbazone)^49^ have been evaluated against *T. vaginalis*. However, the great structural difference between these molecules and the thio(seleno)semicarbazones prepared herein, makes it not possible to compare the antiparasitic effects.

##### 3.2.2.1. Mechanistic study of the anti-trichomonas activity

In order to get more insight into the anti-trichomonas mode of action (MoA) of title thiosemicarbazones, we have analysed the alteration of the hydrogenosome membrane potential and structural changes in the protozoan; the two more promising compounds (thiosemicarbazones **51** and **63**) were included in this study.

Thus, to determine if the hydrogenosome could be involved in their mode of action, alterations in the membrane potential of this organelle were studied. Membrane potential is indicative of the correct functioning of the organelle, which is essential for parasite survival and also a key aspect for the activation of 5-nitroimidazoles. For this purpose, JC-1 dye (5,5′,6,6′-tetrachloro-1,1′,3,3′-tetraethylbenzimidazolocarbocyanine iodide) was used; this compound aggregates inside the mitochondria or hydrogenosomes in healthy cells, being detected by fluorescence at 590 nm. However, alterations in the membrane potential of the organelle preclude the agglutination of JC-1, which remains as a monomer in the cytoplasm with fluorescence emission at 535 nm.

**Figure 2.**
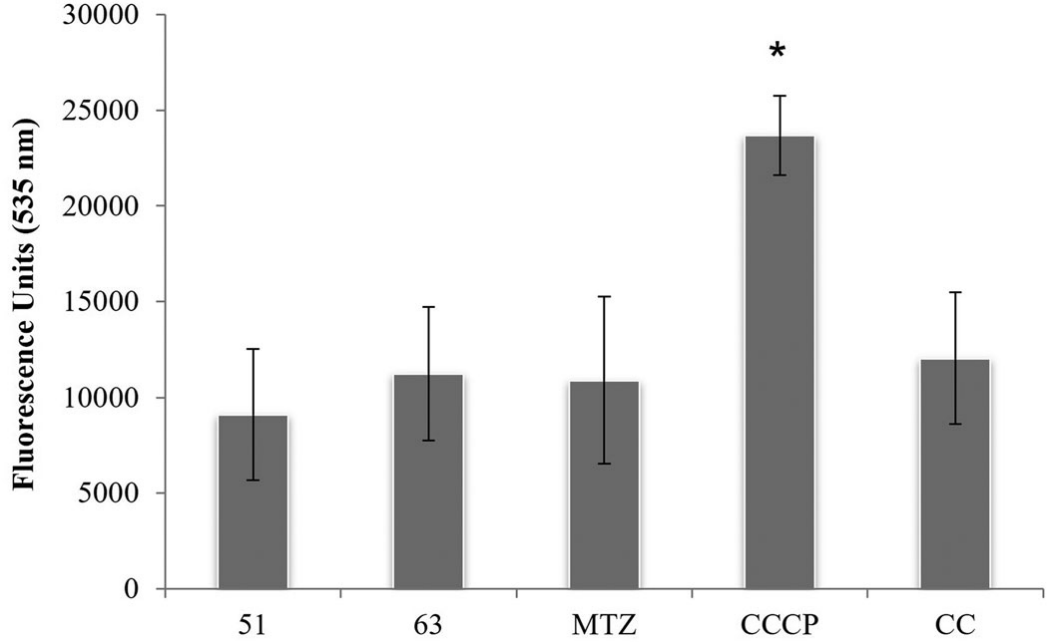
Plots of fluorescence units at 535 nm after addition of JC-1. MTZ (metronidazole), CCCP (*m*-chlorocarbonylcyanide phenylhydrazone) and CC (culture control). Data indicate mean ± SD of three independent experiments (*n* = 3). Data that are significantly different from control experiment are marked with an asterisk (*) (ρ < 0.05).

Figure 2 depicts results of this assay after 4 h of incubation with derivatives **51** and **63** at 100 µM concentration; MTZ (24 µM) was included for comparison.

Results demonstrate that neither the tested thiosemicarbazides nor the reference drug alter the hydrogenosome membrane potential of the parasite. Only CCCP (positive control) showed a significant difference (*t*-Student) with the others, suggesting a charge deregulation and thus, disturbance in the hydrogenosome.

**Figure 3.**
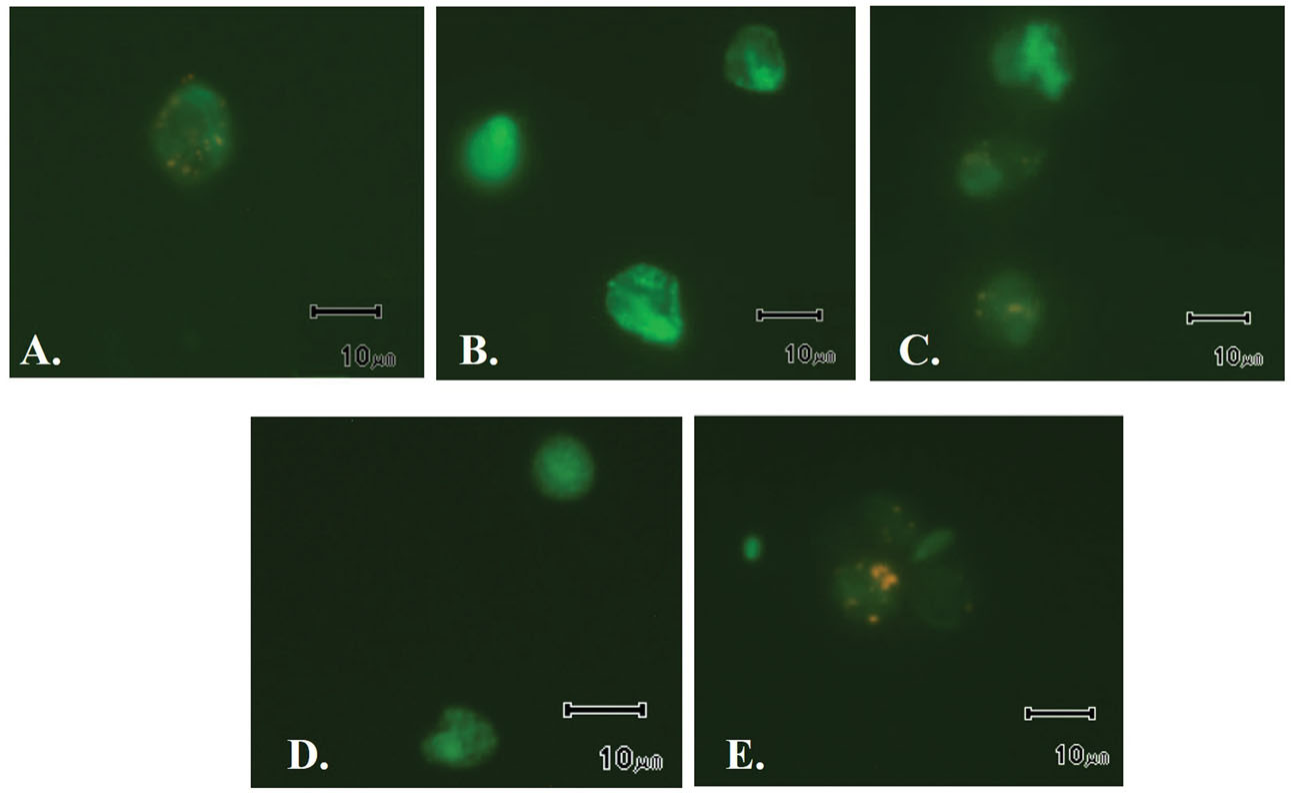
Fluorescence emitted by JC-1 in hydrogenosome. A. Control. B. Positive control (treatment with the uncoupler agent CCCP). C. Treatment with MTZ. D. Treatment with **51**. E. Treatment with **63**.

These data are in agreement with images obtained from fluorescence microscopy. Figure 3 shows that uncoupler agent CCCP (Figure 3(B)) provokes a disturbance in the hydrogenosome membrane potential, as indicated by fluorescence distribution throughout the trophozoite body, which is much more intense and delocalised. However, for the rest of images − control (Figure 3(A)), and *T. vaginalis* treated with MTZ (Figure 3(C)), compound **51** (Figure 3(D)) and **63** (Figure 3(E)) − fluorescence restricted to certain regions of the parasite is observed. In other words, the good physiological condition of this organelle is demonstrated with the fluorescence results observed in Figure 2 and corroborated in the microscope. The accumulation of JC-1 in these organelles is clearly observed in growth controls and trophozoites treated with both compounds as reflects Figure 3.

**Figure 4.**
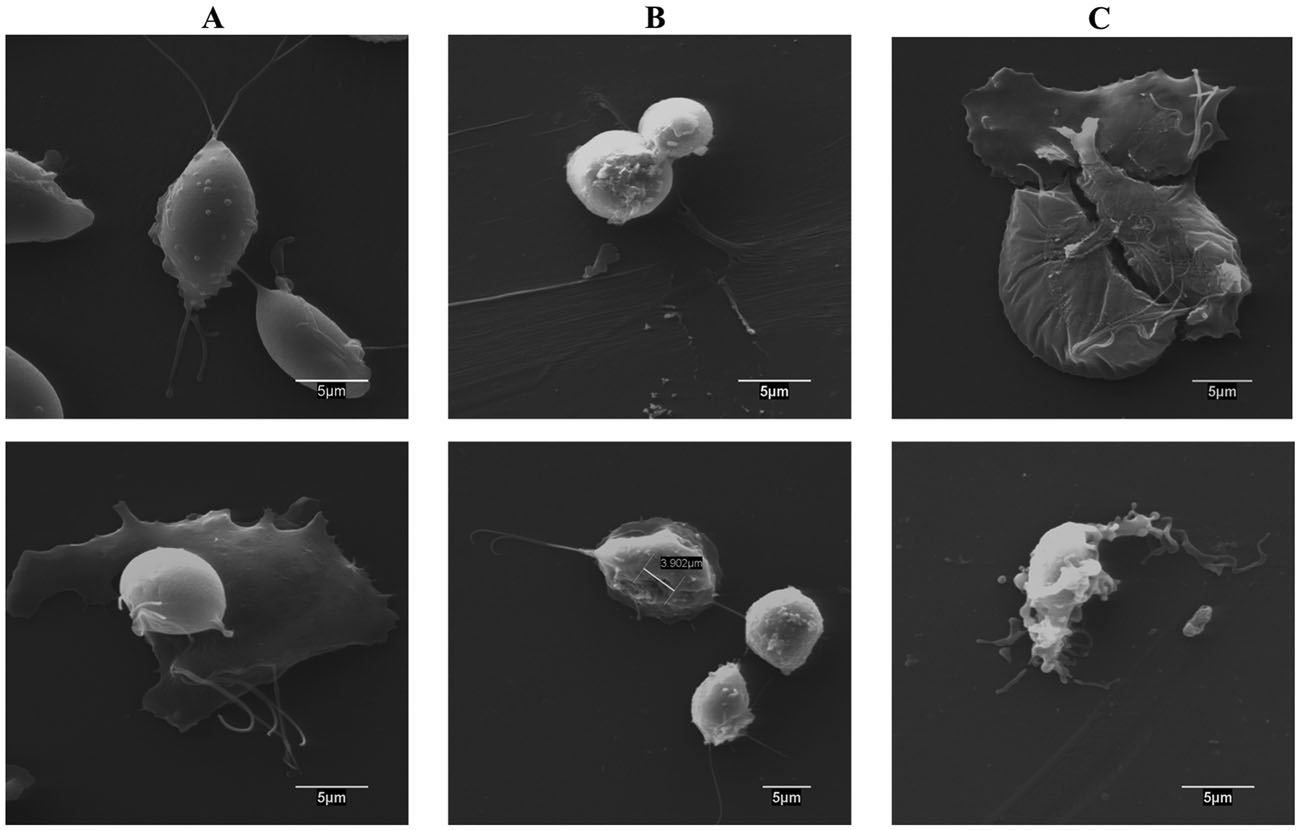
Scanning electron microscopy images of *T. vaginalis* cultures. A. Control. B. Treatment with compound **51**. C. Treatment with compound **63**.

With the aim of studying any structural alteration in the parasite triggered by these thiosemicarbazones, scanning electronic microscopy (SEM) was used to observe surface disturbances upon 24 h treatment at 15 µM concentration (Figure 4).

Control experiment (Figure 4(A)) shows trophozoites with no alterations in the plasmatic membrane, with pear-shaped or even amoeboid cells. Interestingly, treatment with thiosemicarbazone **51** induced a clear disturbance in the trophozoite surface (Figure 4(B)), showing parasites with a more rounded shape and even pseudocyst forms; some trophozoites were found to exhibit invaginations and pores in the surface; structural abnormalities have therefore an apoptotic aspect. It has been reported that *T. vaginalis* adopts this rounded shape, with internalised flagella in endocytic vesicles upon stress situation or lack of nutrients^66^. Regarding derivative **63**, it induced amoeboid cells in most of the culture with smooth disturbances in the cytoplasmatic surface and showing a wrinkled shape in many trophozoites, as depicted in Figure 4(C).

Therefore, these MoA experiments show that both thiosemicarbazones provoke a trichomonacidal effect in a hydrogenosome-independent mechanism inducing the trophozoites death with a clear disruption of the parasite surface.

## 4. Conclusions

In the present work we have accomplished the preparation of an ample number of thio(seleno)semicarbazones as potential antiparasitic agents against *T. cruzi* (responsible for Chagas disease) and *T. vaginalis* (responsible for trichomoniasis) with a mode of action different to that of classical nitroheterocyclic compounds, the current only available drugs for treating both parasitic infections. We have carried out an extensive analysis of SARs upon modification of the stereoelectronic effects of the aromatic substituents, together with the nature of the chalcogen atom (S *vs.* Se). Thiosemicarbazide **31**, bearing a naphthyl residue on N-4, exhibited the best trypanocidal activity at the epimastigote stage of two different parasitic strains, with similar profile to that of the reference drug benznidazole.

Moreover, halogenated thiosemicarbazones **49**, **51** and naphthyl-containing **63** exhibited a remarkable trichomonacidal profile, with activities within the low micromolar range, and excellent selectivities. Their selenium isosters afforded a slightly enhancement of the activity.

Analysis of the hydrogenosome membrane potential, and structural changes through scanning electronic microscopy (**51**, **63**) afforded valuable information concerning their mechanisms of action.

## Supplementary Material

Supplemental Material
