# Supplementary material for: Thio- and selenosemicarbazones as antiprotozoal agents against Trypanosoma cruzi and Trichomonas vaginalis
Source: J Enzyme Inhib Med Chem. 2022 Feb 23;37(1):781–91. doi: 10.1080/14756366.2022.2041629 (PMC8881069; doi:10.1080/14756366.2022.2041629)
Supplement: Supplemental Material [file IENZ_A_2041629_SM1301.pdf]

Thio- and selenosemicarbazones as antiprotozoal agents against *Trypanosoma cruzi* and *Trichomonas vaginalis*

Alexandra Ibáñez-Escribano,[a,#] Cristina Fonseca-Berzal,[a,#] Mónica Martínez-Montiel,[b,#] Manuel Álvarez-Márquez,[c] María Gómez-Núñez,[d] Manuel Lacueva-Arnedo,[a] Teresa Espinosa-Buitrago,[a] Tania Martín-Pérez,[e,f] José Antonio Escario,[a] Penélope Merino-Montiel,[b,*] Sara Montiel-Smith,[b] Alicia Gómez-Barrio,[a,*] Óscar López,[c,*] José G. Fernández-Bolaños[c]

[a]*Unidad de Parasitología, Departamento de Microbiología y Parasitología, Facultad de Farmacia, Pza. Ramón y Cajal s/n, 28040 Madrid, Spain e-mail: agbarrio@ucm.es*
[b]*Facultad de Ciencias Químicas, Ciudad Universitaria, Benemérita Universidad Autónoma de Puebla, 72570, Puebla, PUE, México. e-mail: penelope.merino@correo.buap.mx*
[c]*Departamento de Química Orgánica, Facultad de Química, Universidad de Sevilla, Apartado 1203, E-41071 Sevilla, Spain. e-mail: osc-lopez@us.es*
[d]*Escuela Politécnica Superior, Universidad de Sevilla, Virgen de África 7, E-41011 Seville, Spain*
[e]*Departamento de Biomedicina y Biotecnología, Facultad de Farmacia, Universidad de Alcalá, Alcalá de Henares, 28805 Madrid, Spain.*
[f]*Institute of Specific Prophylaxis and Tropical Medicine, Medical University of Vienna, Kinderspitalgasse 15, 1090 Vienna, Austria.*

[#]These authors contributed equally to the manuscript

Materials and methods……………………………………………………………………S2-S14

Biology……………………………………………………………………………S2-S6

Chemistry…………………………………………………………………………..S6-S14

^{1}H- and ^{13}C-NMR spectra …………………..…………………………………………...S15–S35

References……………………………………………...…………………………………S36-S37

Materials and methods

Biology

Compounds. Stock solutions of the synthesized compounds, the reference antichagasic drug BZ –kindly provided by LAFEPE, Laboratório Farmacêutico do Estado de Pernambuco, Brazil– and the reference trichomonacidal drug MTZ (Sigma-Aldrich Chemical Co., St. Louis, MO) were prepared in dimethyl sulfoxide (DMSO) and extemporaneously diluted in culture media in a final concentration of the solvent that did not exceed 0.2% v/v, which has no toxic effect over both parasites and mammalian cells [1-,4].

Study of potential trypanocidal activity. Trypanosoma cruzi cultures. Biological assays on *T. cruzi* were carried out against two different strains: CL-B5 *lacZ* [5] and Y [6], which are classified as DTUs TcVI and TcII, respectively [7]. Axenic cultures of *T. cruzi* epimastigotes were continuously maintained at 28 °C in log-phase, by weekly passages in Liver Infusion Tryptose (LIT) medium supplemented with 10% of heat-inactivated foetal bovine serum (FBS) and antibiotics (ie., 100 IU/mL penicillin and 100 ug/mL streptomycin). Tissue culture-derived trypomastigotes (TCT) of CL-B5 *lacZ* strain were obtained by infecting L929 cells maintained in Minimal Essential Medium (MEM) without phenol red, supplemented with 10% heat-inactivated FBS, 100 IU/mL penicillin and 100 µg/mL streptomycin, with epimastigotes at stationary phase (ie., 14-day-old cultures) for 24 h at 33 °C and 5% CO_2. Infected cultures were washed with phosphate-buffered saline (PBS) to remove non-internalized parasites and incubated with fresh MEM for 7 days at the aforementioned conditions of temperature and humidity. At this time, TCT were harvested in the supernatant of L929 infected cultures cultures and then, used for the obtention of intracellular amastigotes in the corresponding susceptibility assays [1,2,4].

Mammalian cell cultures. Cultures of L929 fibroblasts and J774 macrophages were grown at 37 °C with 5% CO_2 and respectively sustained in MEM and Roswell Park Memorial Institute (RPMI) medium without phenol red, both supplemented with 10% heat-inactivated FBS and antibiotics (ie., 100 IU/mL penicillin and 100 µg/mL streptomycin). Weekly sub-passages were carried out by detaching cells with a solution of 0.03% EDTA and 0.05% trypsin in PBS [4,8].

CL-B5 lacZ epimastigote susceptibility assays. Trypanocidal activity of the compounds was firstly evaluated over this DTU TcVI *lacZ* transfected strain in presence of the chromogenic substrate chlorophenol red β-D-galactopyranoside (CPRG) [4,9]. Cultures of log-phase epimastigotes in LIT medium were seeded in 96-well microplates (200 µL/well) at a density of 2.5×10^5 parasites/mL and incubated within serial solutions of compounds for 72 h at 28 °C. Then, 50 µL of CPRG solution in 0.9% Triton X-100 (final concentration 200 µM, pH 7.4) was added per well and after 3 h of incubation at 37 °C, absorbance read at 595 nm (ELx808 ELISA reader, Biotek Instruments Inc). Percentages of epimastigote growth inhibition (%EGI) and concentration that inhibits 50% of epimastigote growth (IC_{50}) were calculated as previously reported [4].

Y strain epimastigote susceptibility assays. The most promising compounds of these series, were also tested against the moderately-drug resistant Y strain (DTU TcII) in presence of the redox indicator resazurin, following the methodology standardized by Rolón *et al.* [10]. Accordingly, 3×10^6 log-phase epimastigotes/mL were distributed in 96-well microplates by seeding 200 µL/well. Parasites were treated for 48 h at 28 °C with serial solutions of compounds and then, 20 µL of resazurin solution in PBS (3 mM, pH 7.0) added per well. After 5 h of incubation at 28 °C, fluorescence intensity was read at 535 (excitation) and 590 (emission) nm in a Infinite 200 multifunctional microplate reader (Tecan) and %EGI and IC_{50} calculated [1,2].

Unspecific cytotoxicity assays on L929 fibroblasts. The cytotoxic profile of compounds was simultaneously explored over cultures of L929 cells. Accordingly, 100 µL/well of a suspension in MEM containing 10×10^4 cells were seeded in 96-well microplates and maintained for 3 h at 37 °C with 5% CO_2. After cell attachment, culture medium was replaced by 200 µL of compounds serially diluted in fresh MEM and plates incubated for 72 h at the aforementioned conditions of temperature and humidity. Then, 20 µL of a resazurin solution in PBS (2 mM, pH 7.0) was added and plates incubated for 3 h (37 °C, 5% CO_2). Fluorescence intensity was read at 535 (excitation) and 590 (emission) nm in a Infinite 200 multifunctional microplate reader (Tecan). Percentages of cytotoxicity ($\%C_{L929}$) and concentration that produces 50% of cell death (CC_{50}) were calculated as reported by Fonseca-Berzal *et al.* [4].

Unspecific cytotoxicity assays on J774 macrophages. For the most promising compounds, the potential toxic effects were also explored on J774 macrophages [8]. Briefly, a suspension of 50 × 10^4 cells in RPMI medium was distributed in 96-well microplates by adding 100 µL/well. After 3 h of incubation at 37 °C with 5% CO_2, culture medium was discarded and 200 µL of compounds serially diluted in RPMI added. Cells were treated for 48 h (37 °C, 5% CO_2) and then, 20 µL of a resazurin solution in PBS (1 mM, pH 7.0) added to each well. Fluorescence intensity was read at 535 (excitation) and 590 (emission) nm in a Infinite 200 multifunctional microplate reader (Tecan). Finally, percentages of cytotoxicity (%C_{J774}), CC_{50} and SI on Y epimastigotes were calculated [4,11].

CL-B5 lacZ intracellular amastigote susceptibility assays. Those compounds with trypanocidal profile over epimastigotes similar to that of BZ, were moved to a more specific assay against intracellular amastigotes. These assays were carried out by applying the CPRG method reported by Fonseca-Berzal *et al.* [4]. Accordingly, 10,000 L929 cells were seeded in 48-well plates and infected with CL-B5 *lacZ* tissue culture-derived trypomastigotes at 1:6 ratio (cell:parasite). After 24 h of incubation at 33 °C with 5% CO_2, non-penetrated parasites were rinsed with PBS and then, infected cultures treated with compounds diluted in fresh MEM for 7 days in similar conditions of temperature and humidity. Finally, 50 µL of CPRG solution in 3% Triton X-100 (final concentration 400 µM, pH 7.4) was added and after 3 h of incubation at 37 °C, absorbance read at 595 nm in a Infinite 200 multifunctional microplate reader (Tecan). Percentages of amastigote growth inhibition (%AGI), IC_{50} and SI on CL *lacZ* amastigotes were also calculated.

For each experiment, the assays were run at the same conditions three times separately (n=3) and therefore, both activity and cytotoxicity results are expressed as the mean value of the respective IC_{50} or CC_{50} ± standard deviation (SD).

Study of trichomonacidal activity. T. vaginalis culture. Trichomonas vaginalis isolate JH31#A4 from the American Type Culture Collection (ATCC, Ref. No. 30236) was used for the *in vitro* trichomonacidal screening assays. The parasites were cultured in trypticase-yeast extract-maltose (TYM) medium supplemented with 10% heat-inactivated FBS and antibiotic solution (100 IU/mL penicillin and 100 µg/mL streptomycin) at 37 °C in a humidified atmosphere with 5% CO_2 [1].

Mammalian cell cultures. Different mammalian cell lines were used to evaluate the specific antiparasitic activity of the most interesting compounds. African green monkey kidney epithelial cells (Vero CCL-91, ATCC) were grown in RPMI-1640 medium supplemented with 10% FBS and antibiotic solution. The cells were cultured in sterile plastic flasks at 37 °C in a 5% CO_2 incubator and passages were executed when reaching 80-90% confluence checked at the inverted microscope [12].

Trichomonacidal in vitro assays. Parasites in a logarithmic growth phase, after 5 h of incubation from an initial culture of 1 x 10^5 trophozoites/mL, were incubated again with the different compounds at two-fold dilutions (from 300 to 9.37 µM) for 24 h. Metronidazole at 24 µM was used as positive control and DMSO solvent at 0.2% v/v was also added in growth controls. Then, each tube was seeded in a 96 well flat-bottom microplate, posteriorly centrifuged at 300g for 5 min and subsequently, TYM medium was replaced with 200 µL of sterile PBS 1X and 20 µL of resazurin, previously prepared at a stock concentration of 3 mM in PBS. After 1 h of incubation with the redox dye, plates were read in a Infinite 200 multifunctional microplate reader (Tecan) at $\lambda_{excitation\text{-}emission}$ of 535-590 nm, respectively as indicates the method described by Ibañez Escribano *et al.* [1].

Unspecific cytotoxicity assays in mammalian cells. The most active compounds against *T. vaginalis* were posteriorly screened in Vero CCL-81 cells according to the sequential protocol used in the laboratory [1,12-14]. Briefly, Vero cells were seeded (5 × 10^4 cells/well) and plates were incubated for 6 h at the same conditions as previously described. After 6 h, the compounds were added at the same concentrations evaluated in the *T. vaginalis* assays. The cytotoxicity effect over cells was evaluated after 24 h of incubation with the compounds using the resazurin protocol at 1 mM stock solution and after 3 h with the dye. The 96 well-plates were read at $\lambda_{excitation}$ 535 nm and $\lambda_{emission}$ 590 nm in a Infinite 200 multifunctional microplate reader (Tecan).

Statistical determinations. PROBIT analysis (SPSS v.22, IBM) was used for the determination of the 50% inhibitory concentration (IC_{50}) in *T. vaginalis* assays, the 50% cytotoxic concentration (CC_{50}) against Vero CCL-81 cells and the 95% confidence intervals. All the experiments were performed at least two times in triplicate. The growth

inhibition values used to calculate these parameters presented a standard deviation below 10%.

Mode of action (MoA) determinations

Hydrogenosomal Membrane Potential Assay. Loss of hydrogenosomal membrane integrity was evaluated with the JC-1 Mitochondrial Membrane potential Assay Kit (Cayman Chemical, USA) following the recommendations of the manufacturer. Briefly, 5×10^5 trophozoites JH31A#4 were seeded in 96-well microplates in a final volume of 100 µL and cultivated for 4 h with compounds **51** and **63** at a final concentration of 100 µM. Then, a volume of 10 µL of the cationic vital JC-1 dye (1:10 in TYM medium) was added. After 15 minutes of incubation, the plates were washed twice in the buffer solution provided in the kit. The presence of J-monomers was measured in a Infinite 200 multifunctional microplate reader (Tecan) at 535 nm and observed under fluorescence microscope. All the assays have been conducted in the presence of 50 µM of the uncoupler agent CCCP (*m*-chlorocarbonylcyanide phenylhydrazone) as a positive control [15]. Moreover, all the determinations were evaluated twice and at least in two independent experiments.

Scanning Electron Microscopy. Trophozoites were placed in a glass coverslip coated with 0.1 % poly L-Lysine solution during 1 h and were fixed in Milloning's solution containing 2% glutaraldehyde during 1 h. Then the samples were washed in Milloning's solution with 0.5% glucose and dehydrated in ethanol series and anhydrous acetone. Samples were critical-point dried using a Polaron CPD7501 critical-point drying system and sputter coated with 200 Å gold–palladium using a Polaron E5400. SEM observations were performed at 5–15 kV on a Zeiss DSM950 SEM.

Chemistry

*General procedure for the preparation of isothiocyanates **11–20**.*

To a solution of the corresponding aniline **1–10** (3.0 g) in a 1:1 CH_2Cl_2–H_2O mixture (24 mL) were added triphosgene (1.5 equiv.) and $CaCO_3$ (2.0 equiv.); the corresponding mixture was vigorously stirred for 2 h, at rt. After that, it was filtrated, and the organic layer was separated, dried over Na_2SO_4, filtrated and concentrated under vacuum.

Isothiocyanates **11**–**20** were directly used for the next step, without any further purifications.

*General procedure for the preparation of thiosemicarbazides **22–30**.*

To a solution of isothiocyanates **11**–**20** (1.0 equiv.) in CH_2Cl_2 was dropwise added hydrazine (1.2 equiv.). After the addition, the solution was kept at rt for 3 h, yielding precipitation of the target compounds. The solid was filtrated and washed with CH_2Cl_2 to give derivatives **22**–**30**.

*General procedure for the preparation of thiosemicarbazones **36–65**.*

A solution of thiosemicarbazides **21–30** (500 mg) and the corresponding aromatic aldehyde (1.0 equiv.) in EtOH (15 mL) was refluxed overnight. After cooling to rt, the products precipitated and were washed with cold EtOH. 3,4-Dihydroxyphenyl derivatives were concentrated to dryness and precipitated with Et_2O.

*1-(4'-Hydroxybenzylidene)-4-(*p*-methylphenyl)-3-thiosemicarbazone (**39**).* 4-(*p*-Methylphenyl)-3-thiosemicarbazide **22** (500 mg, 3.03 mmol) and *p*-hydroxybenzaldehyde **32** (370 mg, 3.03 mmol) were used. Yield: 260 mg (33%). Mp: 179–183 ºC. ^{1}H-NMR (300 MHz, DMSO-d_6) δ 11.58 (s, 1H, NH), 9.89 (s, 1H, Ar-NH), 9.88 (s, 1H, OH) 8.05 (s, 1H, N=CH), 7.70 (m, 2H, H-2', H-6'), 7.43 (m, 2H, Ar-H*o*), 7.14 (m, 2H, Ar-H*m*), 6.79 (m, 2H, H-3', H-5'), 2.30 (s, 3H, CH_3) ppm; ^{13}C-NMR (125.7 MHz, DMSO-d_6) δ: 175.6 (C=S), 159.6 (C-4'), 143.3 (C=N), 136.7 (C-4), 134.4 (C-1), 129.6 (C-2', C-6'), 128.6 (C-3, C-5), 125.7 (C-1'), 125.1 (C-2, C-6), 115.7 (C-3', C-5'), 20.7 (CH_3) ppm; HRESI–MS *m/z* calcd. for $C_{15}H_{16}N_3OS$ ($[M + H]^+$): 286.1009, found: 286.1012.

*1-(4'-Methoxybenzylidene)-4-(*p*-methylphenyl)-3-thiosemicarbazone (**40**).* 4-(*p*-Methylphenyl)-3-thiosemicarbazide **22** (500 mg, 3.03 mmol) and *p*-methoxybenzaldehyde **33** (0.37 mL, 3.03 mmol) were used. Yield: 720 mg (88%). Mp: 187–190 ºC; ^{1}H-NMR (300 MHz, DMSO-d_6) δ 11.66 (s, 1H, NH), 9.96 (s, 1H, Ar-NH), 8.11 (s, 1H, N=CH), 7.83 (m, 2H, H-2', H-6'), 7.43 (m, 2H, Ar-H*o*), 7.15 (m, 2H, Ar-H*m*), 6.97 (m, 2H, H-3', H-5'), 3.79 (s, 3H, OCH_3), 2.30 (s, 3H, CH^3) ppm; ^{13}C-NMR (125.7 MHz, DMSO-d_6) δ 175.8

(C=S), 161.0 (C-4’), 142.8 (C=N), 136.8 (C-4), 134.5 (C-1), 129.4 (C-2’, C-6’), 128.6 (C-3, C-5), 126.7 (C-1’), 125.9 (C-2, C-6), 114.3 (C-3’, C-5’), 55.4 (OCH_3), 20.7 (CH_3) ppm; HRESI–MS *m/z* calcd. for $C_{16}H_{18}N_3OS$ ($[M + H]^+$): 300.1165, found: 300.1167.

*1-(3’,4’-Dihydroxybenzylidene)-4-(*p*-methylphenyl)-3-thiosemicarbazone* ***(41)***. 4-(*p*-Methylphenyl)-3-thiosemicarbazide **22** (500 mg, 3.03 mmol) and 3,4-dihydroxybenzaldehyde **34** (418 mg, 3.03 mmol) were used. Yield: 571 mg (54%). Mp: 198–200 ºC. ^{1}H-NMR (300 MHz, DMSO-d_6) δ 11.55 (s, 1H, NH), 9.85 (s, 1H, Ar-NH), 9.51 (brs, 1H, OH), 9.09 (brs, 1H, OH) 8.00 (s, 1H, N=CH), 7.44 (m, 2H, Ar-H*o*), 7.32 (d, 1H, $J_{2',6'}$ =1.9 Hz, H-2’), 7.14 (m, 2H, Ar-H*m*), 7.10 (dd, 1H, $J_{2',6'}$ =1.9 Hz, $J_{5',6'}$ =8.2 Hz, H-6’), 6.79 (d, 1H, H-5’), 2.30 (s, 3H, CH_3) ppm. ^{13}C-NMR (125.7 MHz, DMSO-d_6) δ 175.6 (C=S), 148.1 (C-4’), 145.7 (C-3’), 143.7 (C=N), 136.7 (C-4), 134.4 (C-1), 128.7 (C-3, C-5), 125.7 (C-1’), 125.6 (C-2, C-6), 120.8 (C-6’), 115.7 (C-5’), 114.2 (C-2’), 20.8 (CH_3) ppm; HRESI-MS *m/z* calcd. for $C_{15}H_{16}N_3O_2S$ ($[M + H]^+$): 302.0958, found: 302.0960.

*1-(4’-Hydroxybenzylidene)-4-(*p*-methoxylphenyl)-3-thiosemicarbazone* ***(42)***. 4-(*p*-Methoxyphenyl)-3-thiosemicarbazide **23** (500 mg, 2.53 mmol) and *p*-hydroxybenzaldehyde **32** (310 mg, 2.53 mmol) were used. Yield: 428 mg (56%). Mp: 140–145 ºC. ^{1}H-NMR (300 MHz, DMSO-d_6) δ 11.55 (s, 1H, NH), 9.93 (s, 1H, Ar-NH), 9.86 (s, 1H, OH) 8.05 (s, 1H, N=CH), 7.71 (m, 2H, H-2’, H-6’), 7.38 (m, 2H, Ar-H*o*), 6.91 (m, 2H, Ar-H*m*), 6.80 (m, 2H, H-3’, H-5’), 3.76 (s, 3H, OMe) ppm; ^{13}C-NMR (125.7 MHz, DMSO-d_6) δ 175.9 (C=S), 159.6 (C-4’), 143.3 (C=N), 136.7 (C-4), 134.4 (C-1), 129.6 (C-2’, C-6’), 128.6 (C-3, C-5), 125.7 (C-1’), 125.1 (C-2, C-6), 115.7 (C-3’, C-5’), 20.7 (CH3) ppm; HRESI-MS *m/z* calcd. for $C_{15}H_{16}N_3O_2S$ ($[M + H]^+$): 302.0958, found: 302.0961.

*4-(4’-Fluorophenyl)-1-(*p*-methoxybenzylidene)-3-thiosemicarbazone* ***(44)***. 4-(4’-Fluorophenyl)-3-thiosemicarbazide **24** (500 mg, 3.26 mmol) and *p*-methoxybenzaldehyde **33** (0.40 mL, 3.26 mmol) were used. Yield: 770.9 mg (78%). Mp: 187–192 ºC. ^{1}H-NMR (300 MHz, DMSO-d_6) δ 11.72 (s, 1H, NH), 10.03 (s, 1H, Ar-NH), 8.09 (s, 1H, N=CH), 7.83 (m, 2H, H-2’’, H-6’’), 7.69 (app dd, 2H, $^4J_{H,F}$= 5.2 Hz, H-2’, H-6’), 7.18 (app t, 2H, $^3J_{H,F}$= 8.9 Hz, H-3’, H-5’), 6.97 (m, 2H, H-3’’, H-5’’), 3.79 (s, 3H, OMe) ppm; ^{13}C-NMR (125.7 MHz, DMSO-d_6) δ: 176.1 (C=S), 161.2 (C-4’’), 159.6 (d, $^1J_{C,F}$= 241.1 Hz, C-4’),

143.1 (C=N), 135.6 (d, $^4J_{C,F}$= 2.8 Hz, C-1'), 130.0 (C-2'', C-6''), 128.1 (d, $^3J_{C,F}$= 8.4 Hz, C-2', C-6'), 126.7 (C-1''), 114.6 (d, $^2J_{C,F}$= 23.0 Hz, C-3', C-5'), 114.3 (C-3'', C-5''), 55.4 (OMe) ppm; HRESI-MS *m/z* calcd. for $C_{15}H_{15}FN_3OS$ ($[M + H]^+$): 304.0914, found: 304.0917.

4-(4'-Fluorophenyl)-1-(3'',4''-dihydroxybenzylidene)-3-thiosemicarbazone ***(45)***. 4-(4'-Fluorophenyl)-3-thiosemicarbazide **24** (500 mg, 3.26 mmol) and 3,4-dihydroxybenzaldehyde **34** (450 mg, 3.26 mmol). Yield: 27.6 mg (3%). ^{1}H-NMR (300 MHz, DMSO-d_6) δ: 11.63 (s, 1H, NH), 9.95 (s, 1H, Ar-NH), 9.55 (s, 1H, OH), 9.01 (s, H, OH), 8.00 (s, 1H, N=CH), 7.57–7.52 (m, 2H, Ar-H*o*), 7.31 (d, 1H, $J_{2'',6''}$= 2.0 Hz, H-2''), 7.17 (app t, 2H, $^3J_{H,F}$= 8.9 Hz, H-3', H-5'), 7.09 (dd, 1H, $J_{2'',6''}$= 2.0 Hz, $J_{5'',6''}$= 8.4 Hz, H-6''), 6.76 (d, 1H, $J_{5'',6''}$= 8.4 Hz, H-5'') ppm; ^{13}C-NMR (125.7 MHz, DMSO-d_6) δ 175.7 (C=S), 159.1 (d, $^1J_{C,F}$= 241.9 Hz, C-4'), 148.0, 145.9, 143.9 (C=N, C-4'', C-3''), 135.6 (d, $^4J_{C,F}$= 2.7 Hz, C-1'), 127.8 (d, $^3J_{C,F}$= 8.5 Hz, C-2', C-6'), 125.4 (C-1''), 120.7 (C-6'), 115.5 (C-5'), 114.7 (d, $^2J_{C,F}$= 22.6 Hz, C-3', C-5'), 114.2 (C-2'') ppm.

*4-(*p*-Chlorophenyl)-1-(4'-methoxybenzylidene)-3-thiosemicarbazone* ***(46)***. 4-(*p*-Chlorophenyl)-3-thiosemicarbazide **25** (500 mg, 2.48 mmol) and *p*-methoxybenzaldehyde **33** (0.30 mL, 2.48 mmol) were used. Yield: 0.54 g (68%). Mp: 177–181 ºC. ^{1}H-NMR (300 MHz, DMSO-d_6) δ 11.80 (s, 1H, NH), 10.05 (s, 1H, Ar-NH), 8.12 (s, 1H, N=CH), 7.84 (m, 2H, H-2', H-6'), 7.63 (m, 2H, Ar-H*o*), 7.41 (m, 2H, Ar-H*m*), 6.98 (m, 2H, H-3', H-5'), 3.80 (s, 3H, CH_3) ppm; ^{13}C-NMR (125.7 MHz, DMSO-d_6) δ 175.6 (C=S), 160.9 (C-4'), 143.2 (C=N), 138.1 (C-1), 129.3 (C-4), 129.1 (C-2, C-6), 127.9 (C-2', C-6'), 127.3 (C-3, 5), 126.4 (C-1'), 114.1 (C-3', C-5'), 55.3 (OMe) ppm; HRESI-MS *m/z* calcd. for $C_{15}H_{15}ClN_3OS$ ($[M + H]^+$): 320.0619, found: 320.0623.

*4-(*p*-Bromophenyl)-1-(3',4'-dihydroxybenzylidene)-3-thiosemicarbazone* ***(49)***. 4-(*p*-Bromophenyl)-3-thiosemicarbazide **26** (500 mg, 2.03 mmol) and 3,4-dihydroxybenzaldehyde **34** (280 mg, 2.30 mmol) were used. Yield: 480 mg (65%). Mp: 189–193 ºC. ^{1}H-NMR (300 MHz, DMSO-d_6) δ 11.66 (s, 1H, NH), 9.95 (s, 1H, Ar-NH), 9.53 (brs, 1H, OH), 8.99 (brs, 1H, OH) 7.98 (s, 1H, N=CH), 7.53 (m, 4H, Ar-H*o*, Ar-H*m*), 7.30 (d, 1H, $J_{2',6'}$ =2.0 Hz, H-2'), 7.07 (dd, 1H, $J_{2',6'}$ =2.0 Hz, $J_{5',6'}$ = 8.2 Hz, H-6'), 6.75 (d, 1H, $J_{5',6'}$ = 8.2 Hz, H-5') ppm; ^{13}C-NMR (125.7 MHz, DMSO-d_6) δ 175.3 (C=S), 148.2 (C-

4’), 145.7 (C-3’), 144.3 (C=N), 138.8 (C-1), 130.9 (C-4), 127.5 (C-2, C-6), 125.4 (C-1’), 120.7 (C-6’),117.2 (C-3, C-5), 115.4 (C-5’), 114.3 (C-3’) ppm; HRESI-MS *m/z* calcd. for $C_{14}H_{13}BrN_3O_2S$ ($[M + H]^+$): 365.9906, found: 365.9903.

*4-(*p*-Iodophenyl)-1-(4’-methoxybenzylidene)-3-thiosemicarbazone (**51**).* 4-(*p*-Iodophenyl)-3-thiosemicarbazide **27** (500 mg, 1.71 mmol) and *p*-methoxybenzaldehyde **33** (0.21 mL, 1.71 mmol) were used. Yield: 0.65 g (92%). Mp: 183–186 ºC. ^{1}H-NMR (300 MHz, DMSO-d_6) δ 11.77 (s, 1H, NH), 10.02 (s, 1H, Ar-NH), 8.09 (s, 1H, N=CH), 7.82 (m, 2H, H-2’, H-6’), 7.68 (m, 2H, Ar-H*o*), 7.43 (m, 2H, Ar-H*m*), 6.96 (m, 2H, H-3’, H-5’), 3.78 (s, 3H, OMe) ppm; ^{13}C-NMR (125.7 MHz, DMSO-d_6) δ 175.3 (C=S), 160.9 (C-4’), 143.1 (C=N), 139.0 (C-1), 136.6 (C-2, C-6), 129.3 (C-2’, C-6’), 127.7 (C-3, C-5), 126.6 (C-1’), 114.1 (C-3’, C-5’), 89.6 (C-4), 55.2 (OMe); HRESI-MS *m/z* calcd. for $C_{15}H_{15}IN_3OS$ ($[M + H]^+$): 411.9975, found: 411.9974.

*1-(3’,4’-Dihydroxybenzylidene)-4-(*p*-iodophenyl)-3-thiosemicarbazone (**52**).* 4-(*p*-Iodophenyl)-3-thiosemicarbazide **27** (500 mg, 1.71 mmol) and 3,4-dihydroxybenzaldehyde **34** (240 mg, 1.71 mmol) were used. Yield: 431 mg (61%). Mp: 178–182 ºC (dec.). ^{1}H-NMR (300 MHz, DMSO-d_6) δ: 11.64 (s, 1H, NH), 9.95 (s, 1H, Ar-NH), 8.01 (s, 1H, N=CH), 7.69 (m, 2H, Ar-H*m*), 7.47 (m, 2H, Ar-H*o*), 7.32 (d, 1H, $J_{2',6'}$= 1.7 Hz, H-2’), 7.10 (dd, $J_{2',6'}$= 1.7 Hz, 1H, $J_{5',6'}$= 8.7 Hz, H-6’), 6.78 (d, 1H, $J_{5',6'}$= 8.7 Hz, H-5’) ppm; ^{13}C-NMR (125.7 MHz, DMSO-*d6*) δ 175.0 (C=S), 148.0, 145.5, 144.5 (C-3’, C-4’, C=N), 139.0 (C-3’’, C-5’’), 136.6 (C-1’), 127.3 (C-1’’), 125.2 (C-2’’, C-6’’), 120.6 (C-6’’), 115.4 (C-5’’), 114.1(C-2’’), 89.4 (C-4’) ppm; HRESI-MS *m/z* calcd. for $C_{15}H_{13}IN_3OS$ ($[M + H]^+$): 413.9768, found: 413.9764.

*1-(4’-Methoxybenzylidene)-4-(2’’-nitrophenyl)-3-thiosemicarbazone (**54**).* 4-(2’’-Nitrophenyl)-3-thiosemicarbazide **28** (500 mg, 2.35 mmol) and *p*-methoxybenzaldehyde (0.28 mL; 2.35 mmol) were used. Yield: 761 mg (98%). Mp: 180–185 ºC (dec.). ^{1}H-NMR (300 MHz, DMSO-d_6) δ: 12.17 (s, 1H, NH), 11.11 (s, 1H, Ar-NH), 8.49 (brd, 1H, $J_{5'',6''}$= 8.3 Hz, H-6’’), 8.18 (s, 1H, N=CH), 8.15 (dd, 1H, $J_{3'',4''}$=8.2 Hz, $J_{3'',5''}$= 1.3 Hz, H-3’’), 7.82 (m, 2H, H-2’, H-6’), 7.75 (td, 1H, $J_{3'',5''}$= 1.3 Hz, $J_{5'',6''}$= $J_{4'',5''}$= 8.3 Hz, H-5’’), 7.45 (td, 1H, $J_{2'',4''}$= 1.2 Hz, $J_{3'',4''}$= $J_{4'',5''}$= 8.2 Hz, H-4’’), 7.04 (m, 2H, H-3’, H-5’), 3.83 (s, 3H, OMe) ppm; ^{13}C-NMR (125.7 MHz, DMSO-d_6) δ 175.1 (C=S), 161.2 (C-4’), 143.9 (C=N),

141.8 (C-2’’), 134.0, 133.9 (C-1’’, C-2’, C-6’), 129.2 (C-5’’), 127.0 (C-1’), 126.2, 125.5 (C-3’’, C-4’’), 125.1 (C-6’’), 114.4 (C-3’, C-5’), 55.4 (OMe) ppm; HRESI-MS *m/z* calcd. for $C_{15}H_{15}N_4O_3S$ ($[M + H]^+$): 331.0859, found: 331.0859.

1-(3’,4’-Dihydroxybenzylidene)-4-(2’’-nitrophenyl)-3-thiosemicarbazone ***(55)***. 4-(2’’-Nitrophenyl)-3-thiosemicarbazide **28** (500 mg, 2.35 mmol) and and 3,4-dihydroxybenzaldehyde **34** (320 mg, 2.35 mmol) were used. Yield: 222 mg (28%). Mp: 181–184 ºC (dec.). ^{1}H-NMR (300 MHz, DMSO-d_6) δ: 12.07 (s, 1H, NH), 11.00 (s, 1H, Ar-NH), 8.70 (s, 1H, H-6), 9.63 (s, 1H, OH), 9.08 (s, 1H, OH), 8.48 (dd, 1H, $J_{4'',6''}$= 1.2 Hz, $J_{5'',6''}$= 8.3 Hz, H-6’’), 8.13 (dd, 1H, $J_{3'',5''}$= 1.5 Hz, $J_{3'',4''}$= 8.3 Hz, H-3’’), 8.05 (s, 1H, N=CH), 7.76 (td, 1H, $J_{3'',5''}$= 1.5 Hz, $J_{4'',5''}$= $J_{5'',6''}$= 8.3 Hz, H-5’’), 7.41 (td, $J_{4'',6''}$= 1.2 Hz, $J_{3'',4''}$= $J_{4'',5''}$= 8.3 Hz, 1H, H-4’’), 7.29 (d, 1H, $J_{2',6'}$= 2.0 Hz, H-2’), 7.11 (dd, 1H, $J_{2',6'}$= 2.0 Hz, $J_{5',6'}$= 8.2 Hz, H-6’), 6.81 (d, 1H, $J_{5',6'}$= 8.2 Hz, H-5’) ppm; ^{13}C-NMR (125.7 MHz, DMSO-d_6) δ: 174.8 (C=S), 148.4 (C.4’), 145.7 (C-2’’), 145.0 (C=N), 141.7 (C-3’), 133.9 (C-1’’), 127.1 (C-1’), 125.4 (C-5’’), 125.3 (C-3’’, C-4’’), 120.7, 119.1 (C-6’, C-6’’), 115.5 (C-5’), 114.6 (C-2’) ppm; HRESI-MS *m/z* calcd. for $C_{14}H_{13}N_4O_4S$ ($[M + H]^+$): 333.0652, found: 333.0653.

*1-(4’-Hydroxybenzylidene)-4-(*m*-nitrophenyl)-3-thiosemicarbazone* ***(56)***. 4-(3’-Nitrophenyl)-3-thiosemicarbazide **29** (500 mg, 2.35 mmol) and *p*-hydroxybenzaldehyde **32** (290 mg, 2.35 mmol) were used. Yield: 470 mg (63%). Mp: 152–157 ºC. ^{1}H-NMR (300 MHz, DMSO-d_6) δ: 11.90 (s, 1H, NH), 10.27 (s, 1H, Ar-NH), 9.97 (s, 1H, OH), 8.68 (m, 1H, H-2’’), 8.13 (m, 2H, H-4’’, H-6’’), 8.03 (s, 1H, N=CH), 7.77 (m, 2H, H-2’, H-6’), 7.61 (m, 1H, H-5’’), 6.85 (m, 2H, H-3’, H-5’) ppm; ^{13}C-NMR (125.7 MHz, DMSO-d_6) δ 175.4 (C=S), 159.6 (C-4’), 147.4 (C-3’’), 144.5 (C=N), 140.6 (C-1’’), 131.7 (C-6’’), 129.8 (C-2’, C-6’), 129.3 (C-5’’), 124.9 (C-1’), 119.6 (C-2’’, C-4’’), 115.7 (C-3’, C-5’) ppm; HRESI-MS *m/z* calcd. for $C_{14}H_{13}N_4O_3S$ ($[M + H]^+$): 317.0703, found: 317.0700.

*1-(4’-Methoxybenzylidene)-4-(*m*-nitrophenyl)-3-thiosemicarbazone* ***(57)***. *4-(3’-*Nitrophenyl)-3-thiosemicarbazide **29** (500 mg, 2.35 mmol) and *p*-methoxybenzaldehyde **33** (0.28 mL, 2.35 mmol) were used. Yield: 655 mg (84%). Mp: 178–181 ºC. ^{1}H-NMR (300 MHz, DMSO-d_6) δ 11.96 (s, 1H, NH), 10.33 (s, 1H, Ar-NH), 8.64 (brt, 1H, $J_{2'',6''}$=$J_{2'',4''}$= 2.0 Hz, H-2’’), 8.15 (s, 1H, N=CH), 8.11 (dd, 1H, $J_{2'',4''}$= 2.0 Hz, $J_{4'',5''}$= 8.5 Hz, H-4’’),

8.04 (dd, 1H, $J_{2'',6''}$= 2.0 Hz, $J_{5'',6''}$= 8.5 Hz, H-6''), 7.86 (m, 2H, H-2', H-6'), 7.64 (t, $J_{4'',5''}$= $J_{5'',6''}$= 8.5 Hz, 1H, H-5'') 7.00 (m, 2H, H-3', H-5'), 3.80 (s, 3H, OMe); ^{13}C-NMR (125.7 MHz, DMSO-d_6) δ 175.9 (C=S), 161.5 (C-4'), 147.7 (C-3'') 144.3 (C=N), 140.8 (C-1''), 132.0 (C-6''), 129.9 (C-2', C-6'), 129.6 (C-5''), 126.7 (C-1'), 120.0 (C-2''), 119.9 (C-4''), 114.6 (C-3', C-5'), 55.4 (OMe) ppm; HRESI-MS *m/z* calcd. for $C_{15}H_{15}N_4O_3S$ ([M + H]$^+$): 331.0859, found: 331.0860.

*1-(3',4'-Dihydroxybenzylidene)-4-(*m*-nitrophenyl)-3-thiosemicarbazone (**58**).* 4-(*m*-Nitrophenyl)-3-thiosemicarbazide **29** (500 mg, 2.35 mmol) and 3,4-dihydroxybenzaldehyde **34** (320 mg, 2.35 mmol) were used. Yield: 202 mg (26%). Mp: 170–175 ºC (dec.). ^{1}H-NMR (300 MHz, DMSO-d_6) δ 11.74 (s, 1H, NH), 10.19 (s, 1H, Ar-NH), 8.58 (t, 1H, $J_{2'',4''}$= $J_{2'',6''}$= 2.1 Hz, H-2''), 8.02–7.95 (m, 2H, H-4'', H-6''), 7.97 (s, 1H, N=CH), 7.60 (t, 1H, $J_{4'',5''}$= $J_{5'',6''}$= 8.4 Hz, H-5''), 7.30 (d, 1H, $J_{2',6'}$= 2.0 Hz, H-2'), 7.10 (dd, 1H, $J_{2',6'}$= 2.0 Hz, $J_{5',6'}$= 8.2 Hz, H-6'), 6.76 (d, 1H, $J_{5',6'}$= 8.2 Hz, H-5') ppm; ^{13}C-NMR (125.7 MHz, DMSO-d_6) δ 175.1 (C=S), 148.2, 147.2 (C-4', C-3''), 145.6 (C=N), 144.5 (C-3'), 140.4 (C-1''), 131.4, 129.1, 125.2 (C-1', C-5'', C-6''), 120.8 (C-6'), 119.3 (C-2'', C-4'') 115.4 (C-5'), 114.1 (C-2') ppm; HRESI-MS *m/z* calcd. for $C_{14}H_{13}N_4O_4S$ ([M + H]$^+$): 333.0652, found: 333.0657.

*1-(3',4'-Dihydroxybenzylidene)-4-(*p*-nitrophenyl)-3-thiosemicarbazone (**61**).* 4-(*p*-Nitrophenyl)-3-thiosemicarbazide (500 mg, 2.36 mmol) and 3,4-dihydroxyphenylbenzaldehyde (326 mg, 2.36 mmol) were used. Yield: 650 mg (83%). Mp: 199–201 ºC. ^{1}H-NMR (300 MHz, DMSO-d_6) δ: 11.94 (s, 1H, NH), 10.29 (s, 1H, Ar-NH), 9.62 (brs, 1H, OH), 9.10 (brs, 1H, OH), 8.21 (m, 2H, Ar-H*o*), 8.08 (s, 1H, N=CH), 8.04 (m, 2H, Ar-H*m*), 7.33 (d, 1H, $J_{2',6'}$= 1.2 Hz, H-2'), 7.11 (dd, 1H, $J_{2',6'}$= 1.2 Hz, $J_{5',6'}$ = 8.3 Hz, H-6') 6.75 (d, $J_{5',6'}$ = 8.3 Hz, 1H, H-5'). ^{13}C-NMR (125.7 MHz, DMSO-d_6) δ: 174.6 (C=S), 148.3 (C-4'), 145.6 (C=N), 145.5 (C-3'), 145.1 (C-1), 143.2 (C-4), 125.0 (C-1'), 123.8 (C-2, C-6), 123.7 (C-3, C-5), 121.0 (C-6'), 115.5 (C-5'), 114.2 (C-2') ppm; HRESI-MS *m/z* calcd. for $C_{14}H_{13}N_4O_4S$ ([M + H]$^+$): 333.0652, found: 333.0653.

*1-(4'-Nitrobenzylidene)-4-nitrophenyl-3-thiosemicarbazone (**62**).* 4-(*p*-Nitrophenyl)-3-thiosemicarbazide (500 mg, 2.36 mmol) and *p*-nitrobenzaldehyde (357.2 mg, 2.36 mmol). Yield: 786.8 mg (97%). Mp: 240–243 ºC. ^{1}H-NMR (300 MHz, DMSO-d_6) δ 12.38 (s, 1H,

NH), 10.57 (s, 1H, Ar-NH), 8.26-8.22 (m, 5H, N=C, H-3', H-5', Ar-Hm), 8.16 (m, 2H, H-2', H-6'), 6.99 (m, 2H, Ar-Hm) ppm. ^{13}C-NMR (125.7 MHz, DMSO-d6) δ 175.9 (CS), 147.9 (C-4'), 145.2 (C=N), 143.7 (C-1), 141.4 (C-4), 140.1 (C-1') 128.7 (C-2, C-6), 124.9 (C-2', C-6'), 123.8 (C-3, C-5), 123.7 (C-3', C-5') ppm; HRESI-MS *m/z* calcd. for $C_{14}H_{12}O_4N_5S$ ([M + H]$^+$): 346.0605, found: 346.0603.

*1-(4'-Hydroxybenzylidene)-4-(α-naphthyl)-3-thiosemicarbazone (**63**).* 4-(α-Naphthyl)-3-thiosemicarbazide **31** (500 mg, 2.30 mmol) and *p*-hydroxybenzaldehyde (280 mg, 2.30 mmol) were used. Yield: 280 mg (38%). Mp: 180-183 ºC. ^{1}H-NMR (300 MHz, DMSO-*d*$_6$) δ 11.73 (s, 1H, NH), 10.26 (s, 1H, Ar-NH), 9.92 (s, 1H, OH), 8.11 (s, 1H, N=CH), 7.96–7.52 (m 7H, naphthyl) 7.75 (m, 2H, H-2', H-6'), 6.79 (m, 2H, H-3', H-5') ppm; ^{13}C-NMR (125.7 MHz, DMSO-*d*$_6$) 177.6 (C=S), 159.6 (C-4), 143.3 (C=N), 135.9 (C-1) 133.9 (C-10), 130.8 (C-5), 129.6 (C-2', C-6'), 128.1 (C-3), 127.0 (C-1'), 126.6 (C-6), 126.2 (C-7), 125.8 (C-9) 125.6 (C-8), 125.3 (C-4), 123.6 (C-2), 115.7 (C-3', C-5') ppm; HRESI-MS *m/z* calcd. for $C_{18}H_{16}N_3OS$ ([M + H]$^+$): 322.1009, found: 322.1009.

*1-(3'-4'-Dihydroxybenzylidene)-4-(α-naphthyl)-3-thiosemicarbazone (**65**).* 4-(α-Naphthyl)-3-thiosemicarbazide **31** (500 mg, 2.30 mmol) and 3,4-dihydroxybenzaldehyde (320 mg, 2.30 mmol) were used. Mp: 180-183 ºC. ^{1}H-NMR (300 MHz, DMSO-*d*$_6$) δ 11.68 (s, 1H, NH), 10.20 (s, 1H, Ar-NH), 9.68 (s, 1H, OH), 8.95 (s, 1H, OH), 8.03 (s, 1H, N=CH), 7.96–7.47 (m, 7H, H-naphthyl), 7.14 (d, 1H, $J_{2',6'}$= 1.9 Hz, H-2'), 7.12 (dd, 1H, $J_{2',6'}$= 1.9 Hz, $J_{5',6'}$= 8.2 Hz, H-6'), 6.76 (d, $J_{5',6'}$= 8.2 Hz, 1H, H-5') ppm; ^{13}C-NMR (125.7 MHz, DMSO-*d*$_6$) 177.3 (C=S), 147.9 (C-4'), 145.5 (C=N), 143.5 (C-5'), 135.7 (C-1) 133.7 (C-10), 130.5 (C-5), 128.0 (C-1'), 126.7 (C-3), 126.3 (C-6), 126.0 (C-7), 125.9 (C-9) 125.6 (C-8), 125.4 (C-4), 123.3 (C-2), 120.6 (C-2'), 115.4 (C-3'), 114.2 (C-6') ppm; HRESI-MS *m/z* calcd. for $C_{18}H_{16}N_3O_2S$ ([M + H]$^+$): 338.0958, found: 338.0959.

*General procedure for the preparation of selenosemicarbazones **74**, **75**.*

A solution of selenosemicarbazides **72**, **73** (150 mg), the corresponding aromatic aldehyde (1.0 equiv.) and AcOH (1.0 equiv.) in EtOH (5 mL) was refluxed for 4 h. After cooling to rt, the products precipitated and were washed with cold EtOH.

4-(4'-Bromophenyl)-1-(3'',4''-dihydroxybenzylidene)-3-selenosemicarbazone ***(74)***. 4-(4'-Bromophenyl)-3-selenosemicarbazide **72** (150 mg, 0.52 mmol), 3,4.dihydroxybenzaldehyde (70 mg, 0.52 mmol, 1.0 equiv.) and AcOH (30 µL, 0.52 mmol) were used. Yield: 88 mg (42%). ^{1}H-NMR (300 MHz, DMSO-d_6) δ: 11.98 (s, 1H, NH), 10.30 (s, 1H, Ar-NH), 9.33 (s, 1H, OH), 8.98 (s, 1H, OH) 8.13 (s, 1H, N=CH), 7.48 (m, 4H, Ar-H*o*, Ar-H*m*, H-2', H-6'), 7.34 (d, 1H, $J_{2'',6''}$= 2..2 Hz, H-2''), 7.12 (dd, 1H, $J_{2'',6''}$= 2..2 Hz, H-2'', $J_{5'',6''}$= 8.2 Hz, H-6''), 6.77 (d, 1H, $J_{5'',6''}$= 8.2 Hz, H-5'') ppm; ^{13}C-NMR (125.7 MHz, DMSO-d_6) δ 173.7 (CSe), 148.8 (C-4'), 146.1 (C-3'), 139.8 (C=N), 131.3 (C-1), 128.9 (C-4), 125.66 (C-2, C-6), 121.5 (C-1'), 120.9 (C-6'), 118.5 (C-3, C-5), 116.0 (C-5'), 114.8 (C-3') ppm; HRESI-MS *m/z* calcd. for $C_{12}H_{12}{}^{79}BrN_3O_2{}^{80}Se$ ([M+H]$^+$): 413.9351, found: 413.9345; *m/z* calcd. for $C_{12}H_{12}{}^{81}BrN_3O_2{}^{80}Se$ ([M + H]$^+$): 415.9329, found: 415.9330.

4-(4'-Iodophenyl)-1-(4''-methoxybenzylidene)-3-selenosemicarbazone ***(75)***. 4-(4'-Iodophenyl)-3-selenosemicarbazide **73** (150 mg, 0.30 mmol), *p*-anisaldehyde (53 µL, 0.30 mmol, 1.0 equiv.) and AcOH (25 µL, 0.30 mmol) were used. Yield: 33 mg (16%). ^{1}H-NMR (300 MHz, DMSO-d_6) δ 12.10 (s, 1H, NH), 10.37 (s, 1H, Ar-NH), 8.23 (s, 1H, N=CH), 7.87 (m, 2H, H-2', H-6'), 7.72 (m, 2H, Ar-H*o*), 7.38 (m, 2H, Ar-H*m*), 6.99 (m, 2H, H-3', H-5'), 3.81 (s, 3H, CH_3) ppm; ^{13}C-NMR (125.7 MHz, DMSO-d_6) δ 173.5 (CSe), 161.1 (C-4''), 144.6 (C=N), 139.8 (C-1'), 136.7 (C-2', C-6'), 129.6 (C-2'', C-6''), 128.8 (C-3', C-5'), 126.3 (C-1''), 114.2 (C-3'', C-5''), 90.7 (C-4'), 55.3 (OMe) ppm; HRESI-MS *m/z* calcd. for $C_{15}H_{14}IN_3NaO^{80}Se$ ([M + Na]$^+$): 481.9239, found: 481.9230.

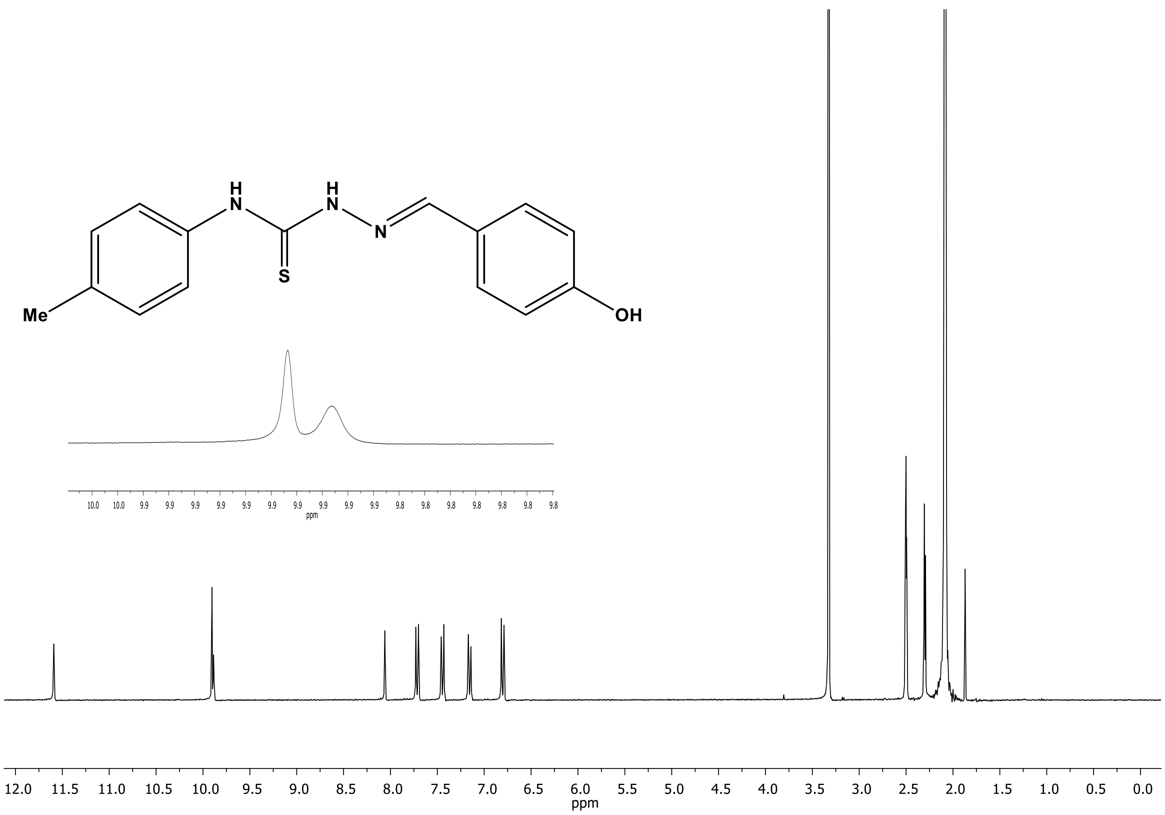

^{1}H-NMR (300 MHz, DMSO-d_6) of **39**

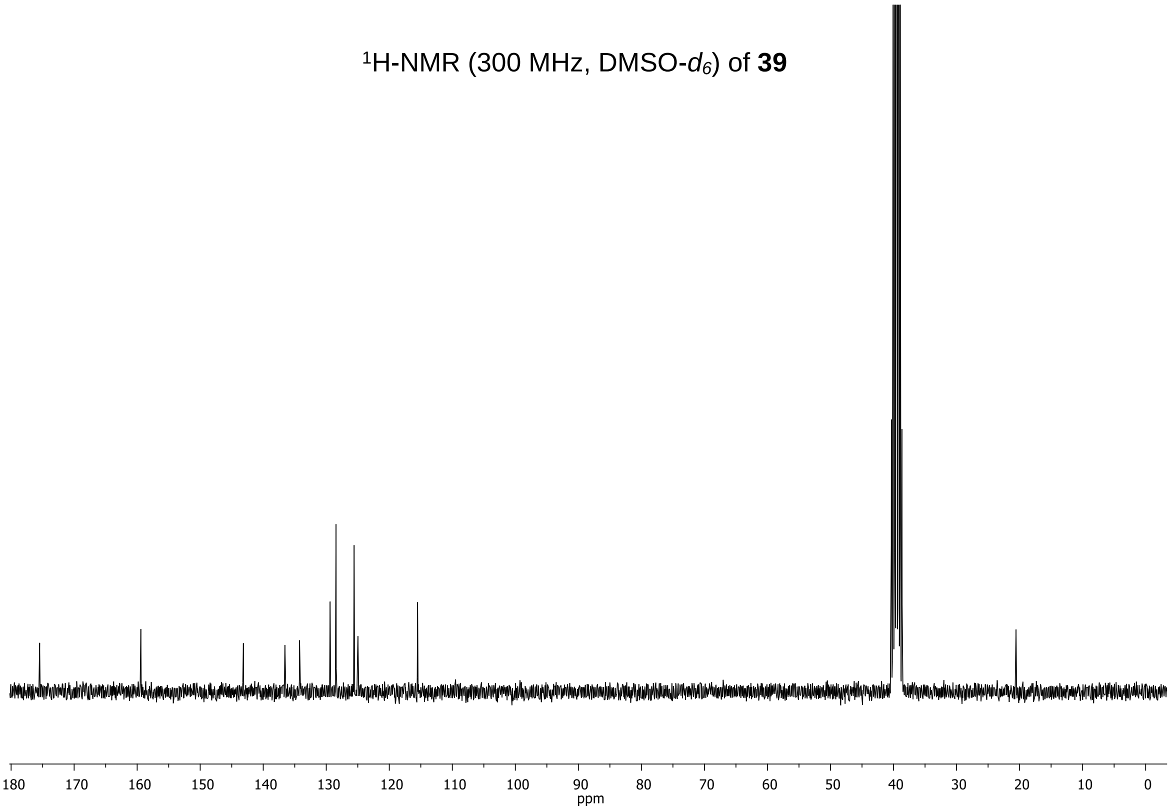

^{13}C-NMR (125.7 MHz, DMSO-d_6) of **39**

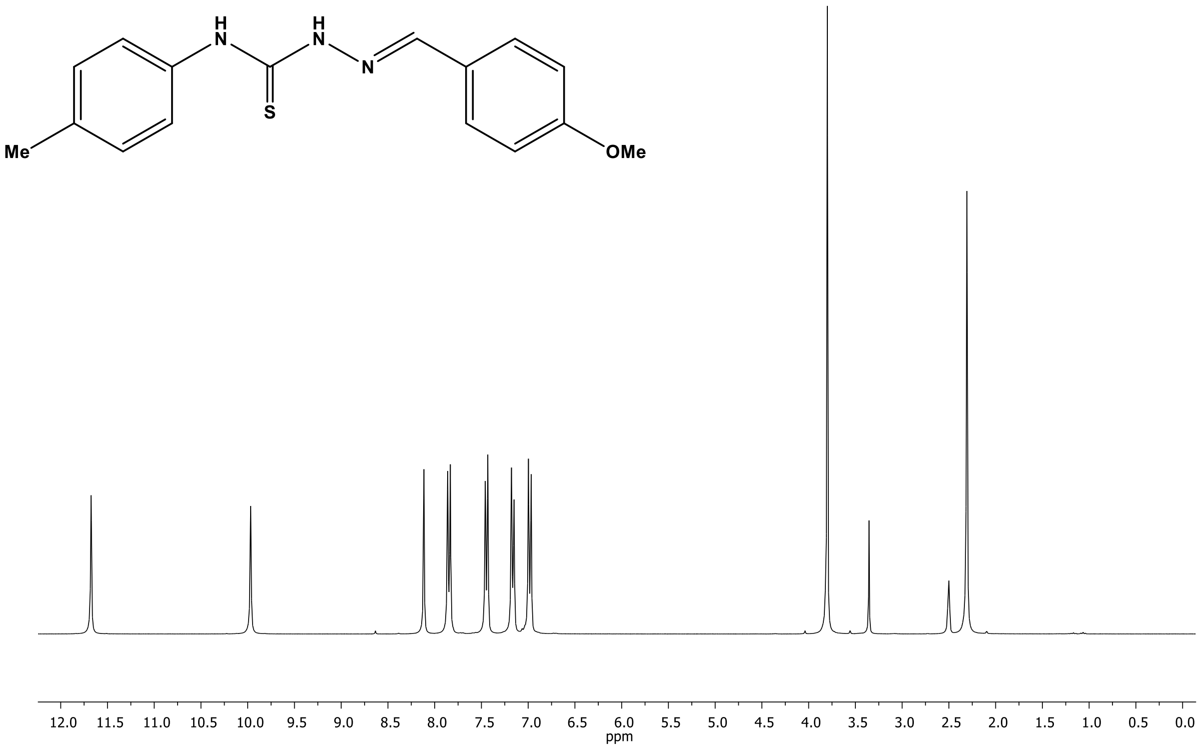

^{1}H-NMR (300 MHz, DMSO-d_6) of **40**

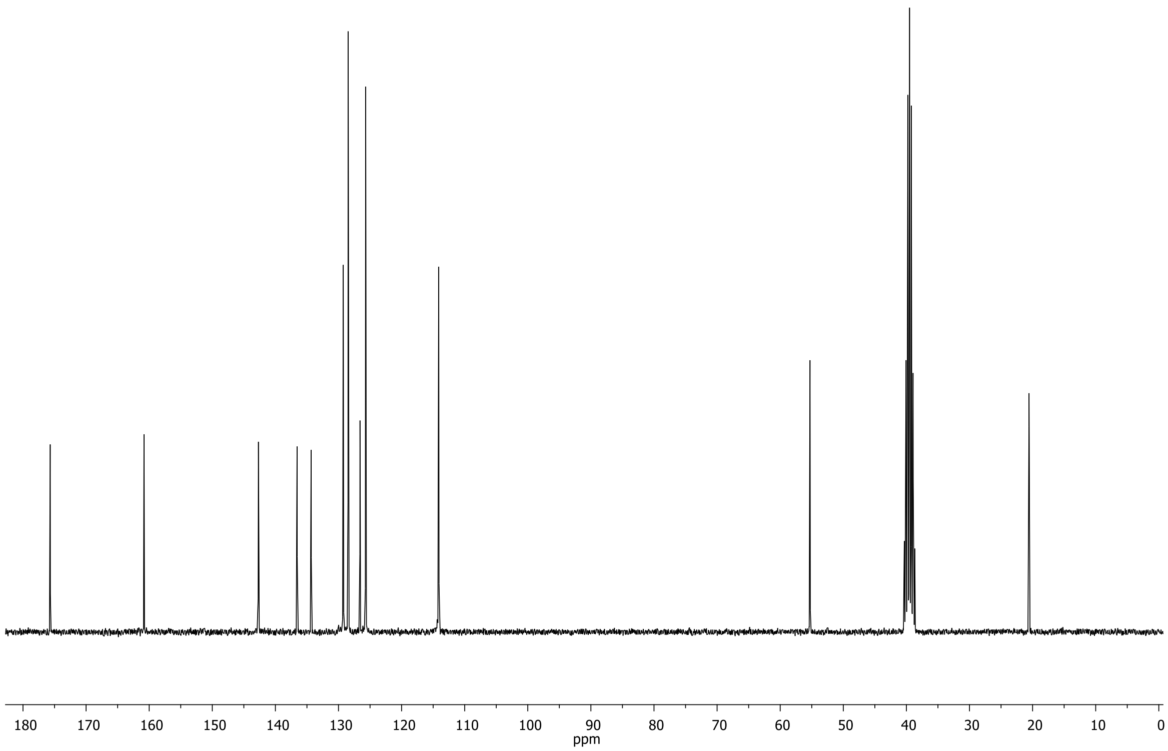

^{13}C-NMR (125.7 MHz, DMSO-d_6) of **40**

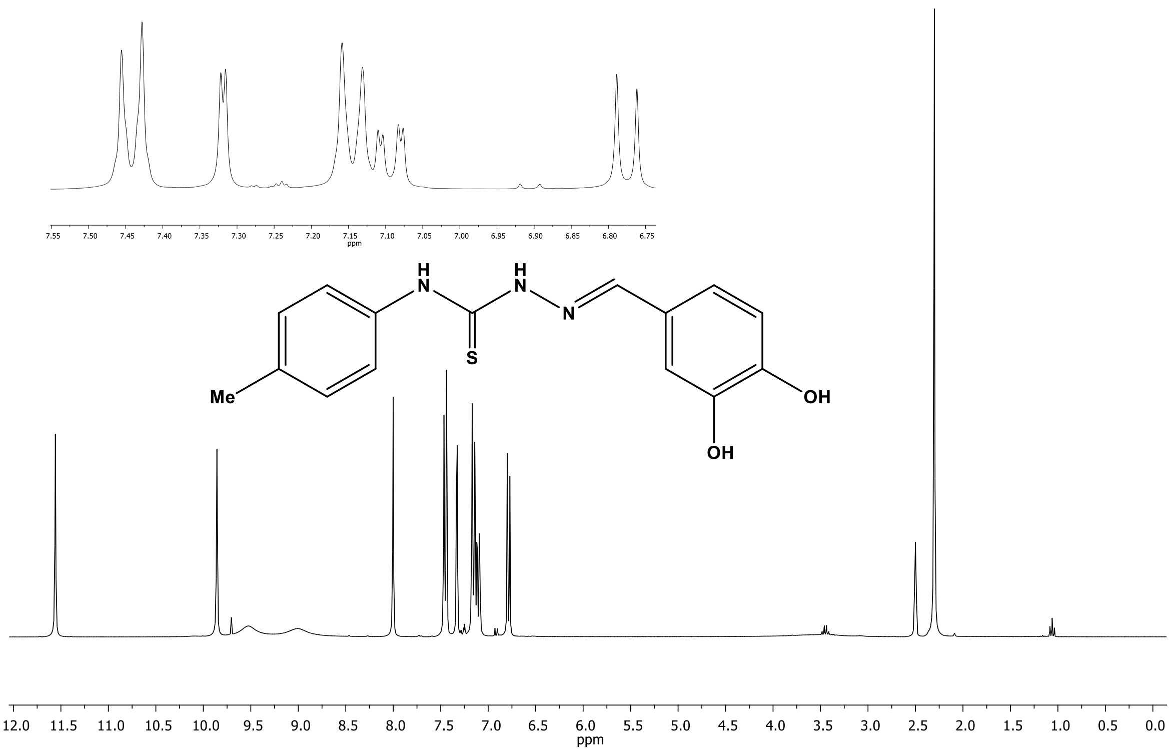

^{1}H-NMR (300 MHz, DMSO-d_6) of **41**

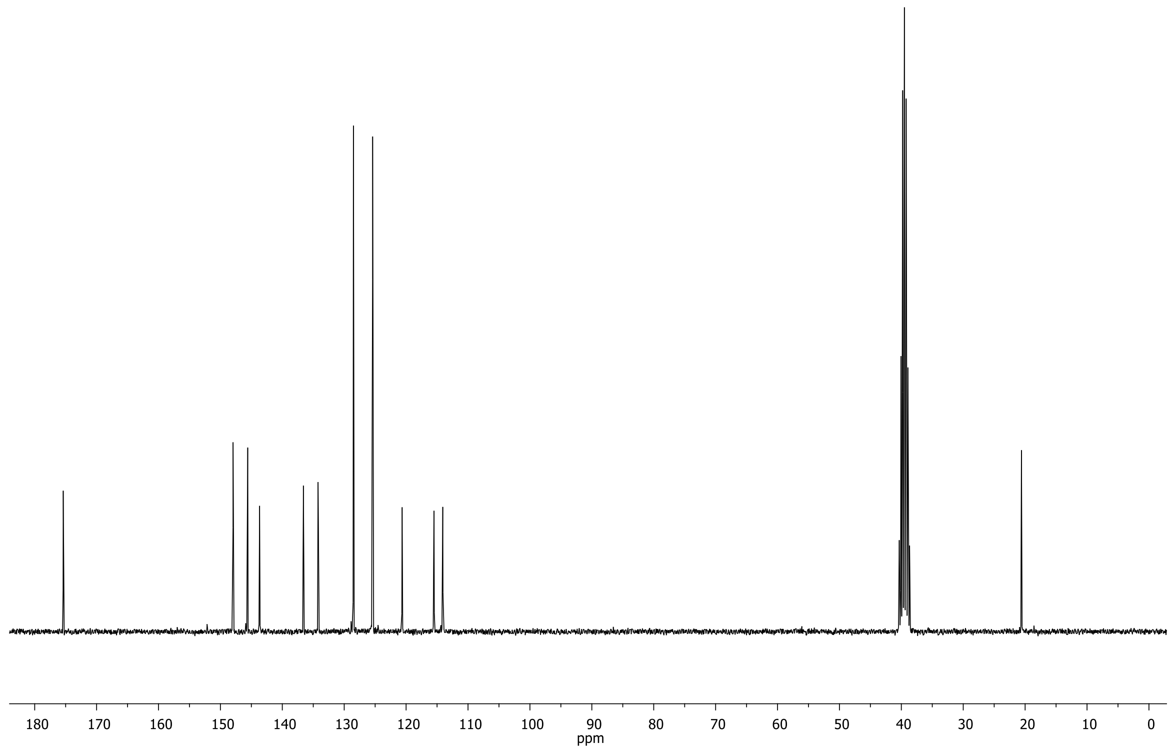

^{13}C-NMR (125.7 MHz, DMSO-d_6) of **41**

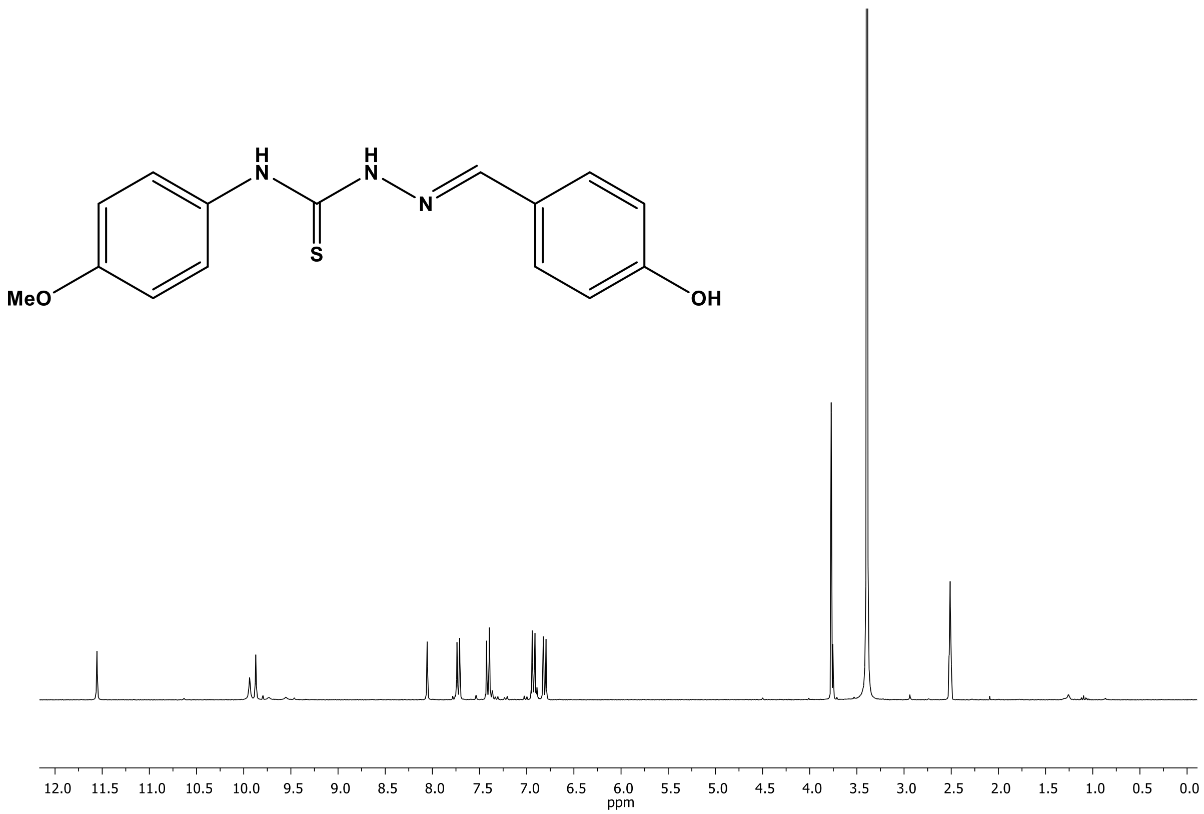

^{1}H-NMR (300 MHz, DMSO-d_6) of **42**

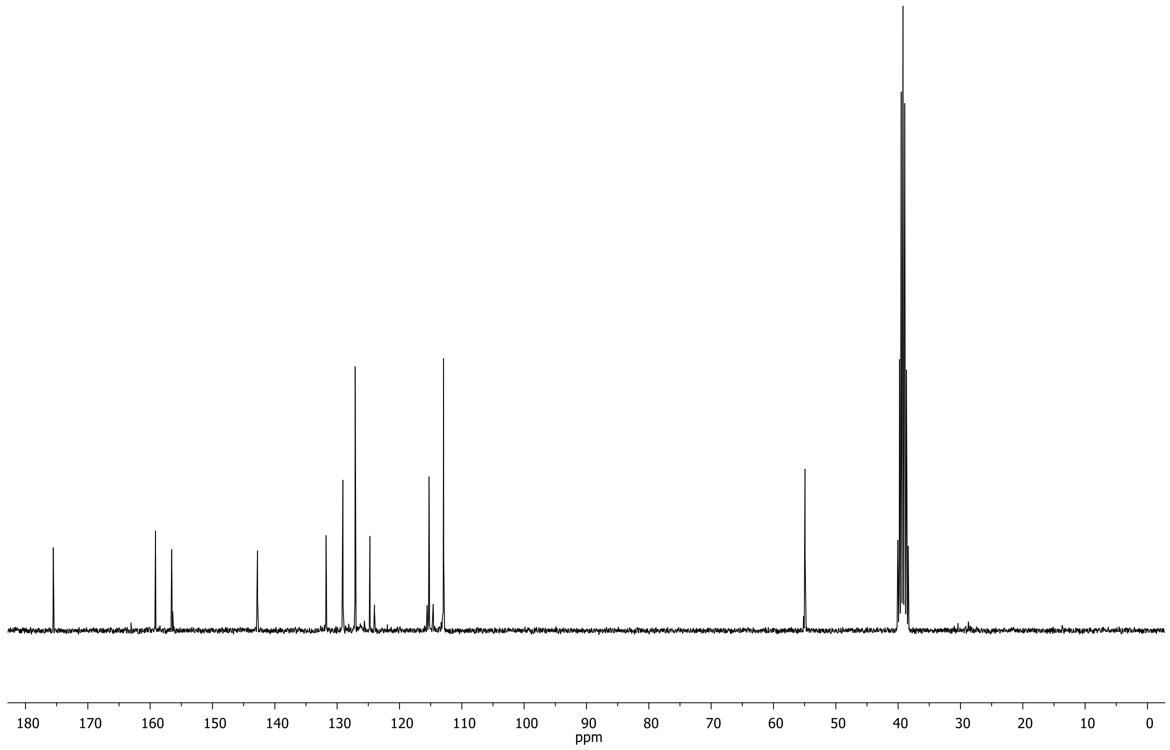

^{13}C-NMR (125.7 MHz, DMSO-d_6) of **42**

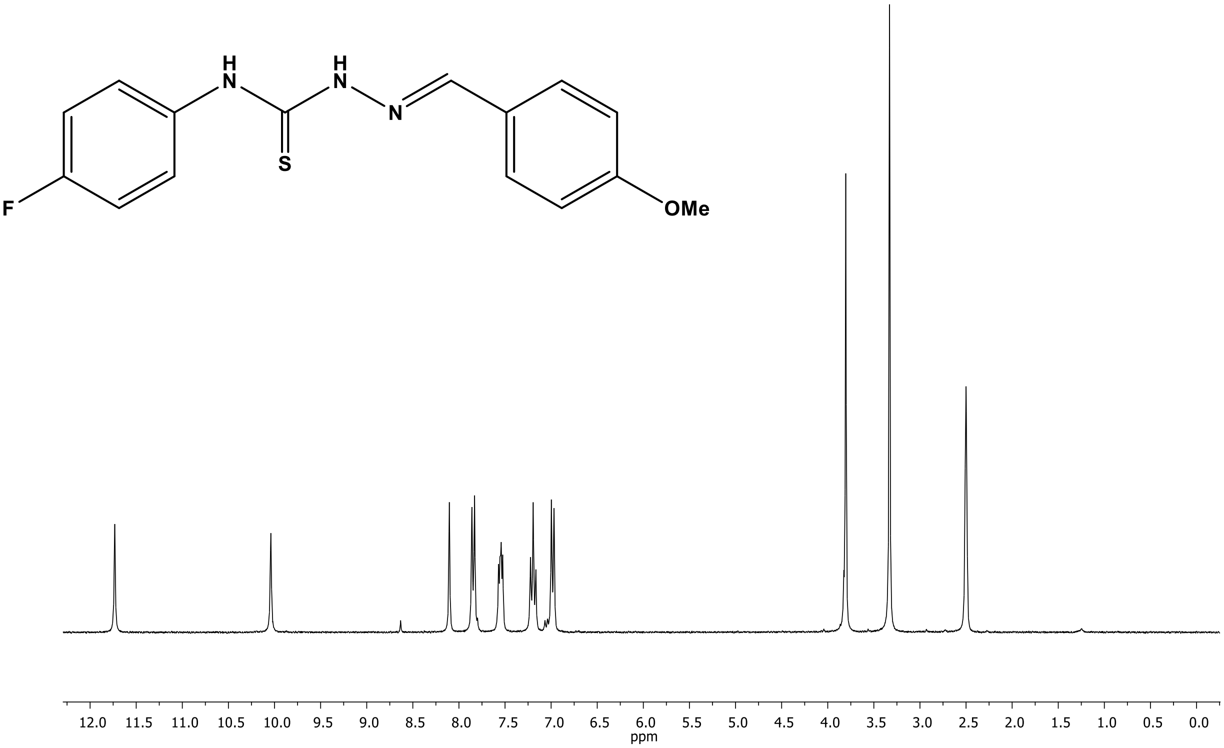

^{1}H-NMR (300 MHz, DMSO-d_6) of **44**

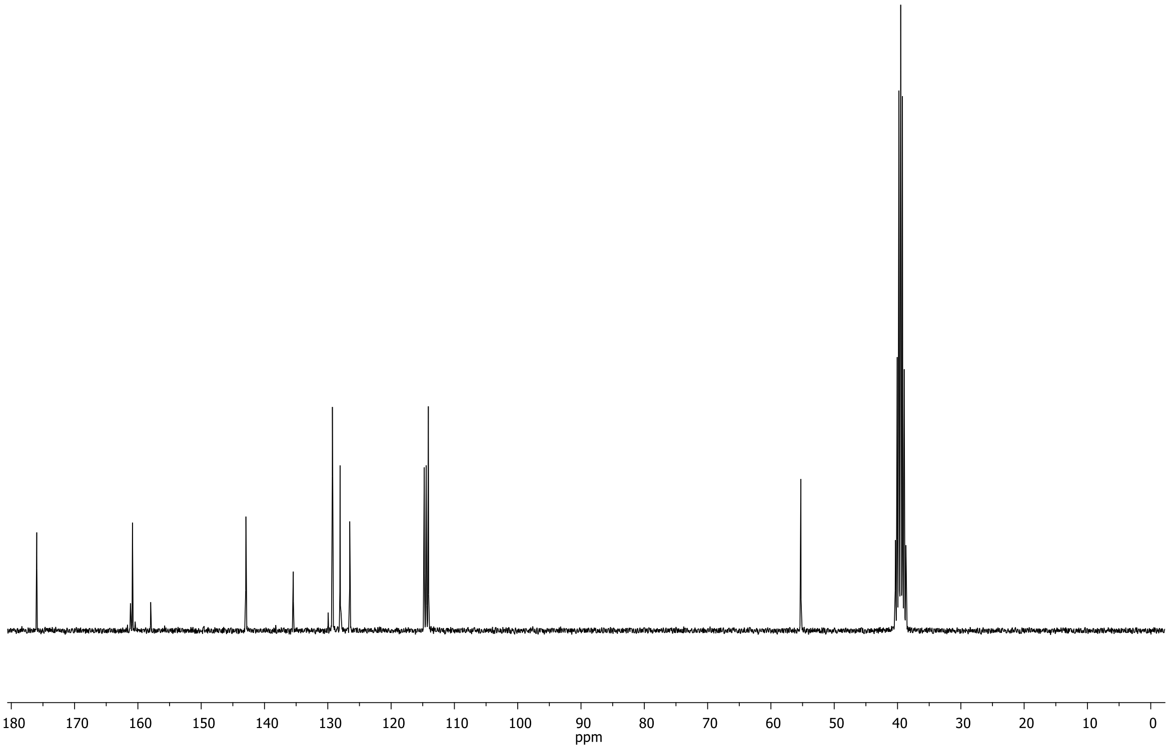

^{13}C-NMR (125.7 MHz, DMSO-d_6) of **44**

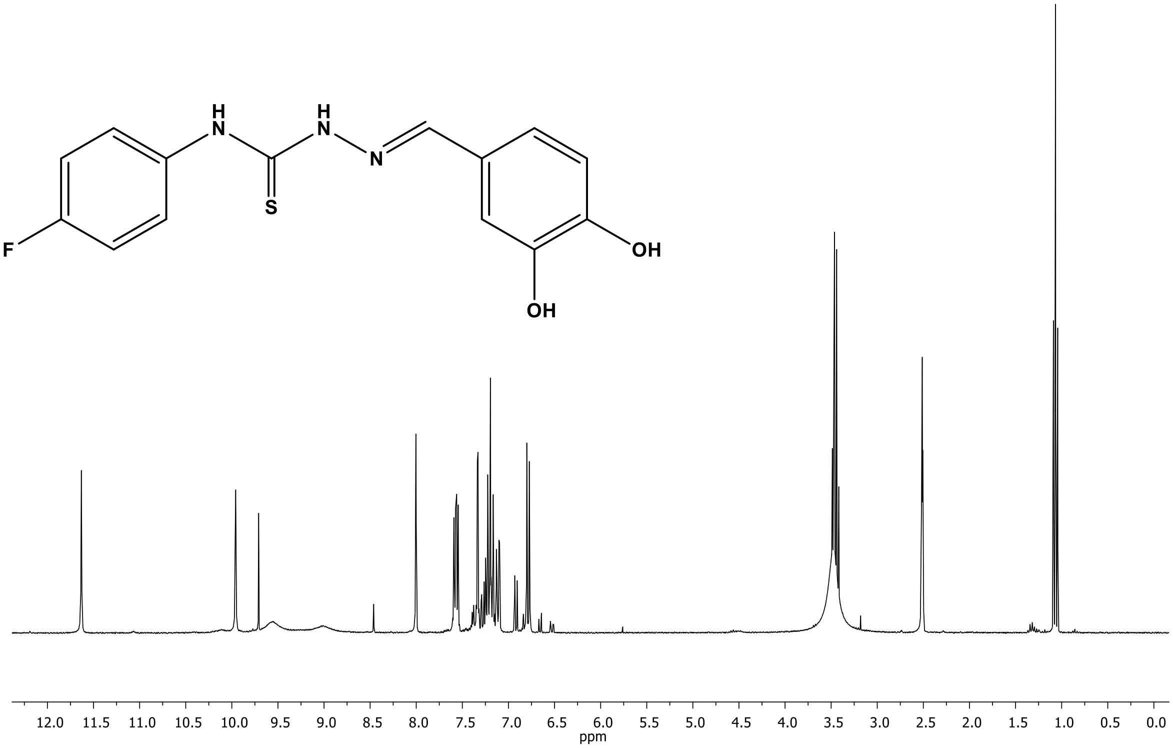

^{1}H-NMR (300 MHz, DMSO-d_6) of **45**

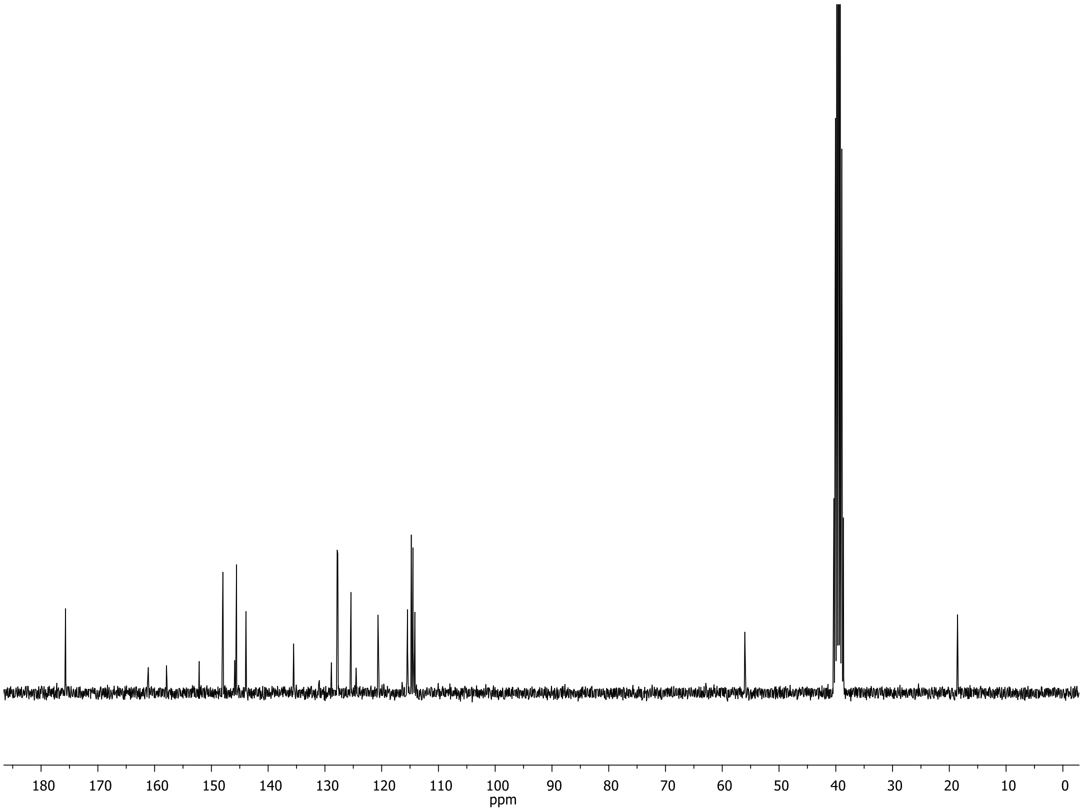

^{13}C-NMR (125.7 MHz, DMSO-d_6) of **45**

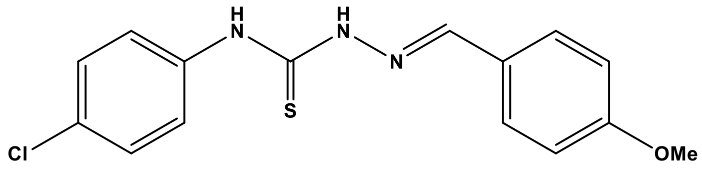

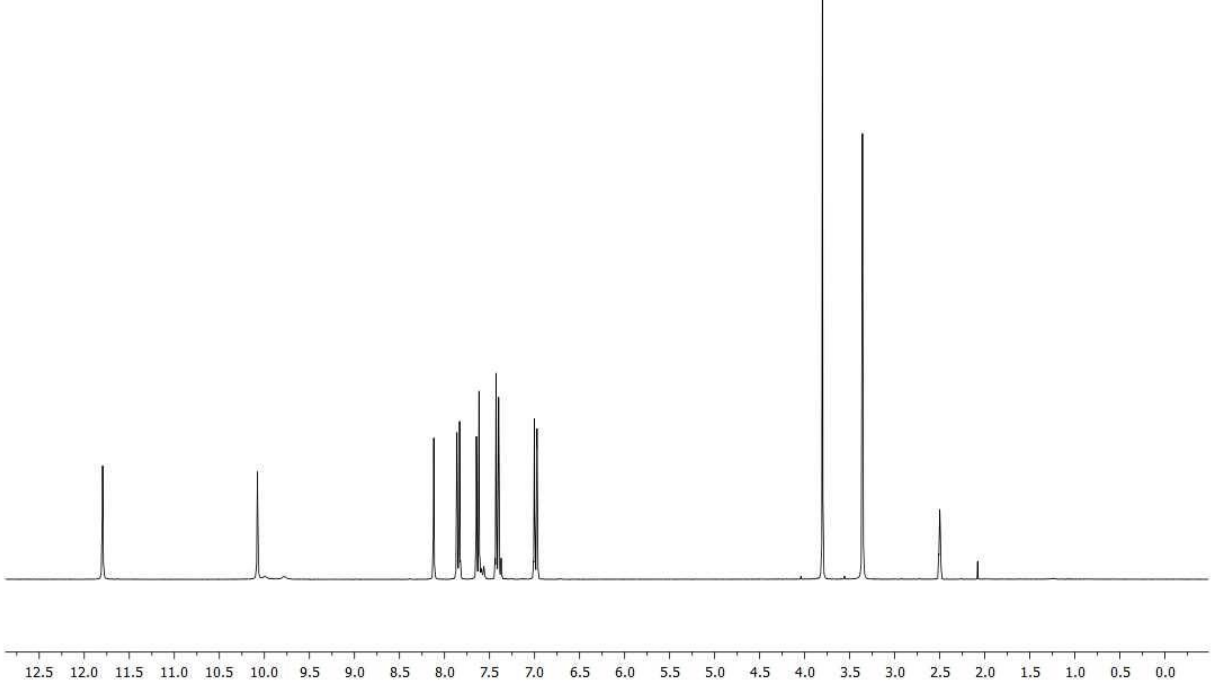

^{1}H-NMR (300 MHz, DMSO-d_6) of **46**

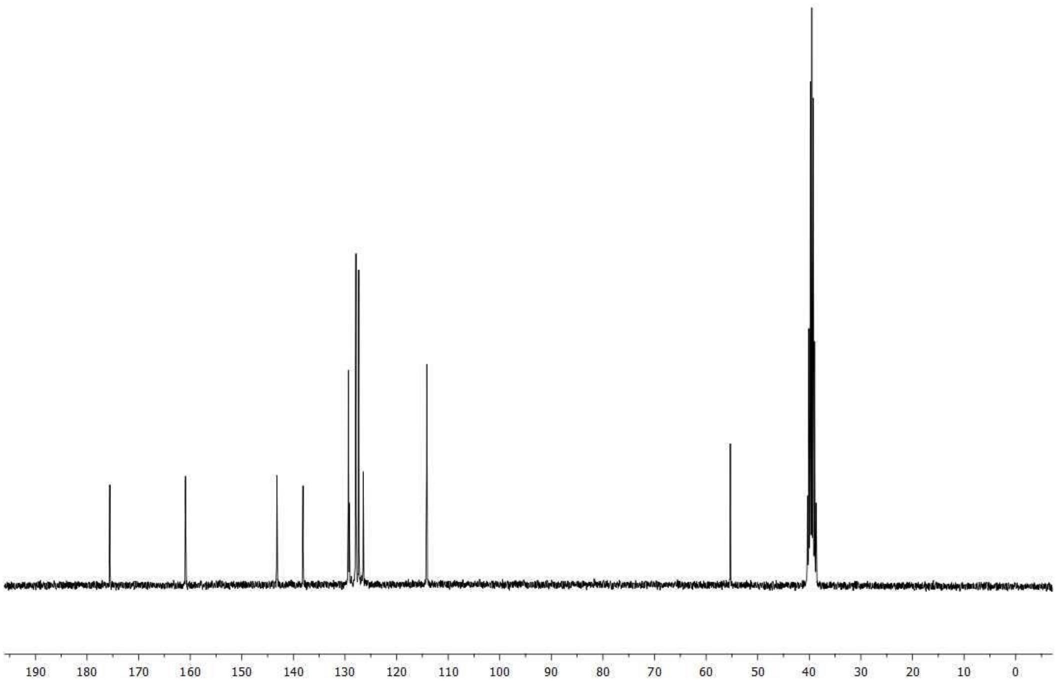

^{13}C-NMR (125.7 MHz, DMSO-d_6) of **46**

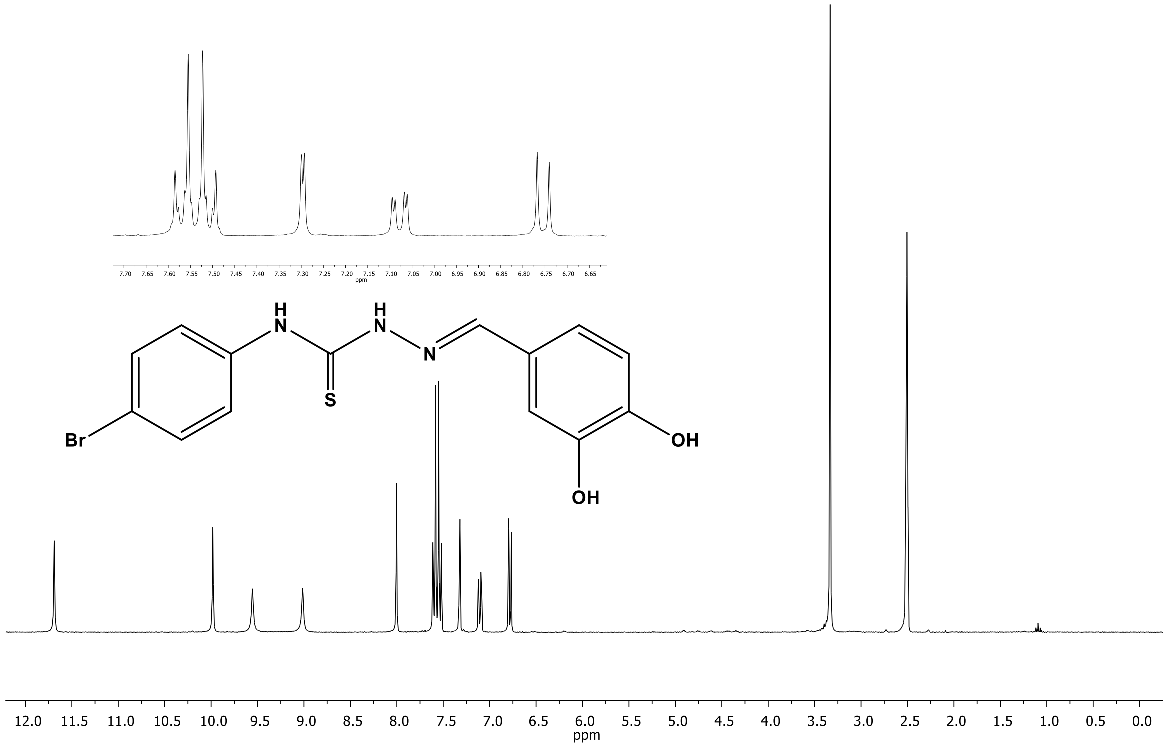

^{1}H-NMR (300 MHz, DMSO-d_6) of **49**

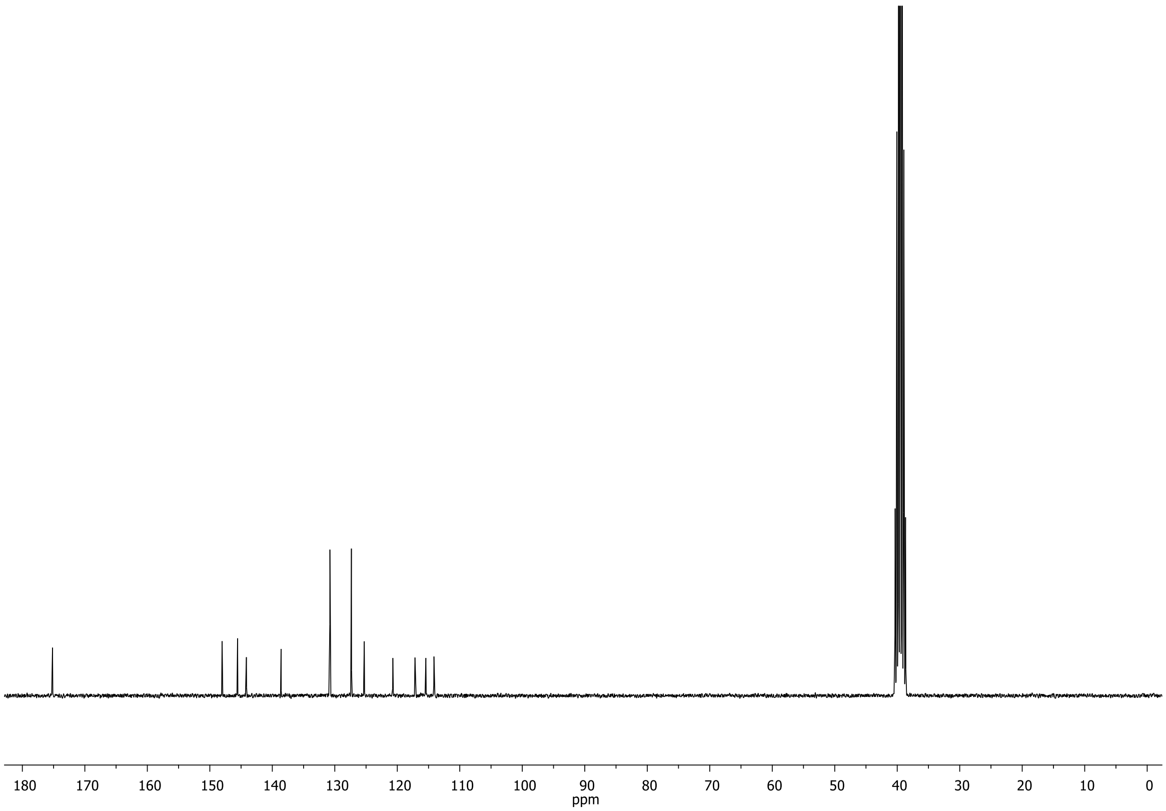

^{13}C-NMR (125.7 MHz, DMSO-d_6) of **49**

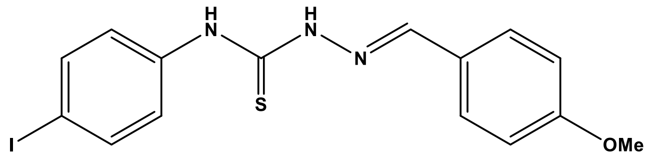

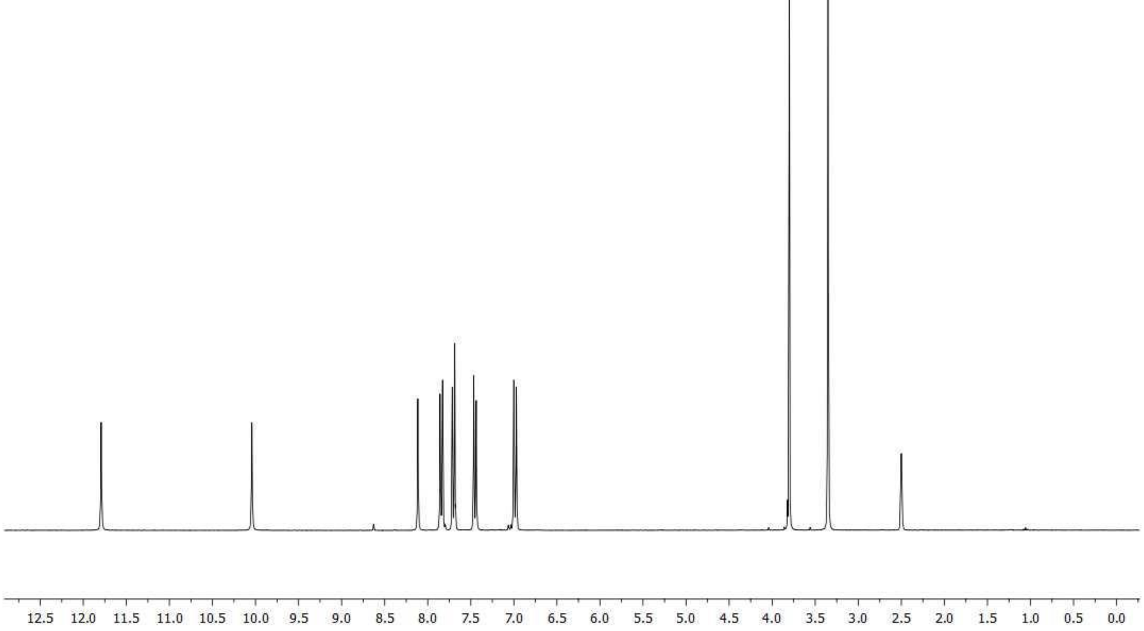

^{1}H-NMR (300 MHz, DMSO-d_6) of **51**

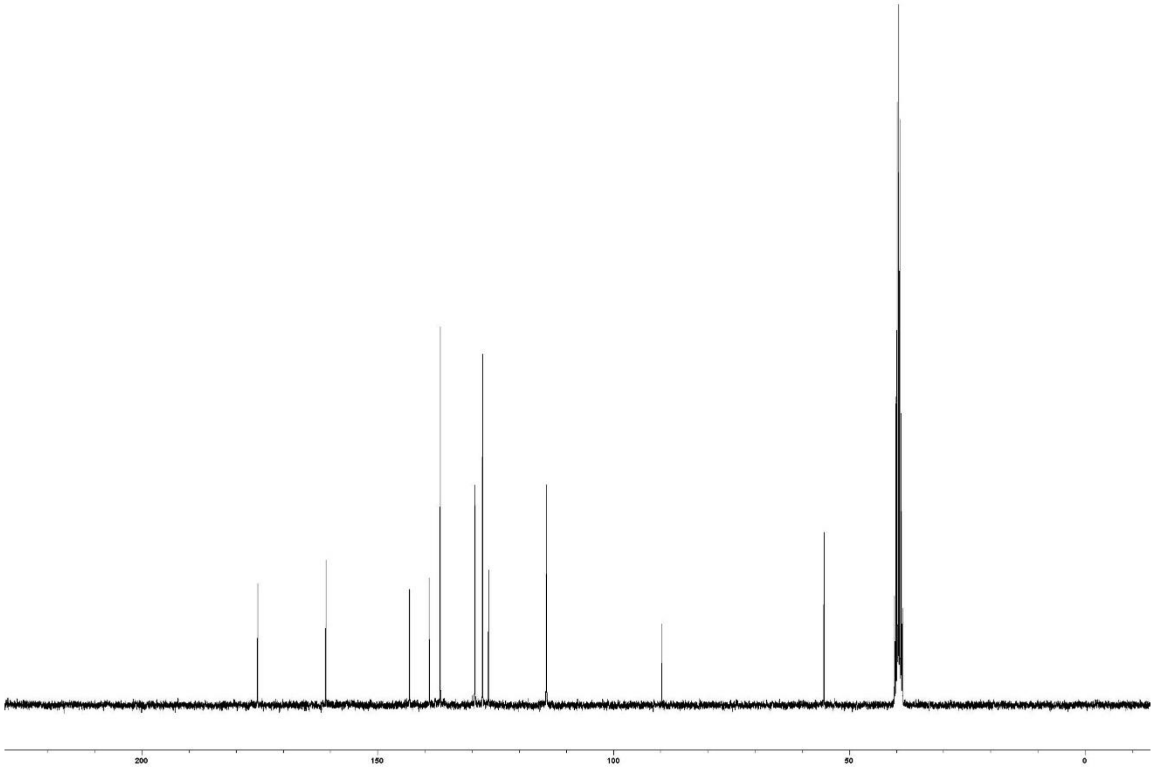

^{13}C-NMR (125.7 MHz, DMSO-d_6) of **51**

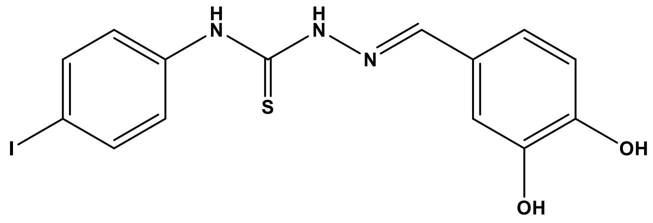

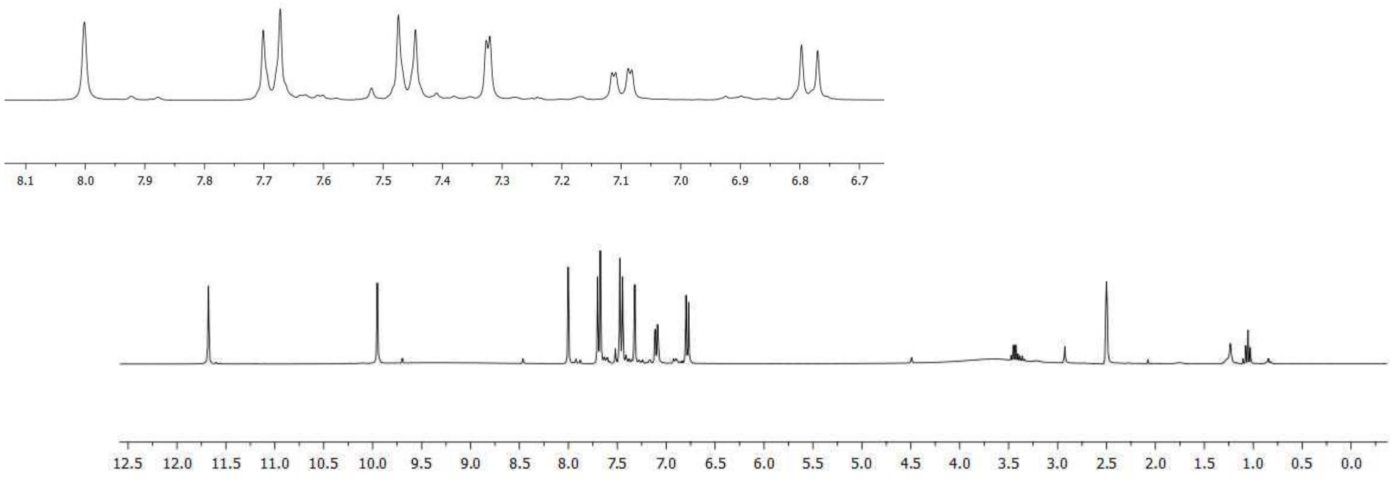

^{1}H-NMR (300 MHz, DMSO-d_6) of **52**

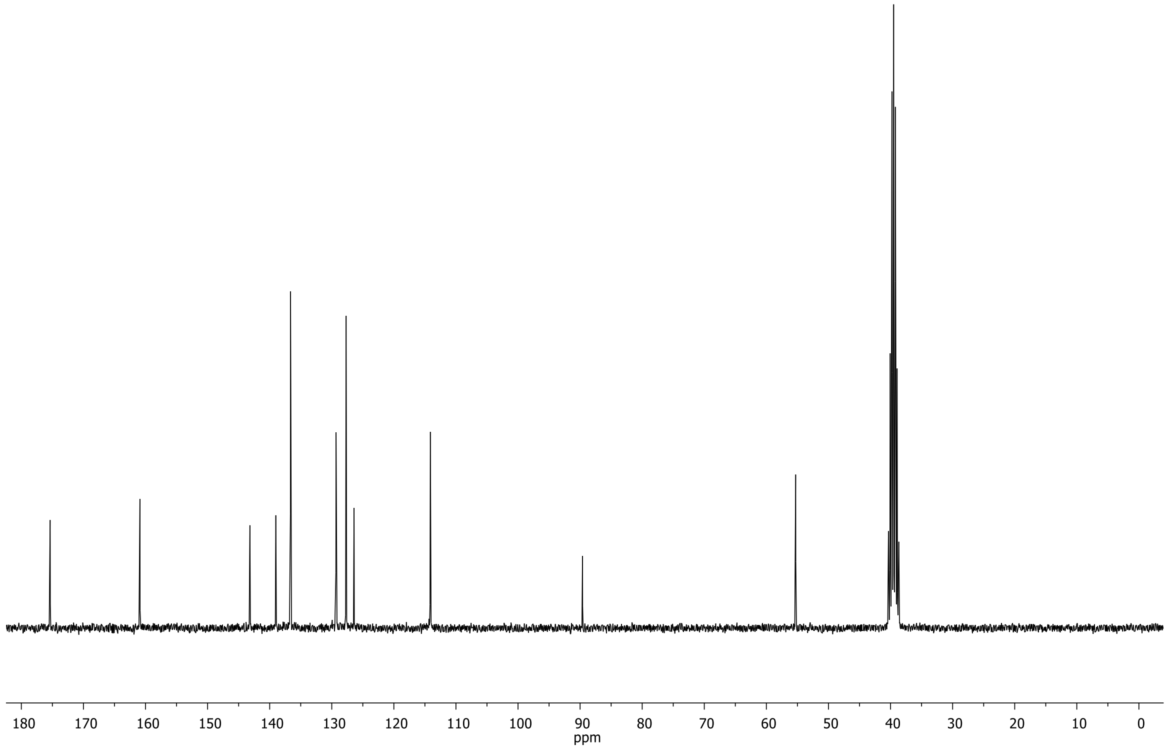

^{13}C-NMR (125.7 MHz, DMSO-d_6) of **52**

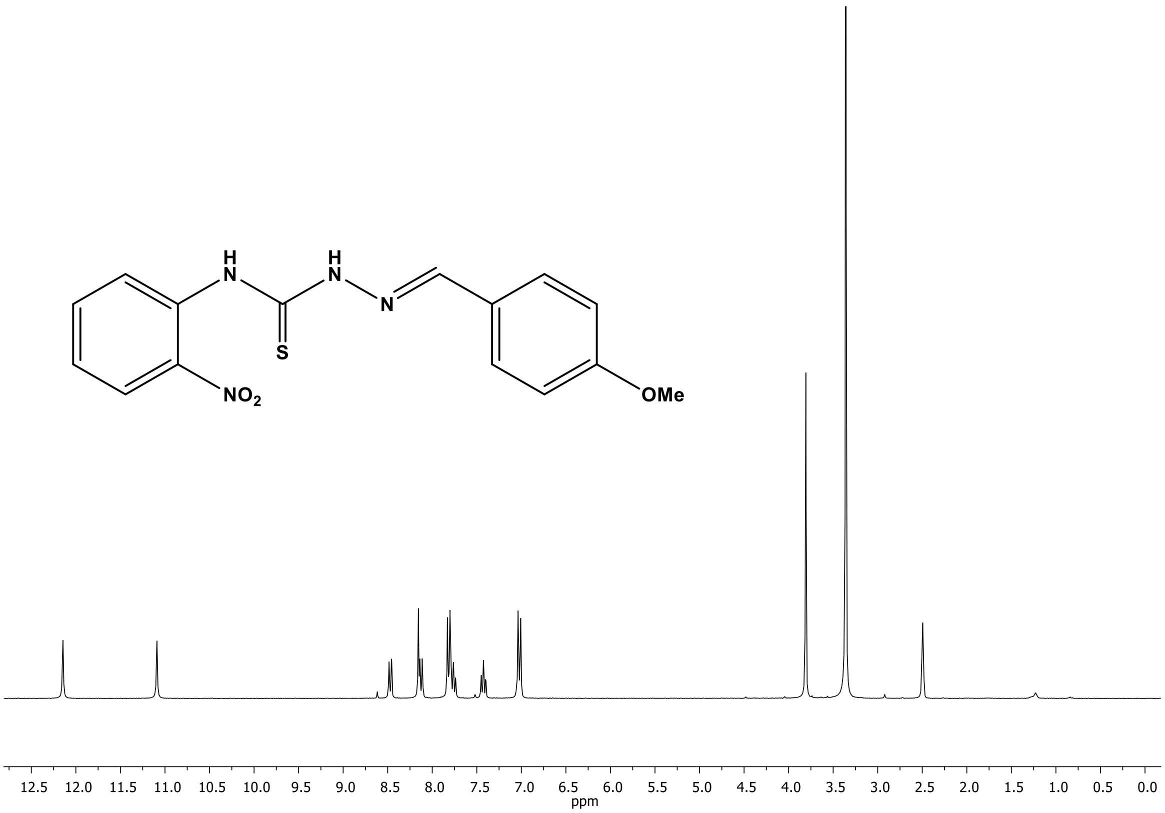

^{1}H-NMR (300 MHz, DMSO-d_6) of **54**

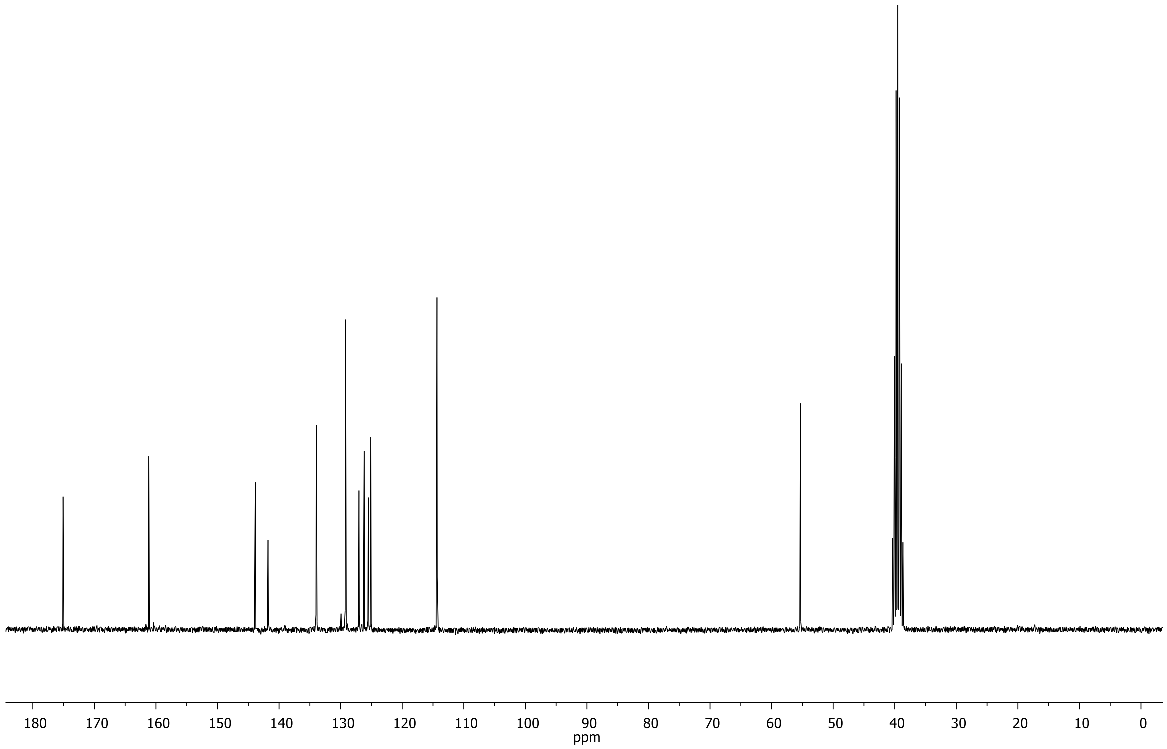

^{13}C-NMR (125.7 MHz, DMSO-d_6) of **54**

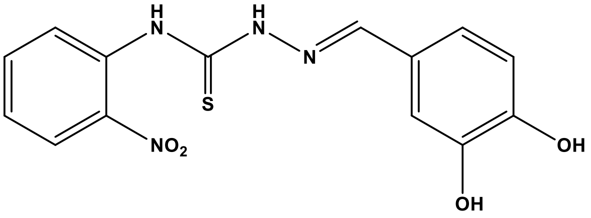

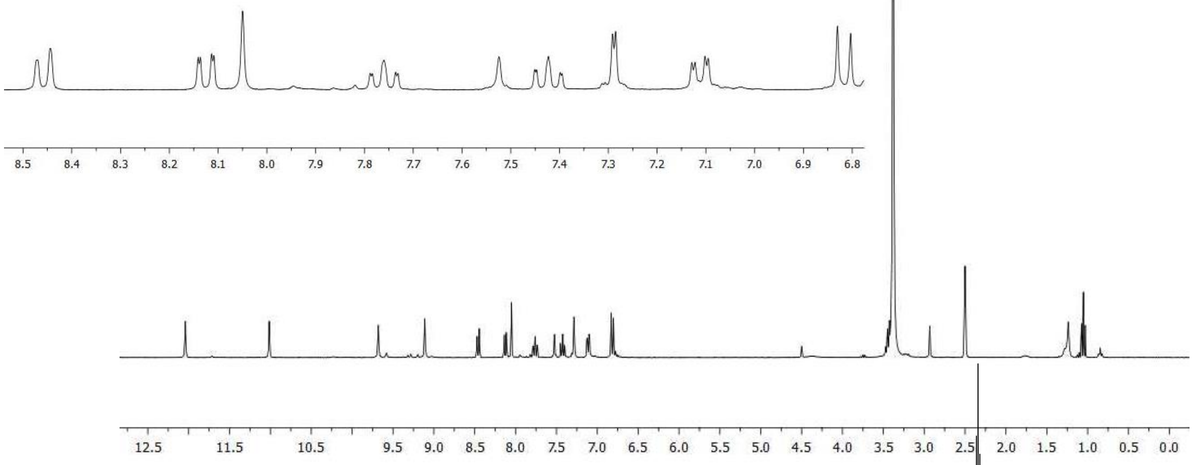

^{1}H-NMR (300 MHz, DMSO-d_6) of **55**

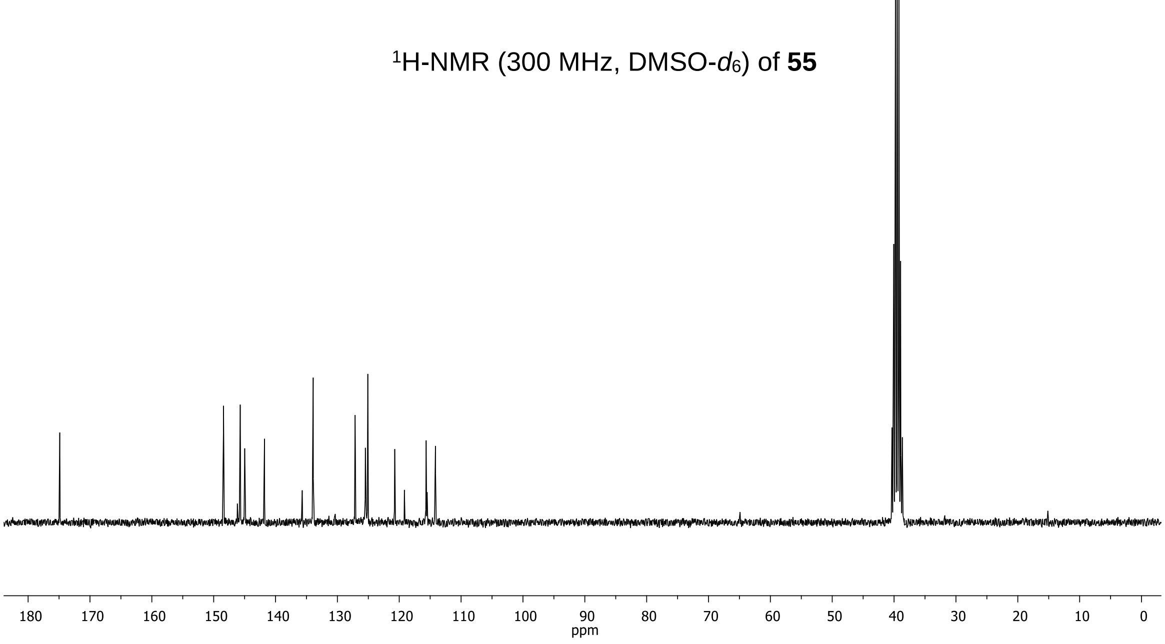

^{13}C-NMR (125.7 MHz, DMSO-d_6) of **55**

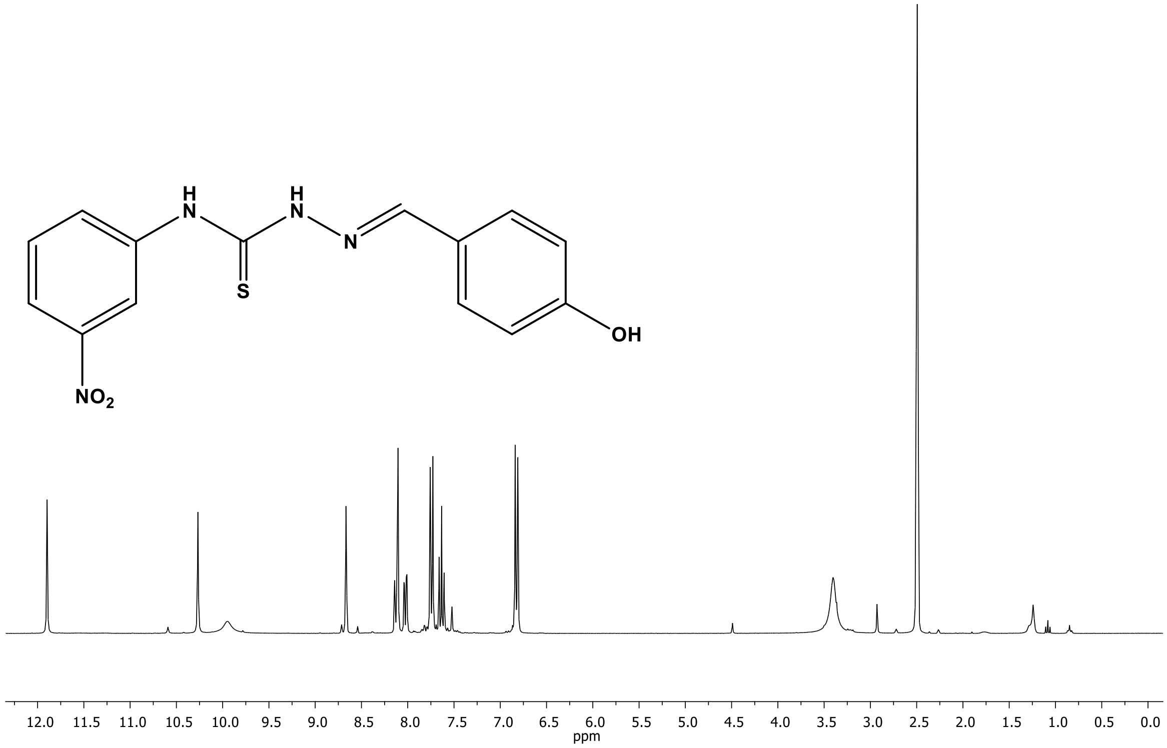

^{1}H-NMR (300 MHz, DMSO-d_6) of **56**

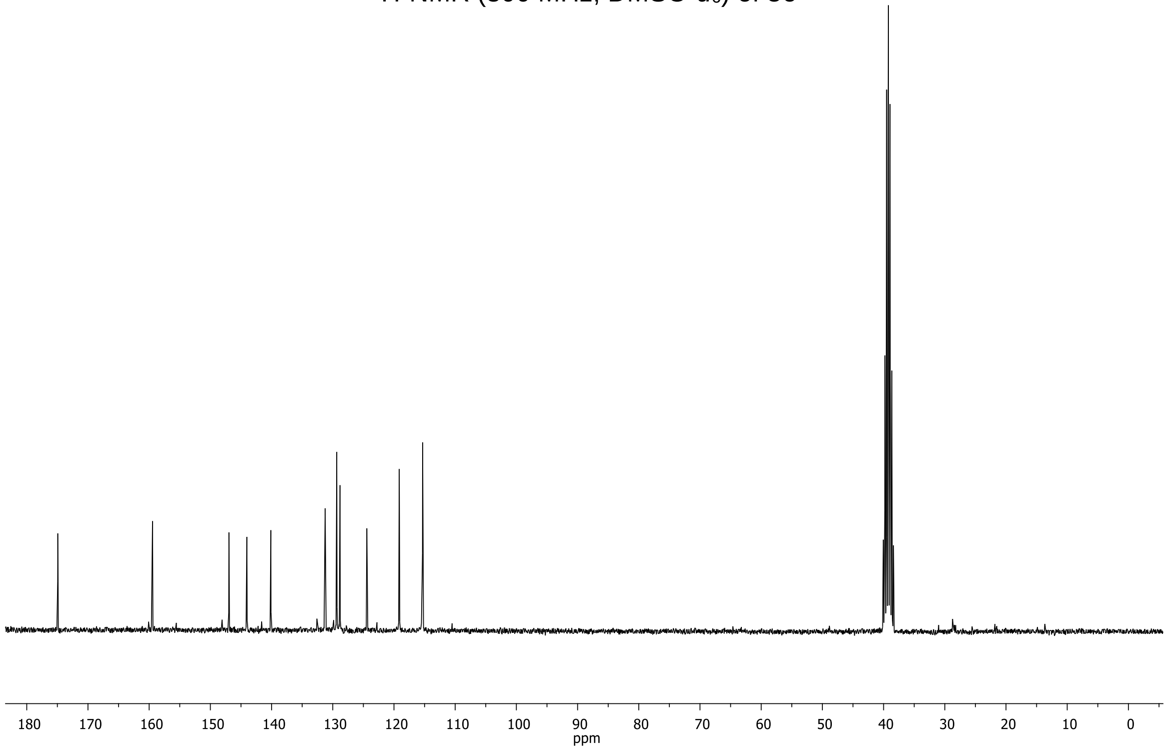

^{13}C-NMR (125.7 MHz, DMSO-d_6) of **56**

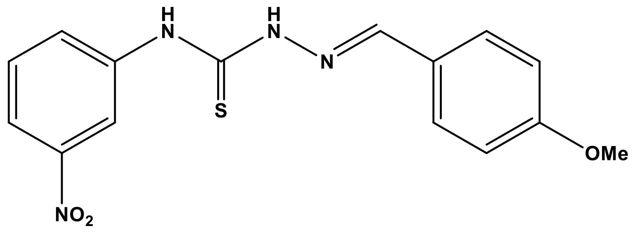

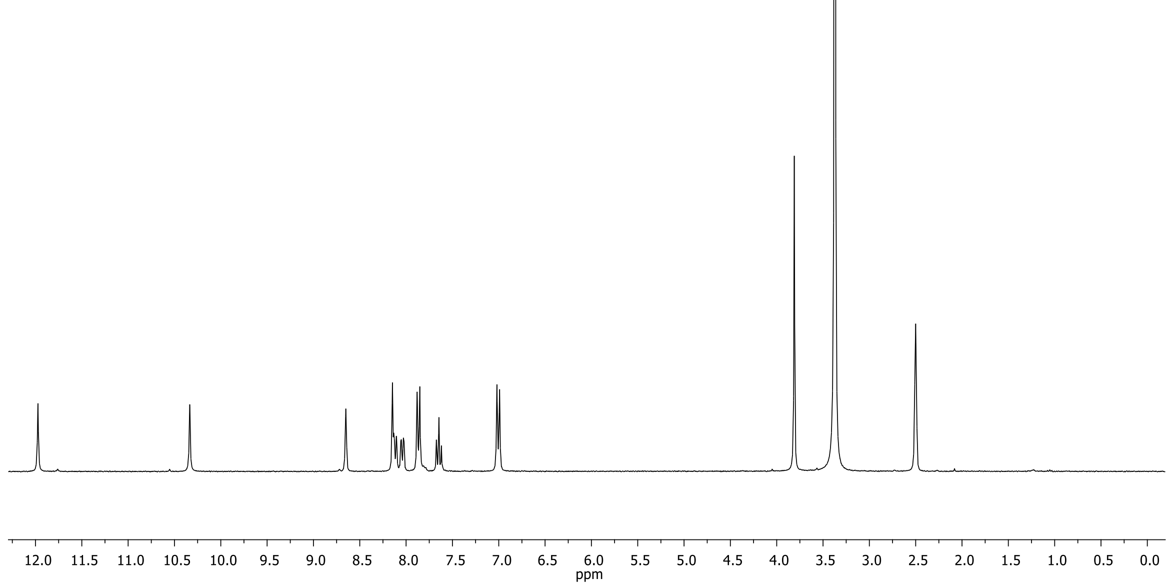

^{1}H- (300 MHz, DMSO-d_6) of **57**

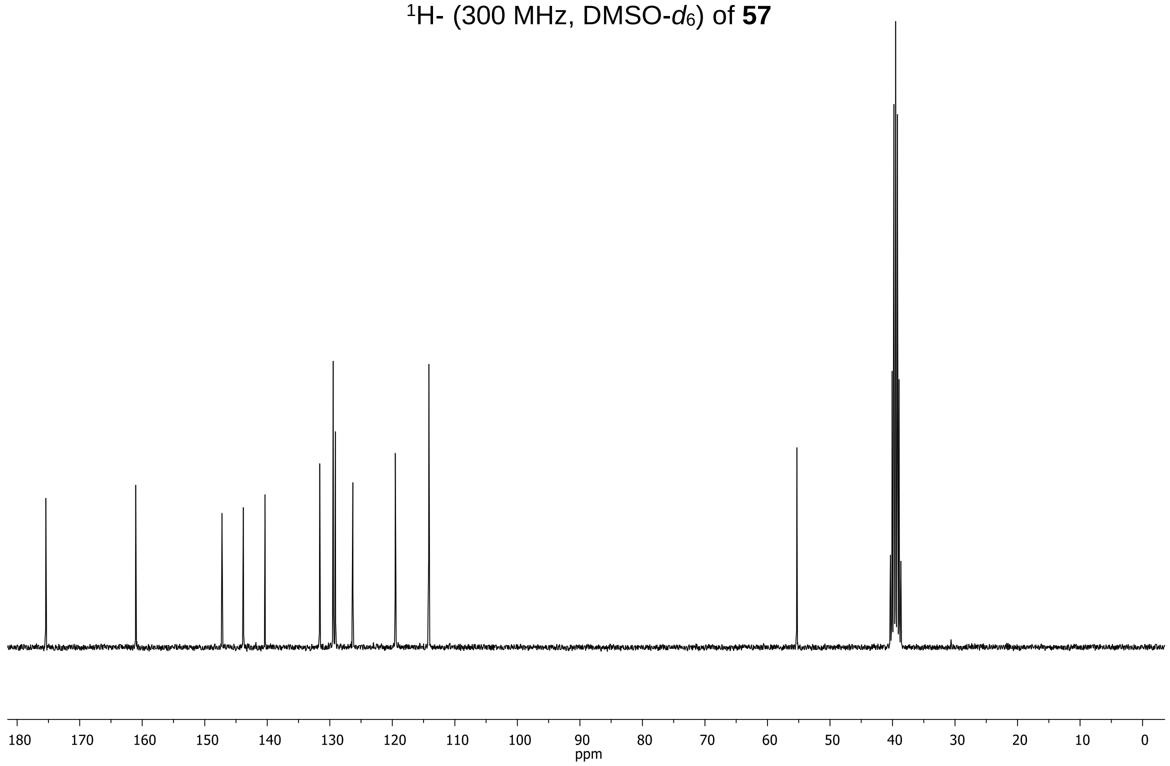

^{13}C-NMR (125.7 MHz, DMSO-d_6) of **57**

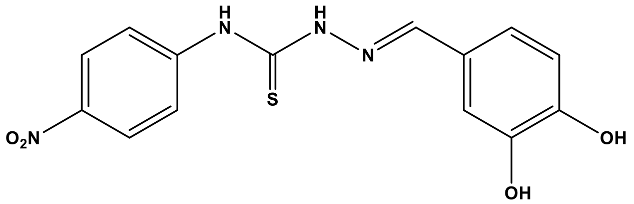

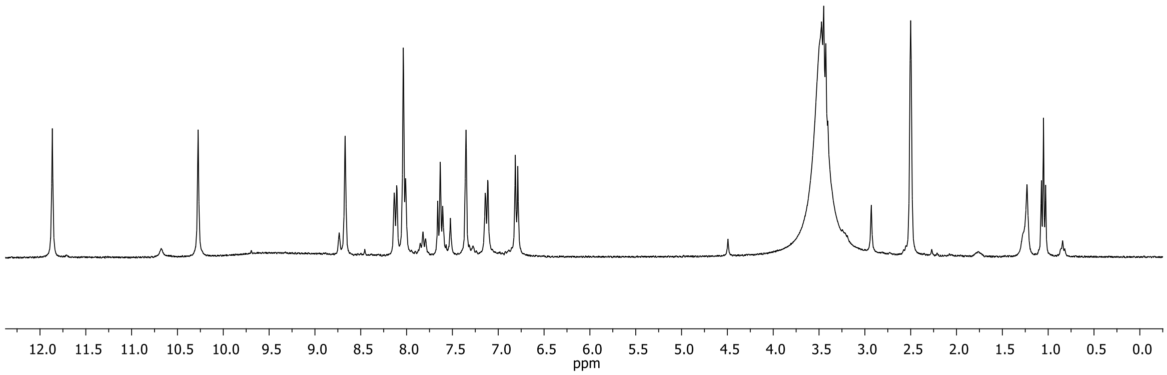

^{1}H-NMR (300 MHz, DMSO-d_6) of **58**

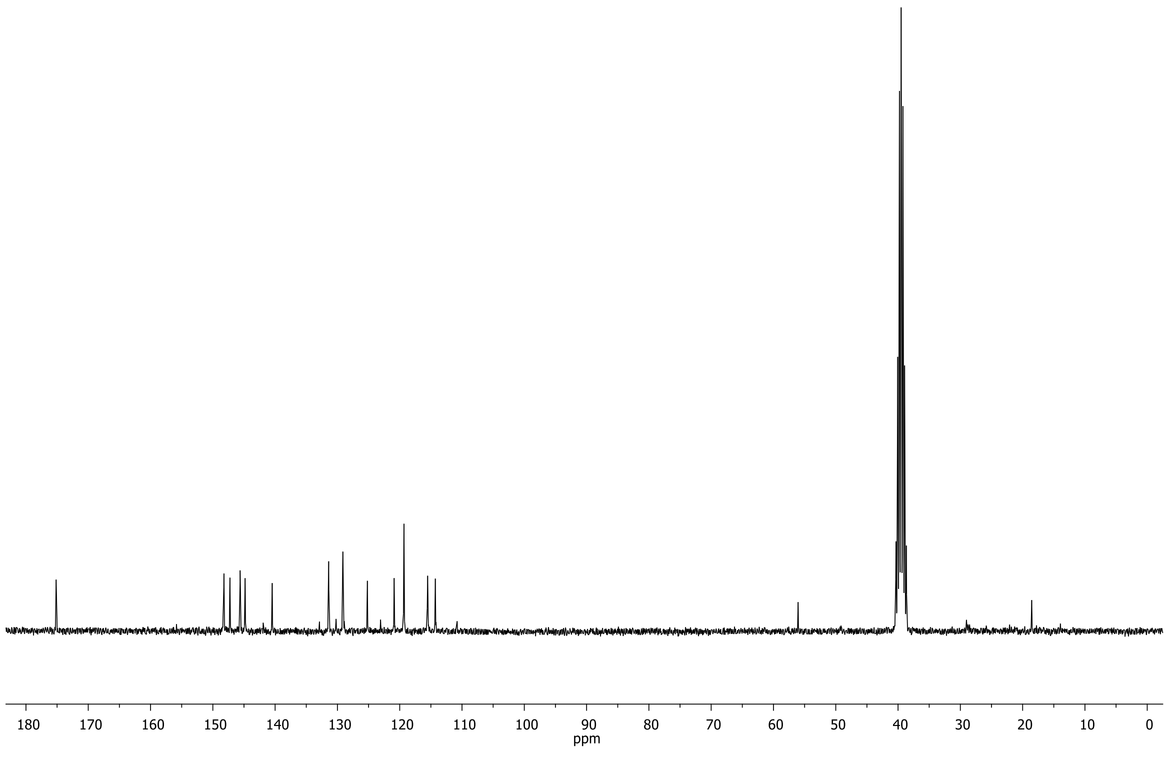

^{13}C-NMR (125.7 MHz, DMSO-d_6) of **58**

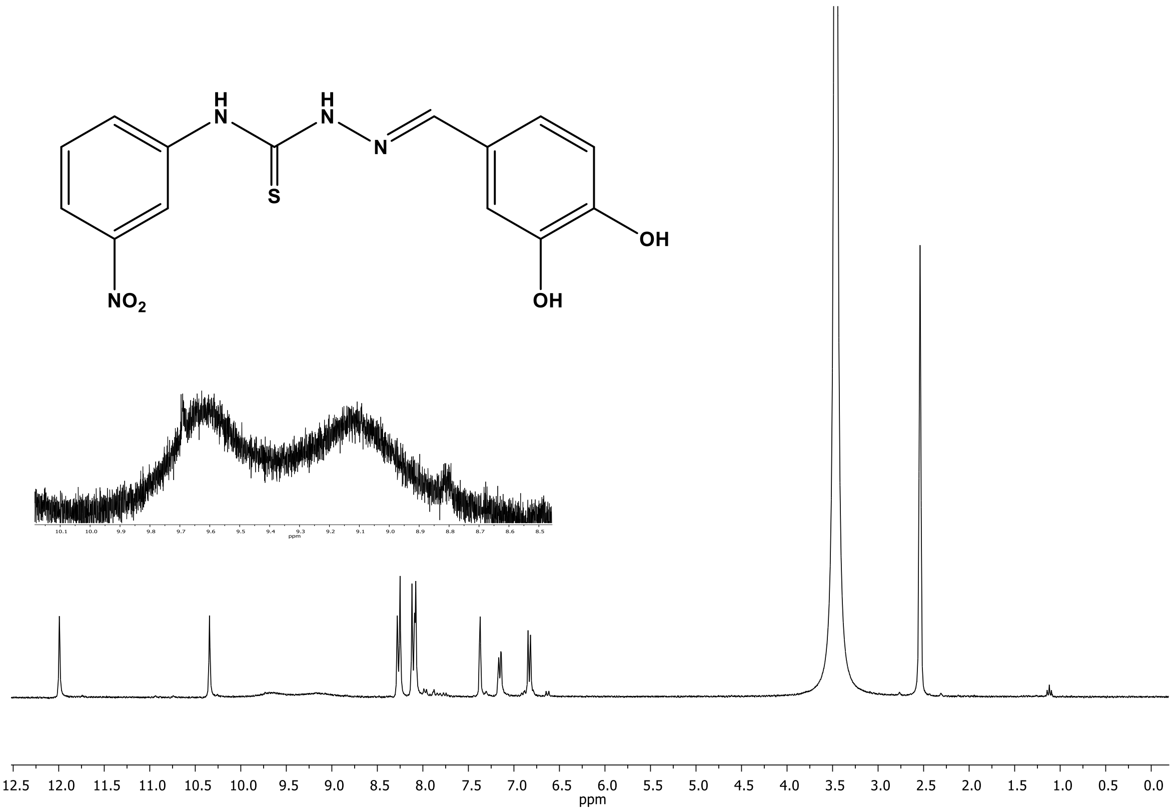

^{1}H-NM (300 MHz, DMSO-d_6) of **61**

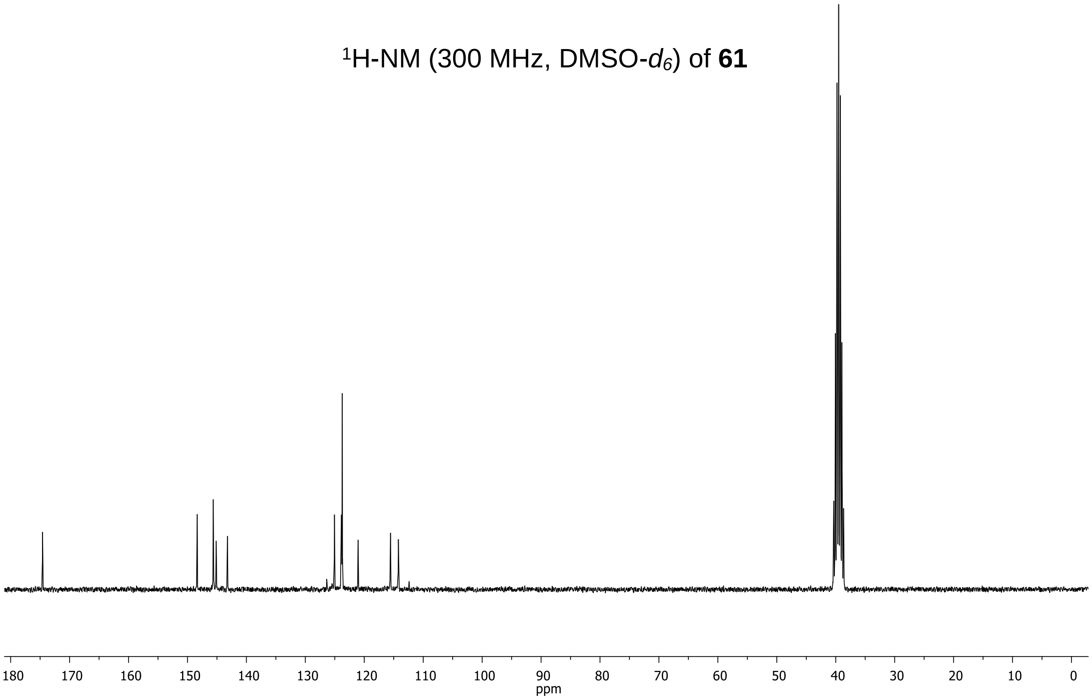

^{13}C-NMR (125.7 MHz, DMSO-d_6) of **61**

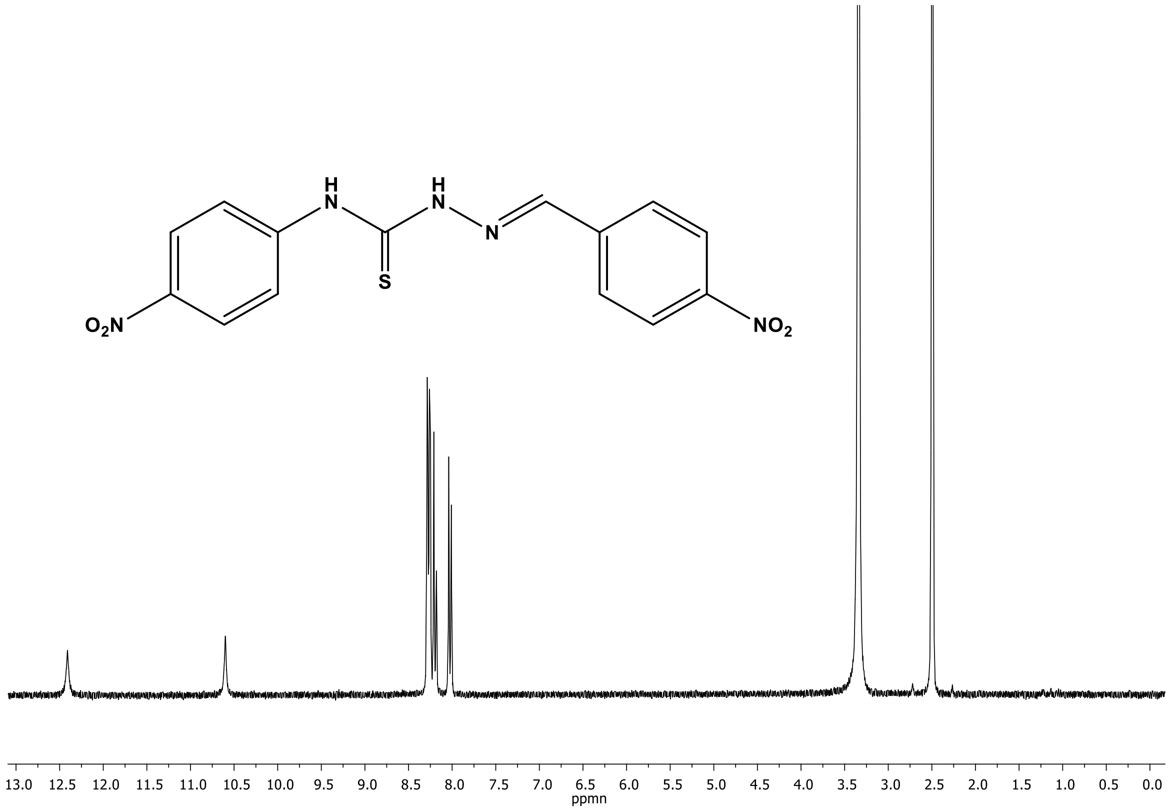

^{1}H-NMR (300 MHz, DMSO-d_6) of **62**

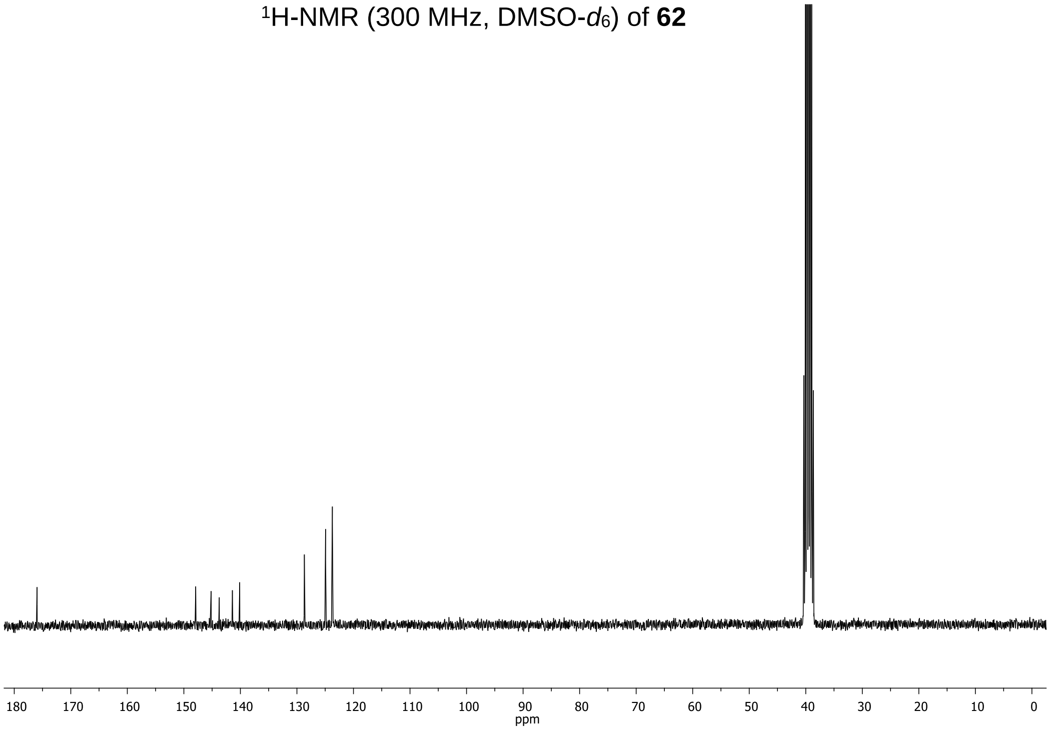

^{13}C-NMR (125.7 MHz, DMSO-d_6) of **62**

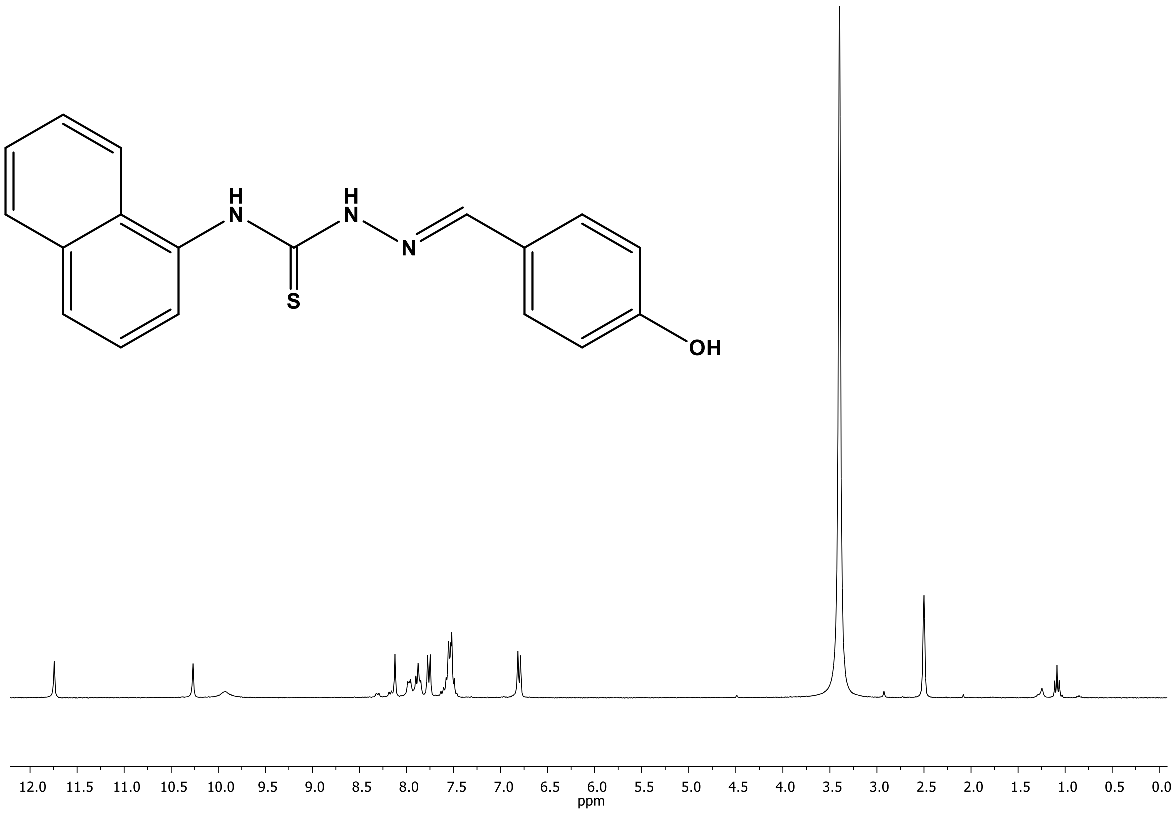

^{1}H-NMR (300 MHz, DMSO-d_6) of **63**

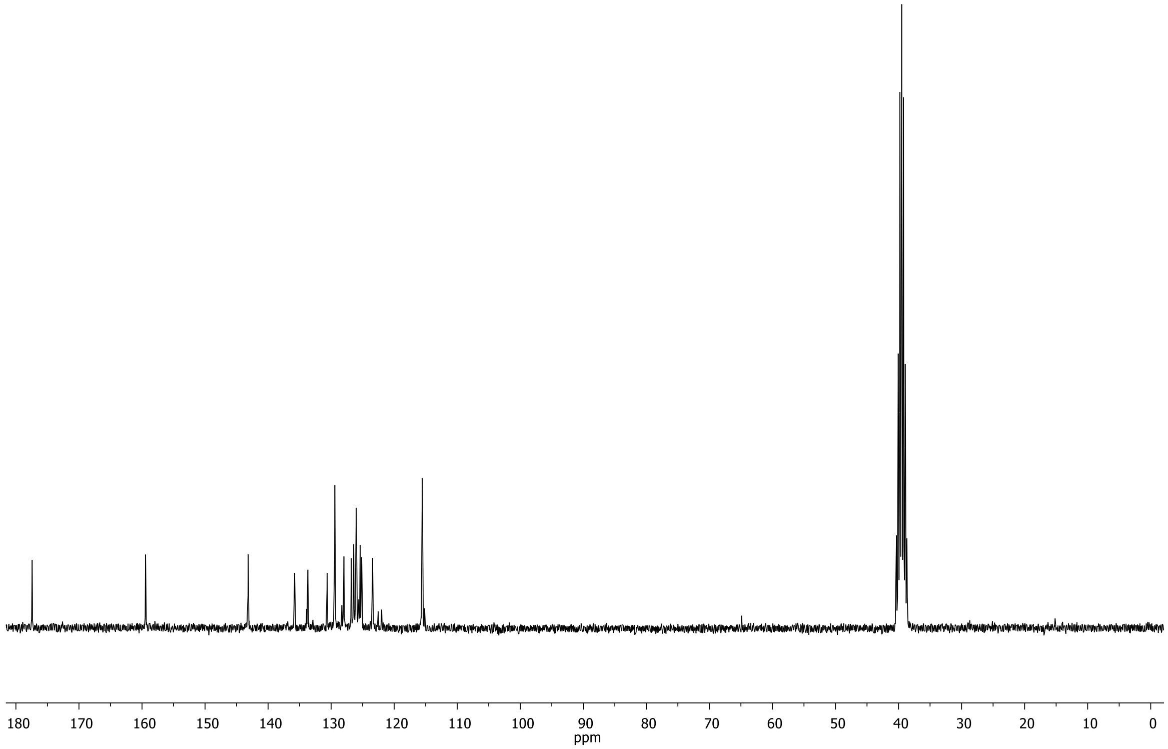

^{13}C-NMR (125.7 MHz, DMSO-d_6) of **63**

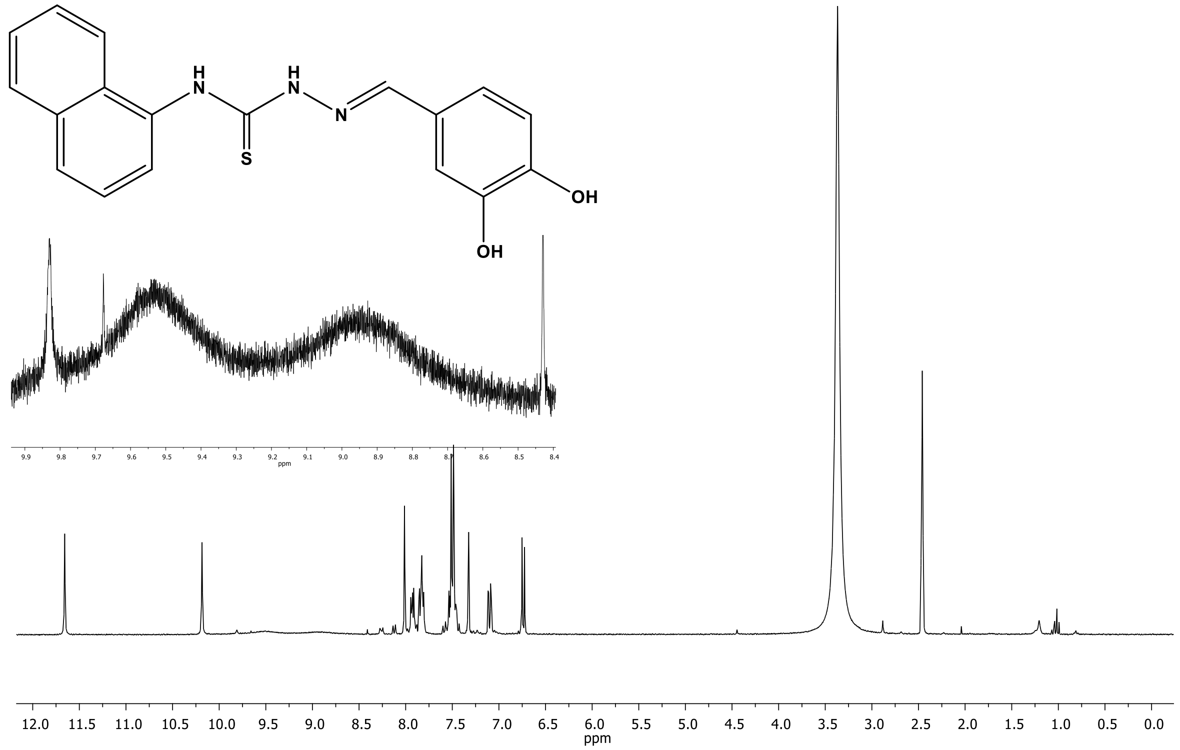

^{1}H-NMR (300 MHz, DMSO-d_6) of **65**

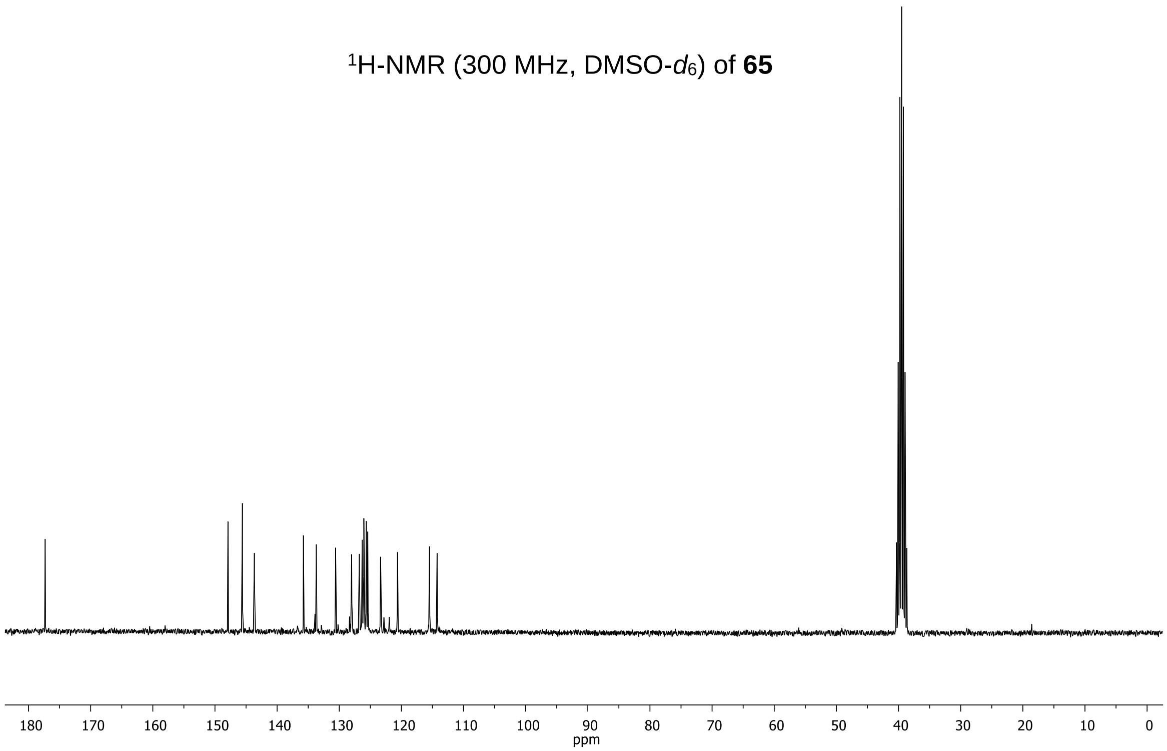

^{13}C-NMR (125.7 MHz, DMSO-d_6) of **65**

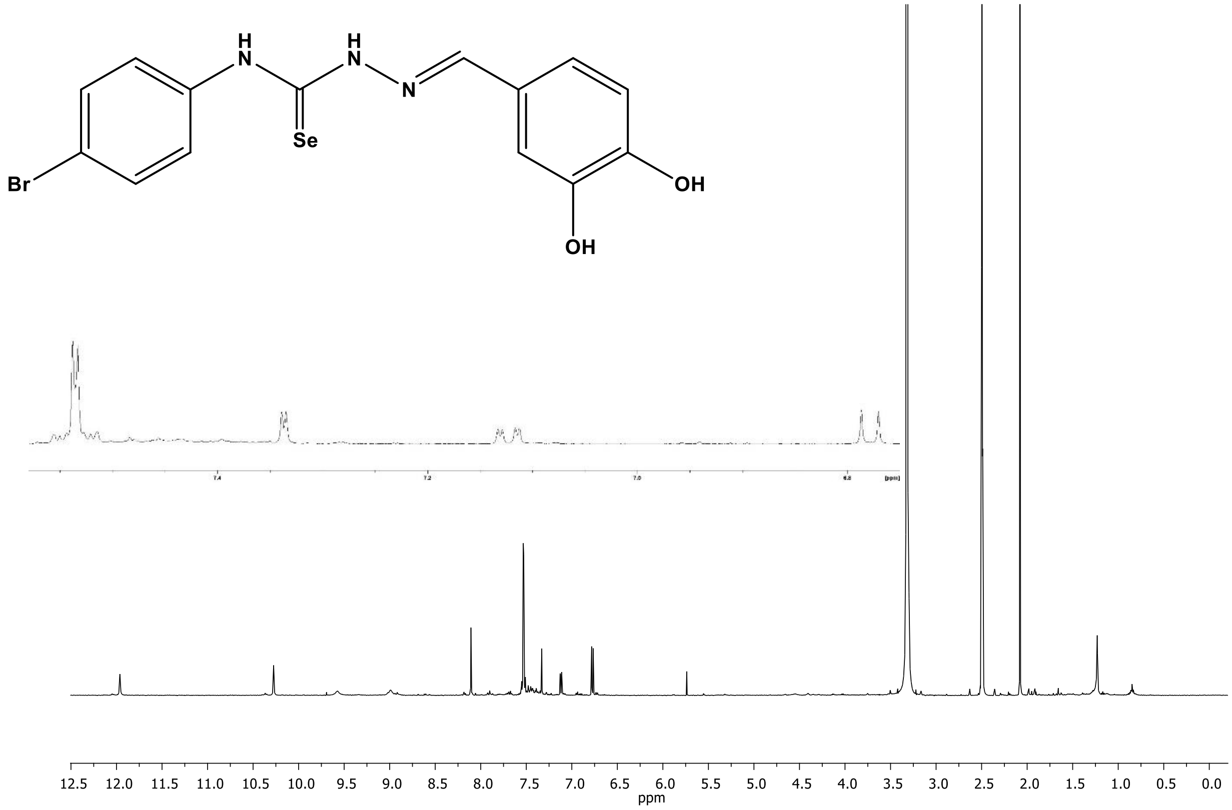

^{1}H-NMR (300 MHz, DMSO-d_6) of **74**

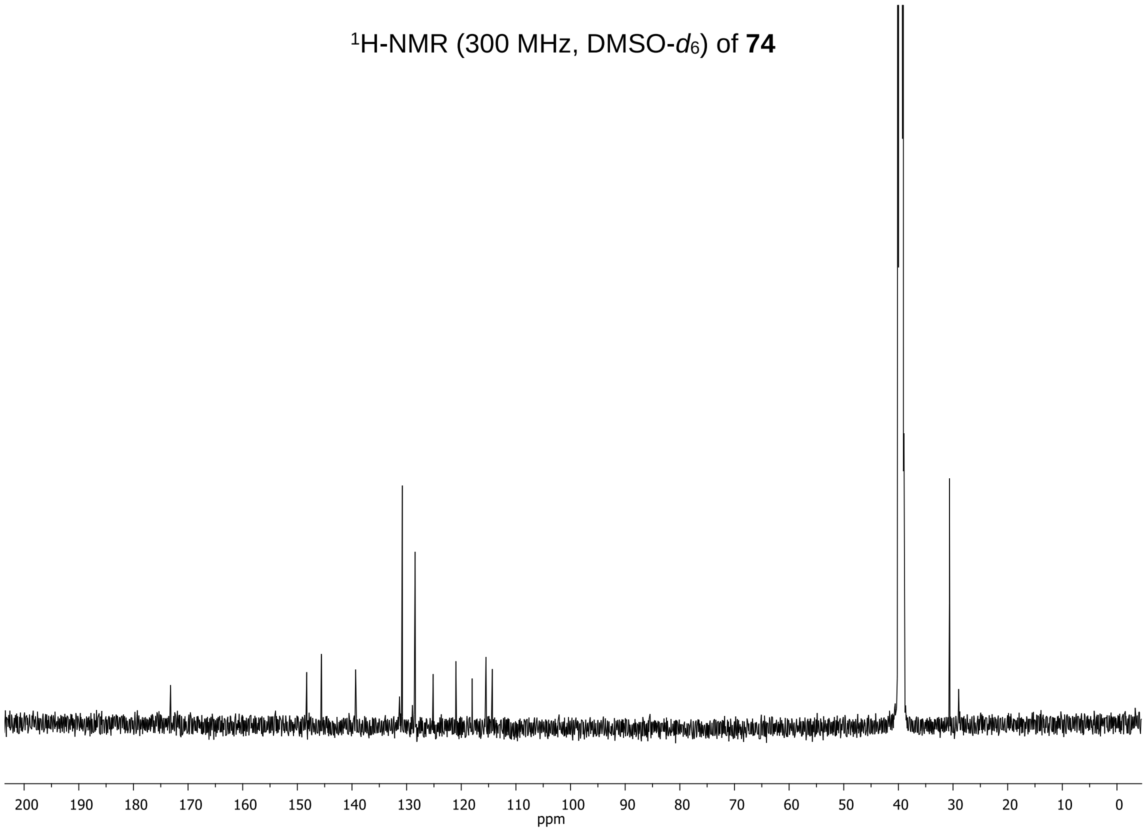

^{13}C-NMR (125.7 MHz, DMSO-d_6) of **74**

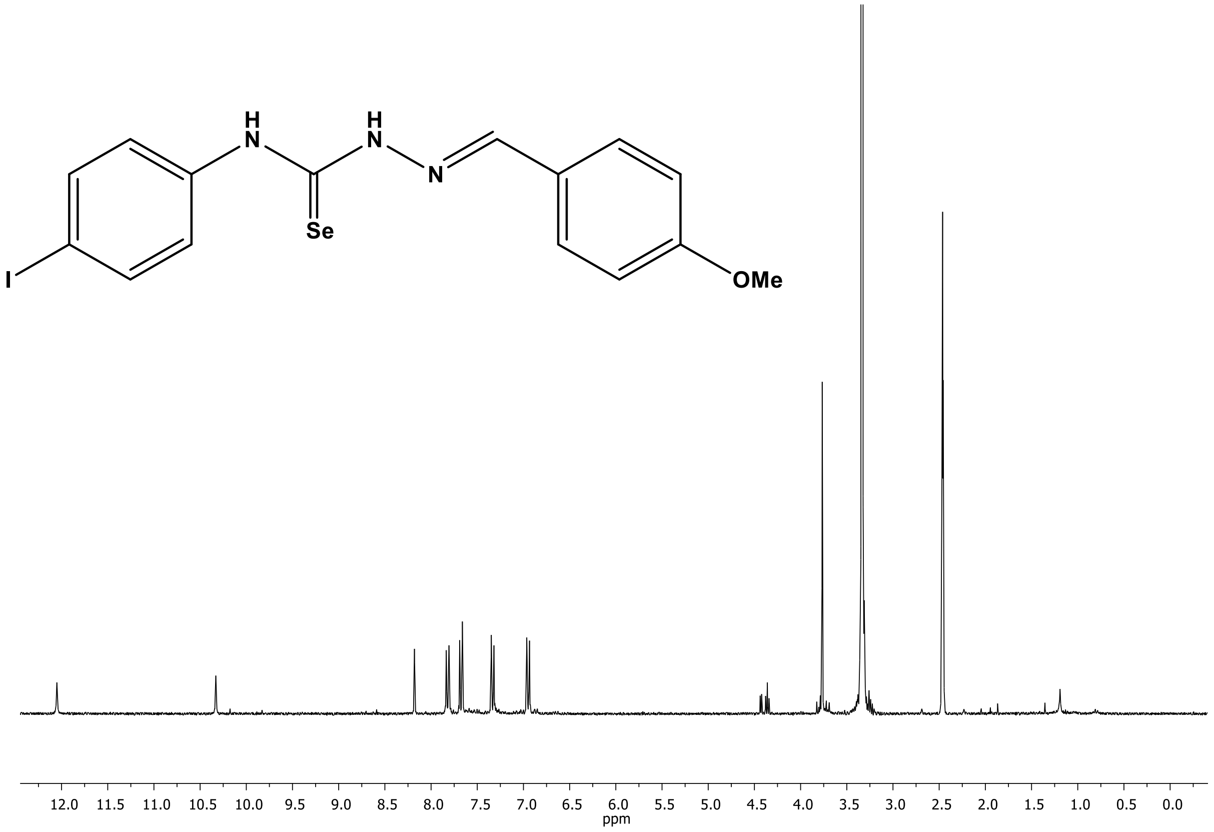

^{1}H-NMR (300 MHz, DMSO-d_6) of **75**

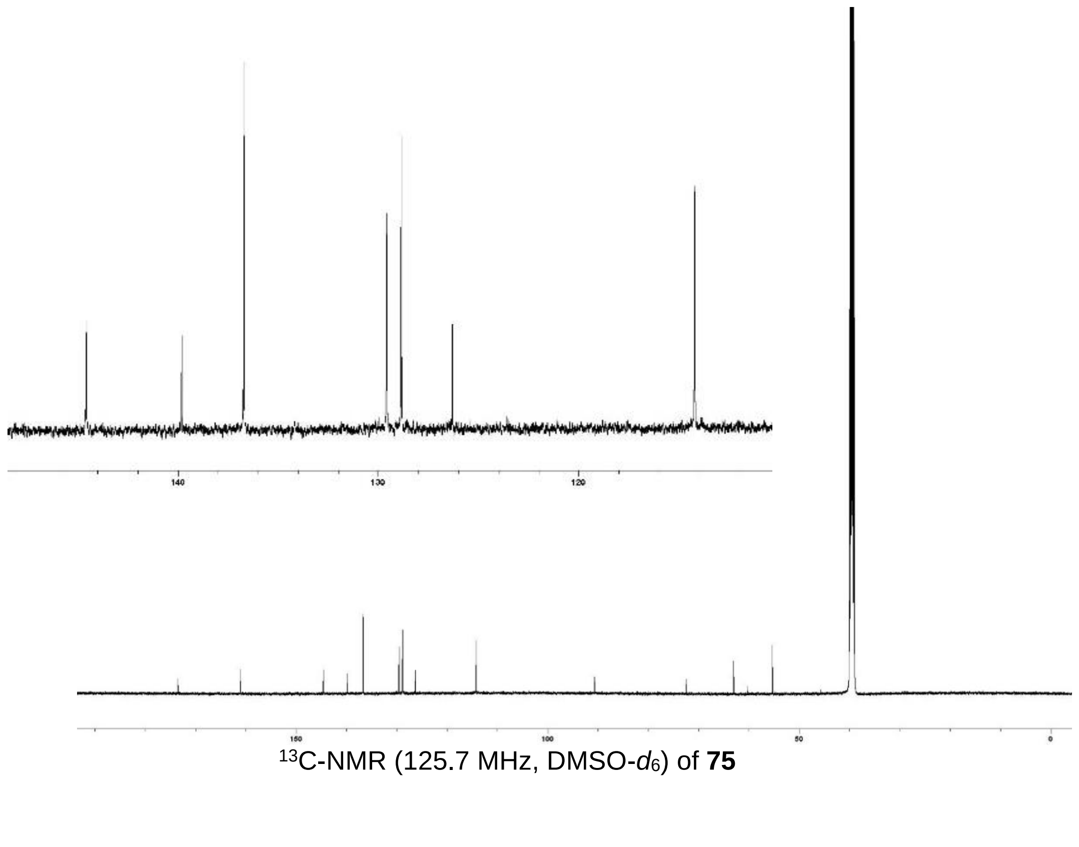

^{13}C-NMR (125.7 MHz, DMSO-d_6) of **75**

References

1. Fonseca-Berzal C, Ibáñez-Escribano A, Reviriego F, Cumella J, Morales P, Jagerovic N, Nogal-Ruiz JJ, Escario JA, da Silva PB, Soeiro MNC, Gómez-Barrio A, Arán VJ. Antichagasic and trichomonacidal activity of 1-substituted 2-benzyl-5-nitroindazolin-3-ones and 3-alkoxy-2-benzyl-5-nitro-2*H*-indazoles. Eur J Med Chem. 2016;115:295–310.
2. Fonseca-Berzal C, Ibáñez-Escribano A, Vela N, Cumella J, Nogal-Ruiz JJ, Escario JA, da Silva PB, Batista MM, Soeiro MNC, Sifontes-Rodríguez S, Meneses-Marcel A, Gómez-Barrio A, Arán VJ. Antichagasic, leishmanicidal, and trichomonacidal activity of 2-benzyl-5-nitroindazole-derived amines. ChemMedChem. 2018;13:1246–1259.
3. Ibáñez Escribano A, Meneses Marcel A, Machado Tugores Y, Nogal Ruiz JJ, Arán Redó VJ, Escario García-Trevijano JA, Gómez Barrio A. Validation of a modified fluorimetric assay for the screening of trichomonacidal drugs. Mem Inst Oswaldo Cruz. 2012;107:637–643.
4. Fonseca-Berzal C, Merchán Arenas DR, Romero Bohórquez AR, Escario JA, Kouznetsov VV, Gómez-Barrio A. Selective activity of 2,4-diaryl-1,2,3,4-tetrahydroquinolines on *Trypanosoma cruzi* epimastigotes and amastigotes expressing β-galactosidase. Bioorg Med Chem Lett. 2013;23:4851–4856.
5. Buckner FS, Verlinde CLMJ, La Flamme AC, Van Voorhis WC. Efficient technique for screening drugs for activity against *Trypanosoma cruzi* using parasites expressing β-galactosidase. Antimicrob Agents Chemother. 1996;40:2592–2597.
6. Silva LH, Nussenzweig, V. Sobre uma cepa de *Trypanosoma cruzi* altamente virulenta para o camundongo branco. Folha Clin Biol. 1953;20:191–207.
7. Zingales B, Andrade SG, Briones MRS, Campbell DA, Chiari E, Fernandes O, Guhl G, Lages-Silva E, Macedo AM, Machado CR, Miles MA, Romanha AJ, Sturm NR, Tibayrenc M, Schijman AG. A new consensus for *Trypanosoma cruzi* intraspecific nomenclature: second revision meeting recommends TcI to TcVI. Mem Inst Oswaldo Cruz. 2009;104:1051–1054.
8. Vega MC, Rolón M, Montero-Torres A, Fonseca-Berzal C, Escario JA, Gómez-Barrio A, Gálvez J, Marrero-Ponce Y, Arán VJ. Synthesis, biological evaluation and chemometric analysis of indazole derivatives. 1,2- disubstituted 5-nitroindazolinones, new prototypes of antichagasic drug. Eur J Med Chem. 2012;58:214–227.
9. Vega C, Rolón M, Martínez-Fernández AR, Escario JA, Gómez-Barrio A. A new pharmacological screening assay with *Trypanosoma cruzi* epimastigotes expressing β-galactosidase. Parasitol Res. 2005;95:296–298.

10. Rolón M, Vega C, Escario JA, Gómez-Barrio A. Development of resazurin microtiter assay for drug sensibility testing of *Trypanosoma cruzi* epimastigotes. Parasitol. Res. 2006;99:103–107.

11. Fonseca-Berzal C, da Silva CF, Batista DGJ, de Oliveira GM, Cumella J, Batista MM, Peres RB, Nefertiti ASG, Escario JA, Gómez-Barrio A, Arán VJ, Soeiro MNC. Activity profile of two 5-nitroindazole derivatives over the moderately drug-resistant *Trypanosoma cruzi* Y strain (DTU TcII): *in vitro* and *in vivo* studies. Parasitology. 2020;147:1216–1228.

12. Ibáñez-Escribano A, Meneses-Marcel A, Marrero-Ponce Y, Nogal-Ruiz JJ, Arán VJ, Gómez-Barrio A, Escario JA. A sequential procedure for rapid and accurate identification of putative trichomonacidal agents. J Microbiol Methods. 2014;105:162–167.

13. Ibáñez-Escribano A, Reviriego F, Nogal-Ruiz JJ, Meneses-Marcel A, Gómez-Barrio A, Escario JA, Arán VJ. Synthesis and in vitro and in vivo biological evaluation of substituted nitroquinoxalin-2-ones and 2,3-diones as novel trichomonacidal agents. Eur J Med Chem. 2015;94:276–283.

14. Ibáñez-Escribano A, Reviriego F, Vela N, Fonseca-Berzal C, Nogal-Ruiz JJ, Arán VJ, Escario JA, Gómez-Barrio A. Promising hit compounds against resistant trichomoniasis: synthesis and antiparasitic activity of 3-(ω-aminoalkoxy)-1-benzyl-5-nitroindazoles. Bioorg Med Chem Lett. 2021;37:127843.

15. Vilela R, Menna-Barreto RFS, Benchimol M. Methyl jasmonate induces cell death and loss of hydrogenosomal membrane potential in *Trichomonas vaginalis*. Parasitol Int. 2010;59:387–393.
